# The *Dictyostelium* Kinome—Analysis of the Protein Kinases from a Simple Model Organism

**DOI:** 10.1371/journal.pgen.0020038

**Published:** 2006-03-31

**Authors:** Jonathan M Goldberg, Gerard Manning, Allen Liu, Petra Fey, Karen E Pilcher, Yanji Xu, Janet L Smith

**Affiliations:** 1 Boston Biomedical Research Institute, Watertown, Massachusetts, United States of America; 2 Razavi-Newman Center for Bioinformatics, Salk Institute for Biological Studies, La Jolla, California, United States of America; 3 Center for Genetic Medicine, Northwestern University, Chicago, Illinois, United States of America; Yale University, United States of America

## Abstract

Dictyostelium discoideum is a widely studied model organism with both unicellular and multicellular forms in its developmental cycle. The *Dictyostelium* genome encodes 285 predicted protein kinases, similar to the count of the much more advanced *Drosophila*. It contains members of most kinase classes shared by fungi and metazoans, as well as many previously thought to be metazoan specific, indicating that they have been secondarily lost from the fungal lineage. This includes the entire tyrosine kinase–like (TKL) group, which is expanded in *Dictyostelium* and includes several novel receptor kinases. *Dictyostelium* lacks tyrosine kinase group kinases, and most tyrosine phosphorylation appears to be mediated by TKL kinases. About half of *Dictyostelium* kinases occur in subfamilies not present in yeast or metazoa, suggesting that protein kinases have played key roles in the adaptation of *Dictyostelium* to its habitat. This study offers insights into kinase evolution and provides a focus for signaling analysis in this system.

## Synopsis

Protein kinases are eukaryotic enzymes involved in cell communication pathways, and transmit information from outside the cell or between subcellular components within the cell. About 2.5% of genes code for protein kinases, and mutations in many of these cause human disease. The authors characterize the complete set of protein kinases (kinome) from *Dictyostelium discoideum,* a social amoeba that responds to starvation by forming aggregates of cells, which then differentiate into multicellular fruiting bodies. *Dictyostelium* branched from the vertebrate lineage after plants but before fungi, and thus illuminates an interesting period in evolutionary history. By comparing the *Dictyostelium* kinome to those of other organisms, the authors find 46 types of kinases that appear to be conserved in all organisms, and are likely to be involved in fundamental cellular processes. *Dictyostelium* is an established model organism for studying many aspects of cell biology that are conserved in humans, and this exposition of conserved kinases will help to guide future studies. The *Dictyostelium* kinome also contains an impressive degree of creativity—almost half of the kinases are unique to *Dictyostelium*. Many of these *Dictyostelium*-specific kinases may be related to this organism's distinctive mechanism for coping with starvation.

## Introduction

Dictyostelium discoideum amoebae thrive in moist soil, where they consume smaller microbes. Nutritional stress drives cells to aggregate by means of chemotactic signals, and these aggregates then differentiate into multicellular fruiting bodies containing spores. Thus, *Dictyostelium* exemplifies many processes characteristic of complex eukaryotes, including phagocytosis, autophagy, chemotaxis, motility, adhesion, and cell-type differentiation. *Dictyostelium* branched from the lineage that ultimately led to the metazoa before yeast but after plants, and while many of its molecular mechanisms are remarkably similar to those in humans, it also has a number of unique processes that apparently evolved after it diverged from the fungal/metazoan lineage [1,2].

As a model organism, *Dictyostelium* provides an appealing balance of interesting biological problems and experimental tractability. *Dictyostelium* offers both traditional and molecular genetics, including targeted gene disruption techniques, restriction enzyme–mediated integration mutagenesis, and RNAi [3–5]. Although normally haploid, parasexual genetic techniques are available for generating diploids [6]. Biochemical studies are facilitated by the ability to grow cells in large amounts. The recently published genome encodes approximately 12,500 protein-coding genes [2], and although this is more than twice the number of genes in yeast, it is only about half that of humans, and the rarity of alternative splicing further simplifies the proteome compared with those of vertebrates. The developmental transcription profile of more than half of these genes has been determined, providing an estimate of their roles in developmental signaling and vegetative growth [7]. The availability of complete genomic data has already synergized with genetic and proteomic approaches [8–10], and enables many other new approaches, including genome-scale knock-out, knock-down, and overexpression studies, as well as the rapid identification by mass spectrometry of proteins in interaction studies.

Protein kinases are key post-transcriptional regulators of most cellular processes, and are particularly involved in signal transduction and coordination of complex pathways. Almost all protein kinases have catalytic domains belonging to the eukaryotic protein kinase (ePK) superfamily, and share a common ancestry and fold. Despite these similarities they interact specifically with a remarkable variety of effectors and substrates as a result of sequence differences within and outside of their ePK domains. A number of other unrelated enzymes also have protein kinase activity, and are referred to as atypical protein kinases (aPKs).

Approximately 44 *Dictyostelium* kinases have been the subject of targeted gene disruption studies, and in almost every case defects are observed, with many kinases having important functions during development (reviewed in [11]). Here we determine the complete kinase catalog (kinome) of *Dictyostelium,* and analyze it in the context of the complete kinomes of yeast and metazoa. Classification of kinases into groups, families, and subfamilies reveals many kinases that are conserved in other organisms, and many others that are *Dictyostelium* specific, indicating that protein phosphorylation pathways have important functions in unique aspects of *Dictyostelium* biology. Since *Dictyostelium* branched from the metazoan lineage before yeast, our analysis sheds light on the early evolution of metazoan kinases. Many metazoan kinases that are missing from yeast are present in *Dictyostelium,* indicating that they are more ancient than previously thought. For some of these, such as DNAPK (see Table S1 for kinase acryonyms) and PEK, *Dictyostelium* provides a unique opportunity to study important disease-related proteins in a simple, tractable model organism.

## Results/Discussion

### Discovery and Overview of *Dictyostelium* Protein Kinases

Comprehensive searches of *Dictyostelium* genomic and expressed sequences were carried out using profiles for the conserved ePK domain, individual ePKs, and both profiles and sequences for additional aPKs and histidine kinases (HisKs). These searches yielded a catalog of 285 candidate protein kinases (246 ePKs, 26 aPKs, and 14 HisKs, one of which is also an ePK) and 15 candidate ePK pseudogenes. Protein sequences, accession numbers, and other information on the kinome are summarized in Table S2. This final catalog strongly overlaps, but does differ from, the catalog of kinases reported by Kimmel [11]. All kinase gene predictions were inspected, and 20% were edited to give a more plausible gene model. Eleven genes are found in an identical second copy, owing to a recent chromosomal duplication of a portion of Chromosome 2 in the sequenced strain of *Dictyostelium,* and are not included in the count of 285. Seven sequences have very limited similarity to known kinase domains, and may be considered borderline members of the kinome. These are: LvsG, DDB0230007, DDB0229337, DDB0229346, and three of the four atypical actin-fragmin kinases (DDB0190004, DDB0190056, and DDB0187708). Almost half of the kinase genes (127) have been analyzed in large-scale developmental profiling studies [12,13], and 187 have expressed sequence tags (ESTs), with a median number of five. The 285 *Dictyostelium* protein kinase genes represent 2.6% of the genome, comparable to 2.3% of yeast and human genes ([14,15]; G. Manning, unpublished data).

**Figure 1:**
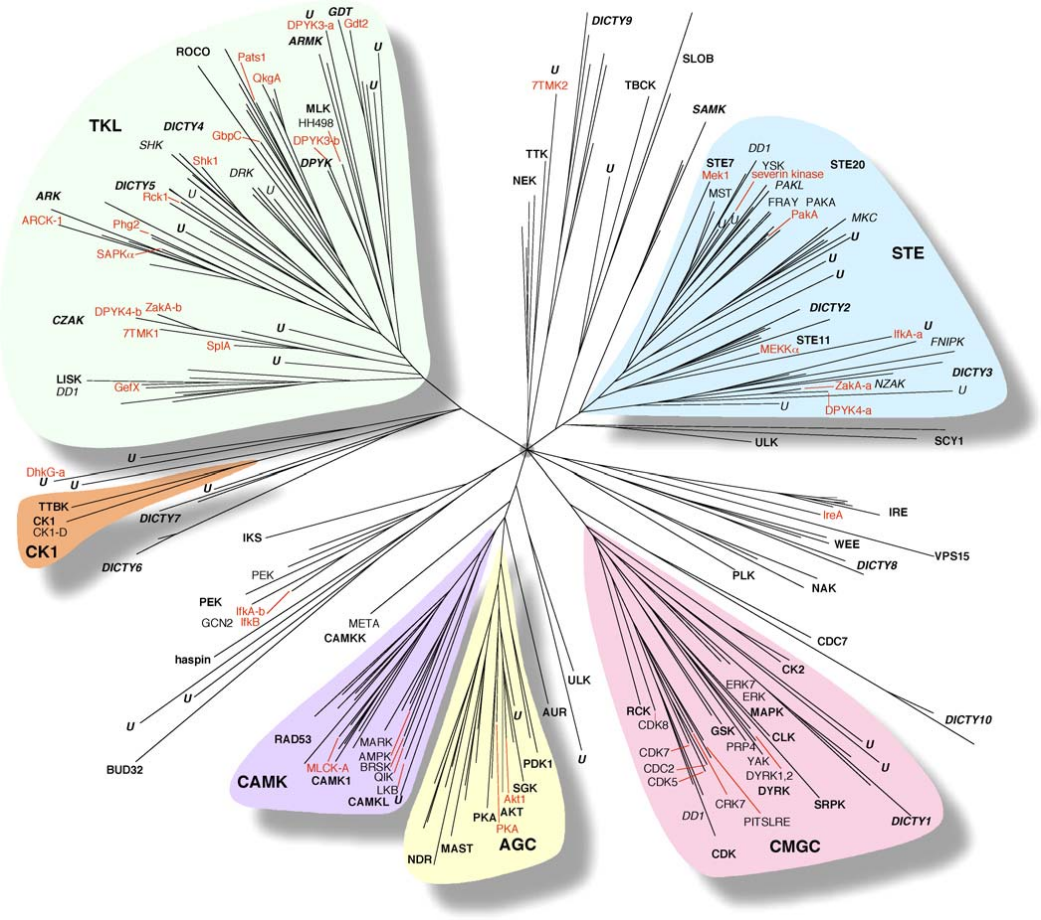
Tree of the *Dictyostelium* Kinome A tree of 248 *Dictyostelium* ePK domains is presented. Pseudogenes, Chromosome 2 duplications, and sequences with very divergent ePK domains were omitted. The N- and C-terminus domains of dual-domain kinases, respectively designated by an “-a” or “-b” extension, were analyzed independently. Group and family names are shown in bold type, subfamily names in plain type, and *Dictyostelium*-specific families and subfamilies in italic. Selected protein names are shown in red. Branch lengths reflect relative distances between ePK domains. A branching order could not be assigned in the region indicated by the small gray circle because of the diversity of the sequences.

The *Dictyostelium* ePK domains were aligned (Figure S1), and the relationship between them is shown in a tree (Figures 1 and S2, Dataset S1). The tree indicates that the AGC and CAMK groups, named for named for protein kinases A, G, and C, and Ca^2+^/calmodulin (CaM)–activated kinases, respectively, are the most closely related groups in *Dictyostelium,* while the tyrosine kinase–like (TKL) and cell or casein kinase 1 (CK1) groups are the most distant from other groups.

**Figure 2:**
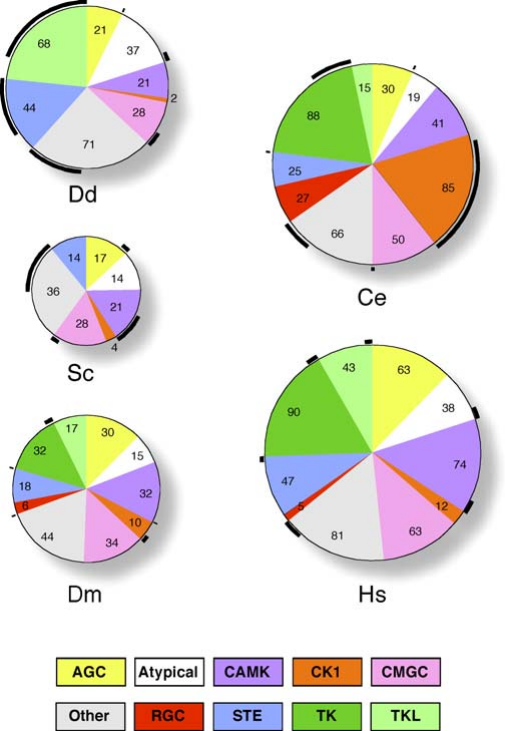
Group-Level Comparison of the *Dictyostelium* and Other Kinomes Pie charts depict the proportion of the kinome that is devoted to the major groups in *Dictyostelium,* yeast, worm, flies, and humans. The size of the pie is proportional to the number of kinase domains in each organism, and the total number of kinases in each group is shown in the slice. Data for other organisms are from KinBase (http://kinase.com). The portion of each group that is in families or subfamilies not found in the other four kinomes is indicated with a black arc drawn outside the slice for that group.

Kinases are not a monolithic family, but rather have very distinct functions that are often conserved across evolution. Using pairwise and multiple sequence alignments, tree analysis and subfamily hidden Markov models (HMMs), we classified all *Dictyostelium* kinases to a hierarchical system of groups, families, and subfamilies (Table S2) [15–17]. Classifying the *Dictyostelium* kinases in this way enabled us to compare orthologous sets of kinases over large evolutionary distances, where a one-to-one orthologous relationship between proteins usually does not exist. This analysis allowed us to discern the likely evolutionary lineages of the *Dictyostelium* kinases and to assign possible functions to unstudied kinase genes. At the broadest level of kinase classification several conclusions can be made (Figure 2). Most notably, *Dictyostelium* lacks kinases in the TK group, but has an expanded TKL group, whereas yeast lacks both these groups. The CK1 group, which is greatly expanded in worms, is reduced in *Dictyostelium,* with only two members. The receptor guanylyl cyclase (RGC) group is absent from *Dictyostelium* and yeast, thereby retaining its metazoan specificity.

A core set of 46 kinase subfamilies is conserved in *Dictyostelium,* yeast, and throughout the metazoa (Table S3A). (In counts such as this, we use the term “subfamily” to indicate a set of related kinases at the deepest level of classification. In some cases, families are not further classified into subfamilies, and the family is counted.) The widespread conservation of these kinases suggests that they play critical roles in fundamental cellular processes. These “universal” kinases mediate functions such as lipid signaling (AKT and PDK1), MAPK cascades (ERK, STE7, STE11, STE20), cell-cycle control (WEE, CDC2, CDC7, CDK7, CDK8, CRK7), mitosis (BUB, NEK, AUR, SCY1, PLK, Haspin), DNA damage control (RAD53, ATR), and energy homeostasis (TOR, AMPK, GCN2).

**Figure 3:**
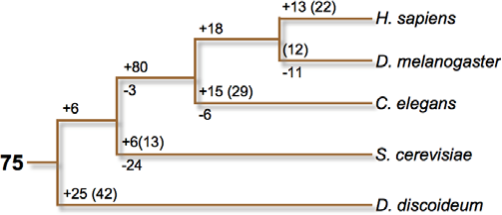
Summary of Kinase Subfamily Invention and Loss Comparison of *Dictyostelium* with four other kinomes suggests that 75 distinct subfamilies existed in their common ancestor, and that new subfamilies were born (positive numbers) and lost (negative numbers) in most lineages. Numbers in parenthesis indicate “unique” kinases within each lineage that may be classified as novel subfamilies when more kinomes are analyzed. Most notably, S. cerevisiae has lost 24 subfamilies present in the common ancestor, while metazoans invented an additional 80 conserved subfamilies.

The remaining *Dictyostelium* kinases show evolutionary distributions reflecting scenarios of gene expansion, divergence, and loss. Most dramatically, *Dictyostelium* has 24 kinase subfamilies that occur in metazoan kinomes but not in yeast (Table 1). This implies that yeast lost these kinases in pursuit of a more specialized biological program. Noteworthy in this set are the G11 and DNAPK aPKs, which had previously only been observed in vertebrates. Six subfamilies are found in yeast and metazoa, but are missing from *Dictyostelium* (Table 2). Some of these presumably arose later in evolution, and others may have been lost in *Dictyostelium*. Many additional families still appear to be metazoan-specific (Table S3H). These include many well-known kinases, such as tyrosine kinases, the stress-responsive MAPKs p38 and JNK, CAMK2, MLCK, STKR (which includes TGFβ receptors), and RAF. Conversely, three types of kinases are found in yeast and *Dictyostelium* but were lost in metazoa: the YAK subfamily of DYRK kinases (CMGC group), IKS in the “Other” group, and histidine kinases (Table S3F). A summary of kinase invention and loss in the evolution of *Dictyostelium,* yeast, worms, flies, and humans is shown in Figure 3.

**Table 1:**
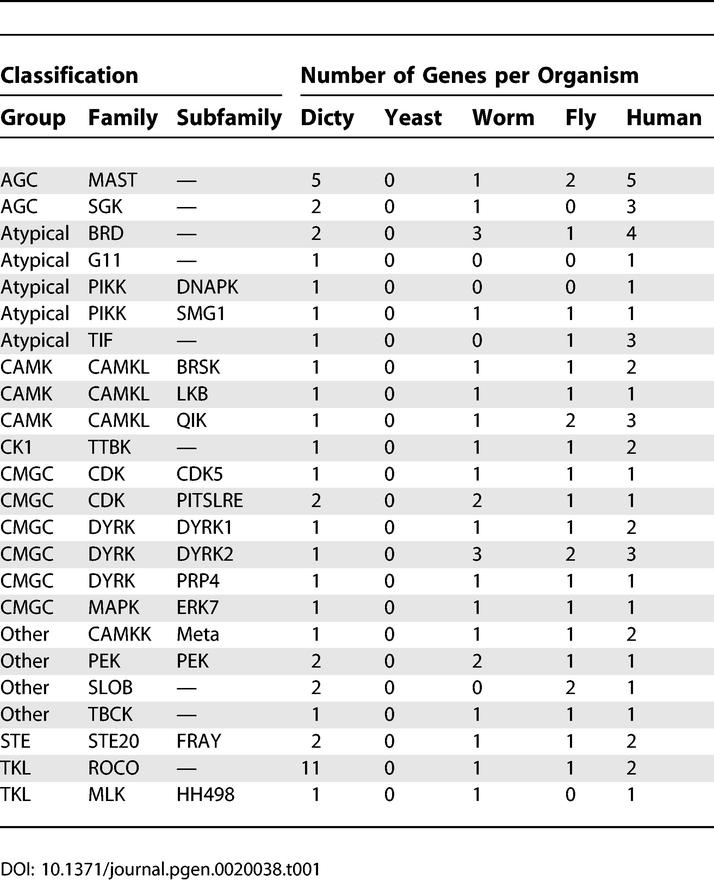
Kinases That Appear to Have Been Secondarily Lost from Yeast

**Table 2:**
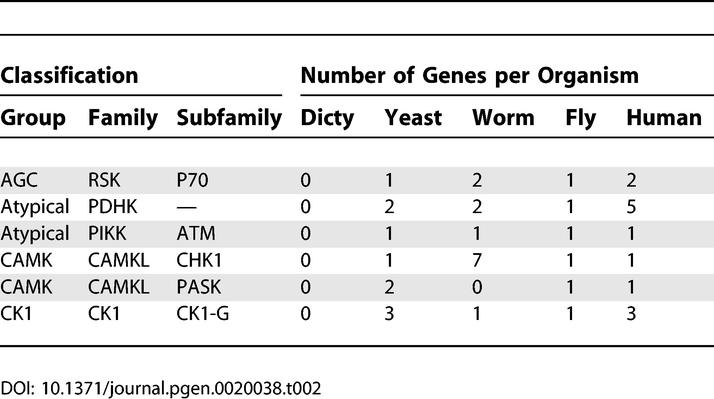
Broadly Conserved Kinases That Are Missing from *Dictyostelium*

About half of *Dictyostelium* kinases are singletons or in 25 subfamilies not found in yeast or metazoa (Figures 2 and 3; Table S3G). Orthologs for some of these “*Dictyostelium*-specific” kinases may be found as more kinomes are analyzed. Many of the *Dictyostelium*-specific kinases appear to be involved in unique aspects of *Dictyostelium* biology; for example, SplA and ZakA are involved in spore differentiation, and Gdt1 and 2 are involved in the growth-to-development transition [18–21]. All but 5 of the 140 *Dictyostelium*-specific kinases are in the TKL, STE, and Other groups. It is possible that members of these groups tended to be less involved in fundamental biological functions, compared to the AGC, CAMK, CK1, and CMGC groups. As a result, new genes in the former groups might have been less likely to have detrimental dominant-negative effects.

### Group-by-Group Analysis

The AGC group, named for protein kinases A, G, and C, consists of small, cytoplasmic kinases that mediate many aspects of phospholipid, cyclic nucleotide, and calcium signaling in eukaryotic cells. *Dictyostelium* has 21 AGC kinases, only two of which have been studied (PKA and AKT). We found two additional members of the PKA family, as well as members of the PDK1 and NDR families, which are broadly conserved, and SGK and MAST members, which were previously only found in metazoa. No protein kinase C (PKC) was found, but C1 domains, which are commonly found on PKC kinases, are present in three *Dictyostelium* kinases from the TKL (DDB0231197 and ARCK-1) and STE (PakD) groups, suggesting that DAG-regulated kinase signaling may exist in *Dictyostelium*. PKC is also absent from *Plasmodium* and *Arabidopsis,* but is found in yeast, suggesting that it evolved later. Conversely, p70 S6K is absent from *Dictyostelium* but present in yeast and *Arabidopsis,* and was presumably lost from the *Dictyostelium* lineage [22,23]. Several other families (PKG, GRK, YANK, DMPK, and PKN) remain metazoan-specific.

The CAMK group consists of Ca^2+^/CaM-activated kinases (CAMK1, CAMK2, DAPK, CASK, MLCK, and PHK families), and related kinases that are not CaM regulated, including the CAMK-like (CAMKL) and RAD53 families. *Dictyostelium* has 21 members of the CAMK group, and like yeast, all members are in the CAMK1, CAMKL, and RAD53 families. The CAMK1 family is expanded, with eight genes, compared to one to five in other organisms. The RAD53 family is also expanded, with five members, compared to one or two in other kinomes. One MLCK has been identified biochemically in *Dictyostelium* [24], but it is in the CAMK1 family, not the MLCK family. This may indicate that an early CAMK acted on myosin light chain, and different families kept that specificity in the two lineages. Of the four CAMKL subfamilies conserved in yeast, flies, worms, and humans, *Dictyostelium* has AMPK and MARK, but lacks CHK1 (whose function may be replaced by the expanded RAD53 family), and PASK. *Dictyostelium* also has three CAMKL subfamilies (BRSK, LKB, and QIK) found in all metazoan kinomes but absent from yeast.

The CK1 group consists in *Dictyostelium* of two proteins. CK1 is from the ubiquitous CK1 family, and DDB0216336 is from the previously metazoan-specific TTBK family. Like other CK1-group kinases, the *Dictyostelium* members have no additional domains. CK1 appears to be essential, and may play a role in DNA repair [25].

The CMGC group is named for the CDK, MAPK, GSK, and CLK families, and also includes DYRK, SRPK, CDKL, RCK, and CK2. Six CMGC subfamilies are found in *Dictyostelium* and metazoans, but not yeast. These are DYRK1, DYRK2, PRP4, ERK7, PITSLRE, and CDK5. Metazoan Dyrk1A, PRP4, and PITSLRE are linked to cyclinL2 and splicing, and their absence in yeast may reflect the rarity of splicing in yeast, whereas introns are common in *Dictyostelium* genes. The metazoan-specific subfamilies that are lacking in *Dictyostelium* include several involved in the cell cycle: CDK4, CDK9, and TAIRE, as well as the CDKL (CDK-like) family and the HIPK subfamily of DYRK. Yeast and *Dictyostelium* have a new DYRK subfamily, comprised of Yak1 in yeast and YakA in *Dictyostelium*. One *Dictyostelium*-specific family is found (Dicty1), as well as a *Dictyostelium*-specific subfamily of CDK (CDK-DD1).

The STE group is named for yeast sterile-phenotype kinases, and consists of kinases involved in MAPK activation and related kinases, many involved in regulation of the cytoskeleton. *Dictyostelium* has 43 STE kinases. It has just single members of the STE7/MAP2K and STE11/MAP3K families (Mek1 and Mekkα, respectively), but has 23 STE20/MAP4K kinases, 14 kinases in two *Dictyostelium*-specific families (Dicty2 and Dicty3), and four unique group members. The *Dictyostelium* STE20s include members from the widely conserved PAKA, MST, and YSK subfamilies, the previously metazoan-specific FRAY subfamily, the *Dictyostelium*-specific PAKL, MKC, and STE20-DD1 subfamilies, and two unique STE20 family members. The lone *Dictyostelium* YSK phosphorylates the gelsolin-like protein severin, in keeping with actin-related functions in the fly homolog [26,27]. The two members of the FRAY subfamily are uncharacterized, but metazoan FRAYs may couple osmotic or other stresses to PAK kinase activation [28]. The *Dictyostelium*-specific MKC subfamily kinases are most similar to PAKs in kinase sequence (Figure 1) and C-terminal placement of the domain (Figure S3), but lack PBD/CRIB domains (see the legend of Figure S3 for domain acronyms), which mediate binding and activation by small GTPases. MkcA mutants have a defect in sporulation, but the other five MKC members have not been biologically characterized [29]. All four *Dictyostelium* PAKA kinases have a PBD/CRIB domain, and the three that have been studied induce cytoskeletal changes [30–34]. PAKc contains a PH domain also seen in yeast PAKs, where the PBD/CRIB and PH domains are thought to integrate Cdc42 and PI4P signaling [35]. This structure found in primitive organisms may be analogous—but not homologous—to the metazoan-specific DMPK kinases (AGC group), which also have both domains and signal from small GTPases to myosin [36]. In addition to the PAKA subfamily kinases, *Dictyostelium* has four kinases with divergent but functional PBD/CRIB domains (F. J. Rivero Crespo, personal communication). These PAKs comprise the PAK-like (PAKL) subfamily, which is evolutionarily more similar to the MST and YSK subfamilies than to PAKA (Figures 1 and S2).

The TKL group kinases have sequences reminiscent of both tyrosine and ser/thr kinases, though they are known to act biochemically as ser/thr kinases. In metazoa, they include MAP3Ks (MLKs and RAF), ser/thr receptor kinases, and kinases involved in immunity (RIPK, IRAK) and cytoskeletal regulation. *Dictyostelium* has 66 TKLs, almost twice the proportion of the kinome compared to metazoa (Figure 2). The presence of TKLs in *Dictyostelium* (and plants; [37]) indicates that this group is ancient, and was lost in budding yeast. *Dictyostelium* has members of the LISK, MLK, and ROCO families, but lacks orthologs of RAF, STKR, IRAK, and RIPK. The nine LISK members comprise a *Dictyostelium*-specific subfamily, and the one MLK is in the HH498 subfamily found in vertebrates and worms. *Dictyostelium* has nine members of the ROCO family, which are homologous to the previously described metazoan LRRK family, as both contain a ras-like domain, and a domain C-terminal of the ras-like domain, and an array of leucine-rich repeats [38]. Curiously, the kinase domains are divergent enough to not be sufficient to unite the two families on their own. Mutations in LRRK2 were recently found to be associated with Parkinson's disease [39,40]. *Dictyostelium* is the only organism for which the molecular role of ROCO/LRRK kinases has been extensively investigated. Pats1 plays a role in cytokinesis, GbpC mediates cGMP signaling during chemotaxis, and QkgA is involved in growth and aggregation [41–43]. Of the remaining 45 TKLs six are unclassified (TKL-Unique), and the rest are in six *Dictyostelium*-specific families (ARMK, ARK, Dicty4, Dicty5, CZAK, and GDT).

The Other group contains all ePKs that do not otherwise fit into a group. For the most part, families of the Other group are either unrelated or weakly related to each other or to the major ePK groups, although strong intrafamily similarity is common (Figures 1, S1, and S2). Three families are closely related to a major kinase group: the *Dictyostelium*-specific SAMK family is related to the STE group, aurora kinase in the AUR family branches near the AGC group, and DDB0220010 in the CAMKK family branches near the CAMK group. Many (18) of the families in the Other group are broadly conserved, being found in *Dictyostelium* and most other kinomes. Among these are kinases involved in cell division (AUR, PLK, BUB, NEK, CDC7, and WEE), the unfolded protein response (IRE), amino-acid starvation (GCN2), Ca^2+^ signaling (CAMKK), autophagy (ULK), protein sorting (VPS15), and actin regulation (NAK). The overall high frequency of divergent kinases in *Dictyostelium* is reflected in the Other group; *Dictyostelium* has 15 kinases in six *Dictyostelium*-specific families, and 13 Other-Unique kinases, with little similarity to any other kinase.

### Atypical Protein Kinases

While the ePK domain has been tremendously successful throughout eukaryotic evolution, several other smaller families have also been shown to have protein kinase activity (reviewed in [15]). These aPKs are a diverse assortment of proteins. Some have structural or residual sequence similarity to ePKs, while others have distinct structures and catalytic mechanisms. In many cases, biochemical data are scant, and reported kinase activity may even be the result of biochemical contaminants.

*Dictyostelium* has 26 aPKs, among which are representatives of the evolutionarily highly conserved ABC1, PIKK, RIO, and TAF families. *Dictyostelium* also has representatives of the Alpha, G11, TIF, and BRD kinase families; their presence indicates that they were lost in yeast. Several aPK families are absent from *Dictyostelium* and yeast, reinforcing the conclusion that they are specific to vertebrates (BCR, FASTK, and H11). *Dictyostelium* also lacks pyruvate dehydrogenase kinase (PDHK), a mitochondrial aPK that is otherwise broadly conserved. PDHK inhibits pyruvate dehydrogenase in response to insulin and fasting (reviewed in [44]). Since PDHK orthologs are found in plants, it appears that *Dictyostelium* lost the *PDHK* gene, perhaps in favor of its own unique program of coping with starvation conditions by forming fruiting bodies. *Dictyostelium* also has four homologs of actin-fragmin kinase, though only one conserves all the putative catalytic residues. This type of aPK has been observed to date only in *Physarum* [45].

Alpha kinases were discovered in *Dictyostelium* [46], and are found in vertebrates and worms, but not flies or budding yeast. Four of the six *Dictyostelium* alpha kinases contain WD40 repeats, and are implicated in myosin II heavy-chain phosphorylation (MHCK A-D [47]). Another *Dictyostelium* alpha kinase (VwkA) has an upstream von Willebrand factor type A (VWA) domain, and appears orthologous to two Neurospora crassa proteins [48]. ak1 is a unique alpha kinase, and has an ArfGAP domain not found in any other alpha kinases described to date.

PIKKs have catalytic domains related to phosphatidyl inositol 3′4′ kinases, but phosphorylate proteins rather than lipids, and have structural similarity to ePKs [49]. ATM, ATR, TOR, and TRRAP are well conserved in other kinomes, and *Dictyostelium* has orthologs of each of these except the DNA damage response kinase ATM. The lack of ATM correlates with the absence of a homolog of the ATM-binding DNA repair protein NBS1 (G. Manning, unpublished data; [50]). SMG1 is found in *Dictyostelium* and metazoa but not yeast; its presence in *Dictyostelium* may indicate the existence of an RNA surveillance mechanism in primitive organisms. DNAPK is implicated in maintaining chromosomal stability, and had previously been observed only in vertebrates, but *Dictyostelium* has a well-conserved ortholog which has provided the first opportunity to study DNAPK function in an invertebrate model [51].

HisKs use a phosphorelay between aspartate and histidine residues to modulate downstream targets. HisKs autophosphorylate a conserved histidine residue, and then transfer the phosphate to an aspartate residue in the receiver domain, which can be located either on the HisK itself or on a separate polypeptide. In some instances, the phosphate is further relayed to another histidine–aspartate pair on additional polypeptides. HisKs are found in bacteria, fungi, plants, and *Dictyostelium*. Mammals appear to have phosphorylated histidine, but lack detectable HisK homologs (reviewed in [52]).

*Dictyostelium* has 14 putative HisKs (DokA and DhkA-M). All contain a C-terminal receiver domain, and five have transmembrane helices, suggesting a role in extracellular signal transduction. DhkG contains an ePK domain, and has orthologs in cyanobacteria and fungi [53,54]. DokA is involved in osmosensitivity and spore maturation, and DhkA-C are implicated in various aspects of development and spore germination (reviewed in [55]). To date the only known downstream target of HisK signaling in *Dictyostelium* is the cAMP phosphodiesterase RegA. Signaling from HisKs to RegA is mediated by RdeA, which accepts a phosphate group and relays it to the receiver domain on RegA. Five additional proteins have receiver domains (unpublished data), suggesting that there are additional HisK targets.

### Tyrosine Phosphorylation

Tyrosine phosphorylation is well documented in *Dictyostelium,* despite its lack of TK group kinases. The *Dictyostelium* orthologs of STAT and GSK3 are regulated by tyrosine phosphorylation, as in metazoans, and phosphotyrosine western blots show that many additional proteins are phosphorylated, including actin [19,56–59]. *Dictyostelium* also has 12 phosphotyrosine-binding SH2-domain proteins: the Shk1–5 protein kinases in the TKL group, four STATs, an ortholog of the Cbl proto-oncogene, and two others [2]. The SH2 domain of Shk1 is required for localization of the protein to the plasma membrane, suggesting that there is membrane-localized phosphotyrosine in *Dictyostelium* [60].

*Dictyostelium* Mek1 is a member of the dual-specificity STE7 family, and *Dictyostelium* also has three WEE family members, which are predicted to phosphorylate Cdk kinases on tyrosine or tyrosine and threonine residues. In addition, two kinases (SplA and Shk1) have dual-specificity, and four more (Dpyk2–4 and ZakA) have tyrosine kinase activity [18,19,60–62]. Except for ZakA, which phosphorylates GSK3, specific substrates for these kinases are unknown. All six are in *Dictyostelium*-specific families of the TKL group. This finding is consistent with a scenario where TKLs are the ancestors of the TK group, especially since TKLs have sequences that are a hybrid between ser/thr and tyrosine kinases.

Conventional ser/thr protein kinases almost universally have a serine or threonine residue at a position corresponding with Thr201 of PKA; mutation of this residue inactivates PKA [63]. This residue is in the GTPxYxAPE motif, which corresponds to subdomain VIII as described by Hanks and Hunter [64]. TK group members rarely, if ever, have serine or threonine here. Alignments of *Dictyostelium,* yeast, worm, fly, and human protein kinases from outside the TK group, but that are either known to accept tyrosine substrates, or that belong to the WEE and STE7 families, for which the members that have been characterized phosphorylate tyrosine, reveal a great deal of variability in this region. The 12 WEE family kinases all have aspartate here. Ten of the 26 STE7 family members have a threonine at this position; the others have cysteine. Three of the six *Dictyostelium* TKL group members that have been shown to phosphorylate tyrosine have serine or threonine at this position, but the others have cysteine or asparagine. Overall, 61% of non-TK group kinases that accept tyrosine substrates lack serine or threonine at the position corresponding with T201 of PKA. These data suggest that the presence of serine or threonine at position 201 is required for conventional ser/thr protein kinase activity, but optional or disfavored, respectively, for dual-specificity or tyrosine specific activity. We infer from the absence of serine or threonine at this position (Figure S1) that the following kinases from the TKL group may phosphorylate tyrosine: 7TMK1, HH498, DDB0220138, and all members of the ROCO family. In the Other group this criterion suggests that DDB0231179, SAMK-A and B, Vps15, members of the IRE family (except IreA), and members of the Dicty10 family are the most likely to accept tyrosine substrates.

### Receptor Kinases in *Dictyostelium*

**Figure 4:**
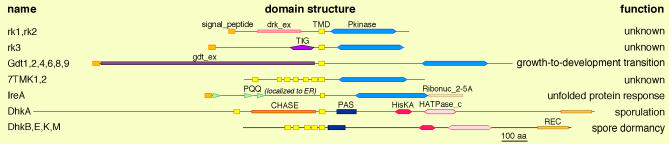
Receptor Kinases The 17 *Dictyostelium* kinases that are strongly predicted to have TMDs are depicted. The drawings are approximately to scale.

A major characteristic of metazoan kinomes is the use of receptor kinases to transduce signals between cells in multicellular organisms. These are found in the TK group (receptor tyrosine kinases) and the TKL group (ser/thr receptor kinases, from the STKR/TGFβ family). The *Dictyostelium* TKL group contains nine kinases with classic receptor kinase domain architectures, consisting of a signal peptide and a single transmembrane domain (TMD) flanking an extracellular region, and a C-terminal intracellular portion containing an ePK domain (Figure 4). Six of these are from the GDT family, and share a highly conserved extracellular domain that presumably detects ligands signaling the onset of starvation [20,21]. The other three receptor kinases in the TKL group are unstudied, and are from the DRK family. rk1 and rk2 have closely related extracellular domains that are unique to *Dictyostelium,* whereas rk3 has an extracellular TIG domain, which is found in receptors from higher eukaryotes, including the MET kinase family.

*Dictyostelium* also has two proteins with kinase domains fused to seven TMDs. This domain architecture has not been observed in other organisms. 7TMK1 is in the TKL group, and is closely related to the nonreceptor, dual-specificity kinase SplA, whereas 7TMK2 has no close homologs. IreA, from the broadly conserved IRE family, has predicted signal peptide and TM domains. Based on studies of orthologs, IreA is likely to localize to the ER membrane and function in the unfolded protein response. Curiously, the IRE family is expanded in *Dictyostelium,* with seven members, compared to one or two in other organisms. All have RNase(L) domains implicated in cleaving a nonclassical intron, but none of the other *Dictyostelium* members appear to be membrane bound. Several other kinases have weakly predicted TMDs, of which the best is SLOB2. SLOB2 contains a predicted N-terminal membrane anchor, and dileucine ER-retention motifs in the C-terminus, and may be secreted to the ER membrane, though all SLOB orthologs appear to be cytoplasmic.

Receptor HisKs are central in an ancient mechanism for the transduction of extracellular signals. In *Dictyostelium* DhkB, E, K, and M have multiple predicted TMDs, and are therefore likely to sense extracellular primary messengers. DhkA has two transmembrane helices flanking a CHASE domain, which is implicated in hormone binding in plant histidine kinases [65].

### MAPK Signaling

MAP kinase phosphorylation cascades consist of MAPKs from the CMGC group, and kinases from the three major families that constitute the STE group: STE7 (MEK, MAP2K), STE11 (MEKK, MAP3K) and STE20 (MEKKK, MAP4K). These pathways typically link extracellular signals and receptors to transcriptional activation of growth and stress-response programs. *Dictyostelium* has single STE7 (Mek1) and STE11 (Mekk) genes, and two MAPKs (Erk1 and Erk2). While *Dictyostelium* has 24 members in the STE20 family, its paucity of MAPK, STE7, and STE11 kinases is striking: yeast and metazoans have six or more MAPKs, and multiple STE7 and STE11 members. Metazoans additionally have a structurally distinct set of MAP3K genes in the TKL group, including RAFs and some MLKs, none of which have clear *Dictyostelium* homologs. Thus *Dictyostelium* offers a simplified system to study MAPK signaling in what may resemble its primordial state.

Like yeast, *Dictyostelium* lacks the stress-responsive Jnk and p38 cascades, having one MAPK in the ubiquitous ERK subfamily (Erk1), and one in the ERK7 subfamily. ERK7s are found in metazoa but not yeast, and the *Dictyostelium* ortholog (Erk2) is involved in oscillatory cAMP signaling during development [66,67]. Its activation is independent of Mek1, which is consistent with findings that the vertebrate Erk7 is regulated by ubiquitination and degradation, rather than phosphorylation [66,68].

### CaM Regulation

CaM-activated kinases occur in plants, yeast, and metazoa, suggesting that this is an ancient regulatory mechanism. Only one *Dictyostelium* kinase, the alpha kinase VwkA, has been shown to be activated by Ca^2+^/CaM, although the binding site has not been mapped [48]. One kinase (DDB0229867, in the LISK family) has an IQ motif, which mediates Ca^2+^-independent CaM binding. This motif is located next to a RhoGEF domain, a pairing that is seen in many metazoan RhoGEFs, indicating that the IQ motif probably regulates the RhoGEF rather than the kinase domain.

Ca^2+^-dependent CaM binding sites are very difficult to predict de novo, so we specifically examined *Dictyostelium* kinases that are orthologs of known CaM-regulated kinases for conservation in the Ca^2+^/CaM-binding sites. We see suggestive conservation of these sites in five *Dictyostelium* CAMK1 kinases—DDB0229351, DDB0216307, DDB0216308, DDB0216312, and pXi—but were not able to detect significant conservation in CaM-binding sites of the CAMKK family.

### Predicted Catalytic Activity of the ePKs

For the human kinome, tentative predictions were made regarding the catalytic activity of ePK domains based on the presence of conserved active site residues [15]. The residues used corresponded to K72 (subdomain II), D166 (subdomain VIB), and D184 (subdomain VII) of cAMP-dependent protein kinase (1ATP.pdb numbering [69]). With alignment uncertainties, and the potential for alternate active site geometries in mind, this approach provides an educated guess as to the catalytic activity of kinases in the absence of biochemical data. By these criteria 29 *Dictyostelium* ePK domains are predicted to be inactive, corresponding to 11% of the kinome, similar to that of humans (Table S2). Structural and mutagenesis studies suggest that conserved residues G52, E91, N171, and D220 (subdomains I, III, VIB, and IX, respectively) also play important catalytic or structural roles [69–71]. There are 22 *Dictyostelium* ePK domains that lack identity at these sites but do have residues corresponding to K72, D166, and D184. Most are missing not only the residue itself, but also the entire conserved motif containing the residue (see sequence alignment in Figure S1, and the “Quality of KD” column in Table S2). The “active” designations of this set of domains are thus qualified, and its members are identified in the “Predicted Activity” column of Table S2.

The lion's share of the *Dictyostelium* inactive ePK domains (22 of 29) occurs in families without yeast or metazoan counterparts, and none are from the AGC, CAMK, or CK1 groups. Those conserved in distantly related organisms include members of the BUB, SCY1, SLOB, and TBCK families. Representatives of the BUB, SCY1, and possibly the SLOB families from other species display catalytic activity in spite of their divergent sequences, while no orthologs from the TBCK family are known to be active [72–75]. A single kinase (roco10) from the expanded ROCO family also appears catalytically inert, while other family members from *Dictyostelium* and metazoa appear active. Important scaffolding and regulatory functions, and kinases with alternate active geometries, are likely to be found among this collection of predicted inactive kinases.

### Dual-Domain Kinases

**Figure 5:**
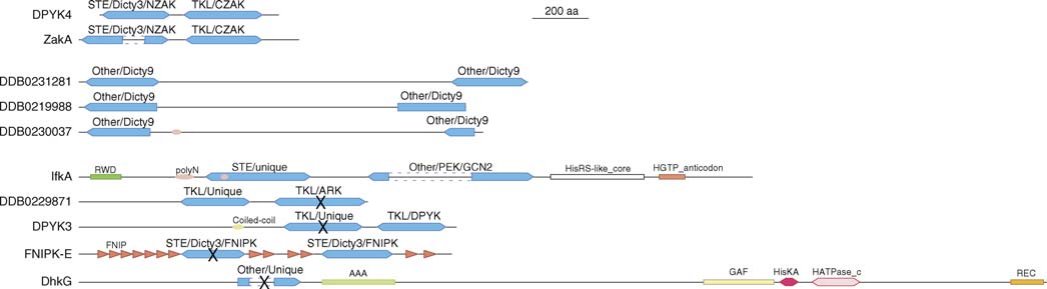
Dual-Domain Kinases The *Dictyostelium* dual-domain kinases are drawn to scale. The kinase domains are shown in blue, broken by a dashed line in instances where there is a large insert in the kinase domains. The classification (group/family/subfamily) of each kinase domain is indicated above each domain. An X through the kinase domain indicates that it is predicted to be catalytically inactive.

*Dictyostelium* has nine proteins with two ePK domains and one with an ePK and a HisK domain—proportionally more than the 13 dual-domain kinases found in humans (Figure 5). The human kinases are in the Jak, RSK, Trio, and GCN2 families, of which only GCN2 (IfkA) is found in *Dictyostelium*. IfkA differs from its yeast and metazoan counterparts in that its N-terminal domain is predicted to be active, suggesting possible differences in how *Dictyostelium* and the later-diverging eukaryotes respond to amino acid starvation. The combination of active and inactive domains, as seen in other GCN2 homologs and Jaks, recurs in four apparently unrelated dual-domain kinases in *Dictyostelium*. In all but DDB0229871 the N-terminus domain is predicted to be inactive, and in DhkG an inactive ePK domain occurs with a HisK domain. This variability indicates that kinases combining active and inactive domains have evolved on multiple occasions.

Except for IfkA and DhkG, all the dual-domain ePKs are from *Dictyostelium*-specific families from the STE, TKL, and Other groups. ZakA, DPYK3, and DPYK4, which all have one or both domains from the TKL group, have tyrosine kinase activity [19,62]. The *Dictyostelium* dual-domain kinases appear to have arisen by two distinct mechanisms: by tandem duplication of the same class of kinase domain, as seen in the Other/Dicty9 family, or by fusion of two distinct kinase domains, such as the STE and TKL kinase domains of ZakA and DPYK4.

### Protein Kinase Pseudogenes

**Figure 6:**
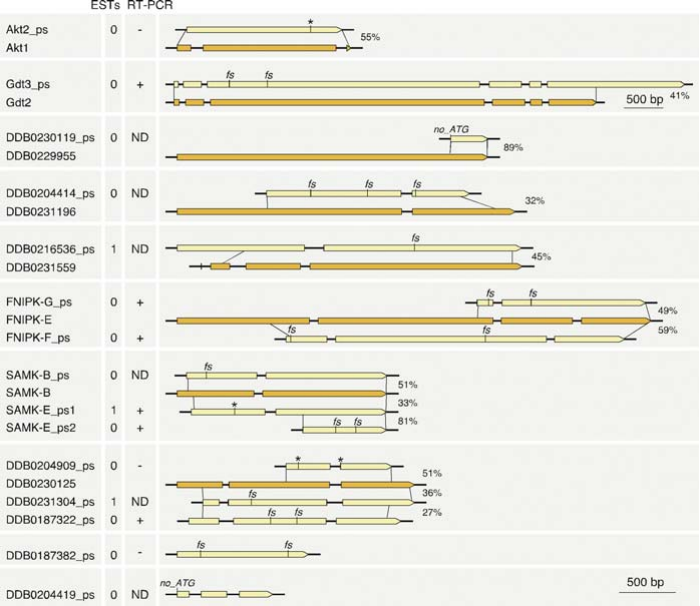
Protein Kinase Pseudogenes The genomic loci for putative pseudogenes, together with their most likely parental genes, are shown. Pseudogene exons are shaded yellow, and parental gene exons are shaded orange. Stop codons are indicated with an asterisk, and frameshifts by “fs.” The percentage identity in amino acid sequence between pairs of sequences is shown to the right of the drawings. The number of ESTs, as reported at http://www.dictybase.org, is shown. The results of RT-PCR experiments are shown. +, a product was obtained; −, no product was obtained; ND, not determined. All are drawn to the scale shown in the bottom right of the figure, except Gdt3_ps, which has a condensed scale. For DDB0187382_ps and DDB0204419_ps, no putative parental gene could be identified.

*Dictyostelium* pseudogenes are not well documented, with only two reported to date [21,76]. We identified 15 likely ePK pseudogenes, based on the presence of frameshifts and/or nonsense codons within otherwise conserved regions (Figure 6). These loci had all been annotated as genes by automated gene prediction programs, with the disabling mutations masked, either by introducing introns, or by truncating the ORF. Three have a single EST, and six more gave positive RT-PCR reactions, indicating a residual level of gene transcription. In these cases, RT-PCR and EST data were used to disprove introns that had been introduced by the automated gene prediction algorithms.

Putative parental genes were identified for 13 of 15 pseudogenes. In general, pseudogenes share intron location with their parent, suggesting that they arose by gene duplication followed by inactivation of one of the copies, rather than by reverse transcription. The clearest exception is *akt2_ps,* which has no introns, whereas *akt1* has two. Three pseudogenes lack one intron but conserve one or two others, compared with their parents, and may reflect retrotransposition of immature transcripts. The degree of similarity between the pseudogenes and their proposed donor gene varies from 27%–89% amino acid identity, and often localized regions with >90% identity are found. As in other species, the family distribution of pseudogenes is highly skewed, with eight pseudogenes arising from just three parents, and all but *akt2_ps* arising from families that are unique to *Dictyostelium*.

### Domain Architecture of *Dictyostelium* Kinases

The majority of *Dictyostelium* kinases contain additional motifs and domains that act to localize, modulate or interact with the kinase domain. Figure S3 shows the extensive variety of domain architectures observed. Predicted domains, TMDs, and signal peptides are illustrated, as well as polyN and polyQ tracts, which are very common in *Dictyostelium* proteins, although their function is not known [2]. Notes on each domain are given in the legend to Figure S3. Other signaling domains are frequently seen in the *Dictyostelium* kinases. Several small G-protein GAPs and GEFs occur: a LISK and a ROCO have RasGEFs, another LISK and a unique kinase in the STE group have RhoGEF domains, two ROCO family members and another unique kinase in the STE group have RhoGAPs, and an alpha kinase has an ArfGAP domain. WD40 repeats were found in 14 kinases from the STE11, CMGC-unique, CZAK, VPS15, Other-Unique, and Alpha families, suggesting that these proteins are involved in myosin II regulation and/or heterotrimeric G-protein signaling. An association with heterotrimeric G-protein signaling is also suggested for Rck1 (in the TKL/Dicty5 subfamily) and ARCK-1 (in the TKL/ARK family), based on the presence of RGS domains in these polypeptides. One ROCO has a myotubularin (lipid phosphatase) domain, and a novel kinase has a dual-specificity protein phosphatase domain. In general, these domains are either not found on orthologs of the kinases from metazoa, or they are on kinases from *Dictyostelium*-specific classes.

### Kinases and Human Disease

More than 150 human kinases are currently known to be associated with disease [77], and of these, 30 are in 22 subfamilies found in *Dictyostelium* (Table 3). Most of these subfamilies are found not only in *Dictyostelium,* but are also conserved in yeast, flies, and worms, suggesting that they play important roles in fundamental cellular processes, and that studies in a variety of model organisms will be useful in elucidating roles and developing therapies. However, yeast lacks five of these subfamilies (LKB1, DYRK1, ROCO, PEK, and BRD), one is missing from yeast and flies (SGK), and DNAPK is found only in *Dictyostelium* and vertebrates. In these instances, *Dictyostelium* is uniquely situated as a tractable unicellular model organism. The ability to quickly generate gene disruptions in *Dictyostelium* makes it particularly useful for understanding the cellular functions of disease-associated kinases. In addition, *Dictyostelium* may prove useful for kinase drug screens, and for understanding resistance to and side effects of drugs [78,79].

**Table 3:**
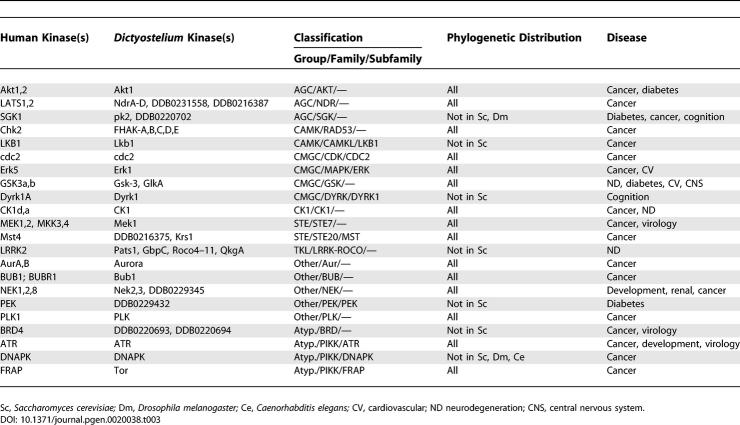
Human Kinases Implicated in Disease That Are Conserved in *Dictyostelium*

### Conclusions

The comprehensive catalog of *Dictyostelium* kinases presented here provides insights into the early evolution of protein kinases, and a resource for future signaling research in this organism. Since kinases frequently act in concert, and are key modulators of most cellular pathways, experimental whole-kinome approaches may be fruitful in dissecting *Dictyostelium* signaling and cell biology. In other kinomes, large scale knockouts, RNAi, and protein chips have been employed successfully, and such technologies may now also be applied to *Dictyostelium* [80–84]. These may be integrated with global protein interaction maps and expression profiles, and correlated with the emerging data from other species to understand both conserved and *Dictyostelium*-specific variants in kinase signaling pathways.

While conservation of function is a major theme, this study also indicates plasticity and variation. For instance, metazoan BRSK and CDK5 kinases function predominantly in neurite outgrowth and neurotransmitter release in the brain, yet these functions must have evolved from a non-neuronal one in their common ancestor with *Dictyostelium*. There are also cases of possible functional displacement. For example, the myosin light-chain kinase activity in *Dictyostelium* is carried out by a related CAMK1 kinase (MLCK-A), and signaling from lipids and small GTPases to myosin is mediated through the PH and CRIB domains of DMPK kinases in metazoans, but the PH and CRIB domains of a PAK kinase in *Dictyostelium*. The unique absence of PKC isozymes in *Dictyostelium,* coupled with the *Dictyostelium*-specific presence of C1 domains on other kinases is also an intriguing structural shift that may indicate displacement of function.

Our understanding of the structure and evolution of *Dictyostelium* kinases will be enhanced by additional genome sequences. Preliminary evidence (J. L. Smith, unpublished data) indicates that several *Dictyostelium*-specific families have close homologs in the *Entamoeba* genome, providing a resource to understand their evolution, and possible models to develop drugs against such apicomplexan parasites. The sequencing of *Dictyostelium* sibling species will also greatly aid in modeling gene structure and looking at close-range gene evolution.

## Materials and Methods

### Discovery of *Dictyostelium* protein kinase genes.

Sequence databases consisted of version 2 of the *Dictyostelium* genome and its ORF predictions (http://www.dictybase.org), and *Dictyostelium* EST sequences from GenBank. Kinase genes were detected using HMMs built on known ePKs. For the final catalog, an in-house HMM with a cut-off of e = 0.04 was used. One sequence, encoding polyA ribonuclease (DDB0218147), was removed from this catalog; polyA ribonucleases have residual sequence homology to ePKs, sometimes leading to their misannotation. The *Dictyostelium* Bub1 ortholog was not found in these HMM searches, but was recovered using BLAST searches. For the various aPK families, in-house HMMs were used, as well as BLAST queries. All genomic and EST hits overlapped with predicted proteins from the genome annotation effort.

All automated gene predictions were inspected, and 20% were edited to give a more plausible gene model. Most of these findings were incorporated into the curated models presented at dictyBase. In instances where frameshifts or stop codons prevented formation of the best gene model, individual reads from the genome sequencing project (http://dicty.sdsc.edu) and EST sequences were examined to determine whether these were errors in the genome sequence. The sequences of four genes *(pXi, PakC, DNAPK,* and *TRRAP)* were corrected using this approach. We could not find a plausible start codon for SplA, and as a result, it has incomplete protein sequence information. Nek4 and pk4 abut gaps in DNA sequence data, and as a result our current protein sequence version for these is truncated. Two other genes *(DDB0218878* and *DDB0219793)* span gaps in the genomic sequence data, and the halves were manually joined. For Gdt9, in-house sequencing was used to correct an ambiguous region, resulting in an improved gene model.

The quality of each ePK domain was evaluated based on the presence of known protein kinase subdomains [64] and kinase HMM scores. The results of this evaluation are given in the “Quality of KD” and “Notes” columns of Table S2, and details of the evaluation are given in the table footnote.

### Identification of pseudogenes.

The initial collection of ePK gene predictions was screened for pseudogenes as follows. Several were identified because the predicted protein had a deletion in an otherwise well-conserved region; upon inspection, the deletion was found to correspond to a bogus intron that encoded the missing homologous sequence, but interrupted by a stop codon or frameshift. Similarly, we inspected kinases that were expected to have additional conserved sequences upstream or downstream of the gene prediction; in some instances the conserved sequences were found, separated from the main ORF by stop codons or frameshifts. We also evaluated all of the introns in the gene models for plausibility, and flagged those that had an intron that appeared coding, rather than intronic (typically, *Dictyostelium* introns are <15% GC, and 60–250 nt in size). We also screened translated genomic DNA for ePK-like sequences in order to identify more cryptic pseudogenes, but all hits had already been predicted into genes.

### Identification of additional domains in kinases.

Sequences were analyzed for the presence of additional domains using InterPro [85]. Additional domains were detected using profiles derived from publications (cor and roc domains [38]) or generated in-house (PH domains found in Pdk1 enzymes, extracellular gdt domain, extracellular drk domain, and mob-binding domain). Potential coiled-coil regions were predicted using paircoil ([86]; http://paircoil.lcs.mit.edu). Transmembrane helices were predicted using TMHMM [87], and signal peptides were predicted using the Sigcleave module at the SMART website (http://smart.embl-heidelberg.de). Regions of poly-asparagine or poly-glutamine were detected using Prosite [88], modified to return segments of at least 19 residues containing 90% or more N/Q.

### Classification.

*Dictyostelium* kinases were mapped to the classification of Manning et al. ([15]; http://kinase.com). Criteria included BLAST scores to previously classified kinases, using both kinase domains and full-length proteins, family- and subfamily-specific HMMs, relationship trees built from kinase domains from *Dictyostelium* and other species, and inspection of the proteins for features outside of the kinase domain that are characteristic of a particular family or subfamily. Where necessary, new families and subfamilies were created.

### Relationship tree.

The alignments underlying the tree were made using MUSCLE [89] and hmmalign [90], and were optimized by hand. Flanking sequences and internal insertions were removed by excluding residues not matching the HMMs. Group-specific HMMs based on yeast, metazoan and *Dictyostelium* ePK sequences were used to align ePK domains from the *Dictyostelium* AGC, CAMK, CMGC, STE, and TKL groups, and to generate group-representative sequences consisting of the top-scoring residues for each position. A *Dictyostelium*-specific ePK HMM was used to align members of the Other and CK1 groups, together with the representative sequences for the AGC, CAMK, CMGC, STE, and TKL groups.

Consensus trees based on 100 bootstrap samples from each alignment were made using the neighbor-joining method of Phylip v3.63 [91]. The group-specific trees were grafted onto the Other/CK1 tree at the sites held by their representative sequences. Branch lengths were reintroduced to the combined consensus tree using ProML, a maximum-likelihood algorithm, with a *Dictyostelium* HMM alignment as input.

## Supporting Information

Dataset S1 The Tree in PHYLIP Format “DDB0” is substituted with “k” to satisfy format constraints. While the trees given here and in Figure 1 are topologically identical, they differ by topologically neutral rotations about nodes in few regions.
(5 KB TXT)

Figure S1 Sequence Alignment of *Dictyostelium* ePK Domains An alignment of all *Dictyostelium* ePK kinase domains (except pseudogenes and Chromosome 2 duplicates) is shown. For proteins with two kinase domains, the domains are distinguished with an “a” or “b” suffix. The amino acid position of the first residue in the kinase domain is given. For deletions within the alignment, the number of residues that were removed is indicated in parentheses. The kinases names are shaded by group: yellow, AGC; purple, CAMK; pink, CMGC; blue, STE; green, TKL; orange, CK1; and gray, OTHER. The alignment is shaded to depict regions of similarity on a group-by-group basis. Some kinases from the Other group were shaded with the group they are most closely related to. Subdomain designations correspond to those used in [64].
(864 KB PDF)

Figure S2 *Dictyostelium* Text Tree The tree shown in Figure 1 is shown here in text format with bootstrap values indicated at the nodes. Nodes at which group specific trees were grafted to the main tree are designated by the word “fixed.”
(140 KB PDF)

Figure S3 Domain Drawing of the *Dictyostelium* Kinases Matches to PFAM, SMART, and in-house HMMs, polyN and polyQ stretches (common in *Dictyostelium* proteins), transmembrane helices (TMDs), and signal peptides are shown. Motifs are labeled the first time they appear in each group, and the first time they appear on each page. All proteins are drawn to scale, and the vertical lines represent 100 aa intervals. A brief description of each of the motifs is given in the legend.
(210 KB PDF)

Table S1 Group, Family, and Subfamily Abbreviations (33 KB XLS)

Table S2 Summary of *Dictyostelium* Protein Kinases The protein sequences used in the analyses for this paper are presented. In most instances they correspond to a curated model at dictyBase, and their accession number (DDB#) is given. In cases where our version differs from the current dictyBase model, the relevant DDB# is italicized and appended with a one-letter code. A “p” indicates a pseudogene; these proteins contain internal asterisks to indicate stop codons, and Xs to indicate frameshifts. An “e” indicates an edited gene model (i.e*.*, our interpretation of the genomic data differs from the model presented at dictyBase). A “c” indicates a model that is based on corrected genomic DNA; these corrections were made based on inspection of EST sequence data, genomic reads, or our own sequence data.
A rating for each ePK domain is given in the “Quality of KD” column. The rating reflects the degree of similarity to a canonical ePK, and is largely based on conservation around and including the following motifs: gxGxxg in subdomain I; vaiK in subdomain II; rEi in subdomain III; HRDxxxxN in subdomain VI; DFG in subdomain VII; and Diws in subdomain IX (subdomain nomenclature of [64]). Sequences containing all of these motifs are rated “kd” (kinase domain); sequences lacking from one or two are rated “partial_kd,” while sequences with at least one clearly recognizable kinase motif but lacking three or more others were designated “kmc” (kinase motif-containing).
In a few cases sequences failing to match three conserved motifs were admitted as partial_kds because of good alignment elsewhere or a good kinase HMM score. Specific rationales for the ratings are given in the “Notes” column. Of the 255 nonpseudogene ePK domains in *Dictyostelium* (nine proteins have dual ePK domains), 210 are designated “kd,” 37 as “partial_kd,” and three as “kmc.” Five sequences were given special designations, because they are in the BUB, SCY, or SLOB families, which diverge strongly from the ePK consensus, but are well conserved across species.
In the “Predicted Activity” column, “a” (active) and “i” (inactive) refer to the catalytic activity predicted as described in the text. The portions of the alignment used to make these predictions (the VAIK, HRD, and DFG motifs) are shown. If the entry is blank, the ePK domain starts (or ends) after (or before) that motif. If the entry contains only periods, the ePK flanks the domain, but does not match that particular motif. In several cases the activity prediction is qualified because of the lack of conserved residues G52, E91, N171, or D220, as discussed in the text. In these cases “q” is appended to the activity flag and the qualification is described in the “Notes” column. For dual-domain kinases the properties of the individual ePK domains are separated by a slash.
(401 KB XLS)

Table S3 Species Distribution of Protein Kinase Families and Subfamilies The number of kinases in each group, family, and subfamily in *Dictyostelium,* yeast, flies, worms, and humans are summarized. The current classification from http://kinase.com is used. Pseudogenes and copies found on the Chromosome 2 duplication are not counted in the *Dictyostelium* numbers. Unique kinases in each group are not related to kinases from other organisms, and are therefore tabulated under Sections G, H, and I.
(57 KB XLS)

## Abbreviations

aPK: atypical protein kinase
CaM: calmodulin
CK1: cell or casein kinase 1
ePK: eukaryotic protein kinase
EST: expressed sequence tag
HisK: histidine kinase
HMM: hidden Markov model
TKL: tyrosine kinase–like
TMD: transmembrane domain
