# Supplementary material for: The Dictyostelium Kinome—Analysis of the Protein Kinases from a Simple Model Organism
Source: PLoS Genet. 2006 Mar 31;2(3):e38. doi: 10.1371/journal.pgen.0020038 (PMC1420674; doi:10.1371/journal.pgen.0020038)
Supplement: Figure S1 — An alignment of all Dictyostelium ePK kinase domains (except pseudogenes and Chromosome 2 duplicates) is shown. For proteins with two kinase domains, the domains are distinguished with an “a” or “b” suffix. The amino acid position of the first residue in the kinase domain is given. For deletions within the alignment, the number of residues that were removed is indicated in parentheses. The kinases names are shaded by group: yellow, AGC; purple, CAMK; pink, CMGC; blue, STE; green, TKL; orange, CK1; and gray, OTHER. The alignment is shaded to depict regions of similarity on a group-by-group basis. Some kinases from the Other group were shaded with the group they are most closely related to. Subdomain designations correspond to those used in [64]. (864 KB PDF) [file pgen.0020038.sg001.pdf]

```
                    Subdomain I                    Subdomain II                               Subdomain III           Subdomain IV
Akt1          120 FELLNLVGKGSFGKV.IQVRKKDTGE........VYAMKVLSKK.........................HIVEHNEVEHTLSERNILQKIN.........HPFLVNLNYSFQ
DDB0220670    173 FIFLRMIGRGAFGQV.NLVKKIDTGR........LYAMKVIKKD.........................KCLNMNAVGHTFTERKLLKKYY.........HPFIVGLKYSFQ
DDB0216386     86 YYFIKPISKGGYGQV.FLAITKSN.K........FFAIKSINKR.........................DTIGKHMVKELMVERNILVQYQ.........NQSIVKLFECFQ
DDB0220701   1527 FEIIKPISRGAFGRV.YLAQKKKTGD........LYAIKVLKKL.........................DTIRKNMVNHVIVERNILAMVQ.........NEFVVKLFYAFQ
DDB0220700     64 FNFLKVISKGGFGKV.YLAENKITKK........TVAIKVIKKS.........................DTIKKMMVDQVNIEKTILSNYK.........CNFIVELFSSFQ
DDB0185113    810 FEFIKPITKGGYGKV.FLAKKIRTGD........IYAIKRLKKS.........................DMIKKNQLDHVKVERNILAYTS.........NPFVVKMYYSFQ
DDB0229452    232 YKHVECIGKGGYGVV.CKFINKKTNE........IRAIKTIRKD.................................YSALKEINTLKELG.........GQFTVNIFHTFL
NdrA          112 FEVIRIIGRGAFGEV.SLVRHRESND........LYAMKRLKKS.........................EMLKKEQAAHVRAERDVLASAN.........TNWVVKLYYSFQ
NdrB          130 FESIRIIGRGAFGEV.RLVKMKKNNK........FFAMKKLDKS.........................KMIEKHQTIHVRSERDILADSNNI....HGSNPWIVSLYYSFQ
NdrC          718 FKILTQIGKGGFGQV.FLAQKKDTGD........IVTLKRLKKQ.........................TVEWANQRNQVSQEKSVML.VD.........NKWITKLLYSFQ
NdrD         1555 FQKLTAIGKGGFGKV.YLARKKDSNE........IVTLKVIRKS.........................TYHRANQMTSVTKEKEVMMIPHSSK....DQSQWITRLLYSFH
DDB0231558    756 FTLIEKIGEGGFGQV.FLAKKKDTGE........IVAIKRMSKE.........................LIWSKNKVSHIKNERDILAQGKN........HRWIVSLVYSFQ
DDB0216387     40 YDIISTIGSGSYGEV.CIVREKSTKQ........VYAMKKIYYT........................EVSDTELVKKRALRERDAMVICNNKN.....NKRAPKLYCSFID
Pdk1A          69 FIIGKVLGEGSYGAV.VLGTEKETQQ........QYAIKILEKK.........................QIIKENKIKYVQIEKEIFCKSN.........HPNIVKLFFTFR
Pdk1B         271 FDFIRTIGKGAYGKV.KLVIEKETQL........IFASKILNKK.........................LIIKEKKAKYVNTEKTILDSLD.........NPNIVKLFYTFQ
PKA           336 FKQIRVIGTGTFGKV.YLIQNTKDGC........YYAMKCLNKA.........................YVVQLKQVEHLNSEKSILSSIH.........HPFIVNLYQAFQ
pk4           679 FKLFDLLGSGSFAKV.RLCQHIPSER........LFCMKILNQN.........................KIIRLRQEVHVCNEKQVLMLTD.........NPFIVKLYSTFK
DDB0185224    334 FNFYGSLGSGSFGTA.KLCRHRGSGL........FFCSKTLRRE.........................TIVHEKHKEHVNNEINIMLNIS.........HPYIVKTYSTFN
pk2           153 FELLNVIGKGSFGKV.MQVKKKGEDK........IFAMKVLRKD.........................AIIARKQVNHTKSEKTILQCIS.........HPFIVNLHYAFQ
DDB0220702    128 FIIKHLVGKGGFGKV.FQVVHVDTQK........VYALKVIKKN.........................HIIAKKSVVNTLAEKDILKKIS.........HPFIVNLHYAFQ
pk3           498 FELLKVLGVGSFGRV.YLVRRKDTGK........FYAMKVLNKK.........................DMLKKKQIAHTNTEKMVLSTMD.........HPFIVRLHFAFQ
Aurora        110 FDIGKLLGMGRFGHV.YLAREKKSQF........IVALKVLFKN.........................QLQTHNIEHQLRREIEIQSHLR.........HPNILRLFGYFY

MLCK-A          8 YEFKEELGRGAFSIV.YLGENKQTKQ........RYAIKVINKS..........................ELGKDYEKNLKMEVDILKKVN.........HPNIIALKELFD
DDB0216385     13 YNITDIIGEGTFSTV.TLANHIEKIE.......DKYAIKIISKE..........................VLDNERRTYVDWEISILSKCQ.........HPNIIKFYEHYE
DDB0216309     38 YILGNEIGRGAFSIV.REATSRATGT........KVAIKSINTR.............................FIKNKLLMREIEIMKKVGD........HPNILKLYEVYE
pXi            18 YEIGSQIGNGKFAQV.HSGTNKSTGK........KSAIKIMKKS.............................IVEESAIIKEIEMMTEIN.........HQNIIKLQEVYE
DDB0216308     20 YEFGPEIGRGAFSIV.RQGTHKDTGD........QVAIKAISKQ..........................HVSEADMKRFTREIEIMKKLK.........HKNIIQLIEVFD
DDB0229351    566 YQIFDRLGSGTFSDV.YLCINKENGK........QYAMKIIDKS.......................LVTMIAQHTDMKVETEVNILKQSF.........HPHVIQIIDHFE
DDB0216307     56 YVVGKELGRGAFSVV.REGTRKTTSD........KVALKYIEKK..........................FVKKKHIEQLRREIDIMKKVK.........HENVLSLKEIFE
DDB0216312      6 YELHKEIGKGAFSVV.FLVTEKKTKK........QWAMKIIDKK.............................SSSKAALETEIEIMKKVD.........HPNIVKMHEYFE
Snf1           31 YRLDKTLGIGSFGKV.KLAEHIRTGV........KVAIKILNRT.........................KIKNLKMDEKIRREIQNMKLFR.........HPHIIKLYEVIE
DDB0229364      9 FIIGKTLGQGTTGKV.KLGFHKDTGF........KVGIKIINKE........................LLINKPSMRRKIEREIVLMKLID.........HPNAMKMYEVYE
Lkb1           40 YILGEVLGEGAYGKV.KDGMDSFTQK........RVAVKILKRA........................RLKKIPGGEASVLKEINITKKLH.........NKHIIKLIDHFI
MARK-A        109 YLVIKTIGRGQFGKV.KLGYHKKIPN.......EKVAIKIINKG..........................KLDPETLKMVQREVRIMKLLH.........HPNIIRLYEVIE
MARK-B         65 YLLGKTIGSGTSSKV.KIGTNILTGK........QYAIKITKPK............................RIKERKEIEREISILKRLK.........HDNIIQLHDAIY
MARK-C         46 YEVGKTLGNGTFGKV.KLGTNICTKE........NVAIKFIKNN..........................KLSGKQKETCFREIDIMKLLD.........HPNIVKLLDVVD
DDB0231454    542 YTLGKTLGRGNYGVV.KLGTHINTKE........EIAIKILYKE..........................QMTESEFTRCKREIEILKQLY.........HPFINKLLNVLE
DDB0219986   1158 YDIIKTLSTHPHN.V.YLANFNDQ..........LVVLKEFGIG............................DAFGKQIFERQVSLLKQMN.........HKCIMPIQAVFY
FHAK-A        180 YDFIKELGSGNFSVV.YEGVNKNTGK........RVAIKHLNLS.......................KINTHTPKFKSQLNREIEILKFIN.........HENVVEIYDIFY
FHAK-B        625 YYFVKEIGSGGYGIV.YEGLYKLNGK........RVAIKHIDLL........................KNGSTSKSMELVSKEYNALKNIK.........HRNVIEFFDIVF
FHAK-C        199 YSIQGILGTGNFSVV.KRCIRRDTGE........VFAVKIIDKK........................KFWSQTKTRRQMESEVEILQKIK.........HPNIISIIDIVQ
FHAK-D        218 YDLREVLGTGNFASV.RLGVEKETGN........KYAIKIIDKK........................KMSMTSKRKDSLMDEVNVLTKVK.........HQNIISIKEVFE
FHAK-E        145 YFIGEMLGQGNFATV.KLAVERTTGV........KYAVKIVDKK.......................KYFMNSSARKDSLMDEVNILRGLS.........HPNIIQIIEVFE
DDB0220010    201 YVFVRKLGKGTFGKV.KLAYHHDTHH........LYAIKIFNKI..................RLKKQTMGIGRPNAFDDVLKEIAIMKKMN.........HINVVKLYEVIN
Apg1            7 YILDKRIGWGAFAQV.YKGFSIKTNE........PFAIKVVDVC.......................RLADKNSKLTENLNYEIRILKELS.........HTNIVRLYDVLN
tsunami         5 YKLIGQIGEGSFGKV.YKYRKKFTGQ........LVACKVISKK..........................GKNEEDILSLRQEIDILKNLS.........HPNIIQFISCFE

Cdk1           10 YQKLEKLGEGTYGKV.YKAKEKATGR........MVALKKIRLE............................DDGVPSTALREISLLKEVP.........HPNVVSLFDVLH
Cdk5            4 YSKIEKLGEGTYGIV.YKAKNRETGE........IVALKRIRLD..........................SEDEGVPCTAIREISLLKELK.........HPNIVRLHDVIH
Cdk7            4 YNIEALIGEGTYGVV.SRATVKATGQ........IVAIKKIRKI......................LIQNQTDDGINFSAIREIKILQELK.........HDNVVNLLDIFA
Cdk8           44 YTFSYEIGSGTYGMV.YKADDKKRPN.......NKVAVKKFRST..........................KEGEGLSLTAYREIGLLKELS.........NENIVKLLDVCL
Cdk9          209 HEKIEQIGEGTFGQV.YKAKNKSNGD........IVALKKVIMD..........................NEVEGFPITAIREIKILKELN.........HANVVNLKEVVT
Cdk10           7 FEKLDSIGEGTYGIV.SKGRDKETGR........IVALKKVKIG.........................QQDKDGIPLTSLREIQILKEIK.........HPNIVSLLEVVI
Cdk11          52 FKKLYTINEGAFGVV.YCAQDKETEE........IVALKKIKME..........................REREGIPITSVREIKVLMELK.........HDNIVQIKEIVL
DDB0229294     64 YEIISKIGEGISGSV.FKAIKKGTEE........MVALKNFKGW.............................TEGDRASKEECSLLQQLR.........HIPYITPVIDIY
DDB0229424      4 FQIIELIGSGSYGKV.YKAIHNLSKC........TVALKIISVM..............................NIENGLPVEVKYLMKLRD........CKNIVHLIEYFY
DDB0230050      4 YIILSKCGQGTYGSV.FKGIHKITHS........LVALKRVTDI..............................AQEDGEPVEVKYLNQLKN........LSNIVNLRDHFY
CK2            47 YEIIRKIGRGKYSEV.FEGANIKNNE........KCVIKVLKPV...............................KKKKIKREIKILQNLCG........GPNIITLYDVVR
DDB0219953    312 YEVIQKIGRGKYSEV.FSGIDIETSD........EVVIKVLKPV...............................QKLKIQREIKILESLNG........GPNIIPLLDSVK
ClkA          590 YKVLCTVGSGTFSTV.VECWDTNSSG........QVAIKIVRSA.............................KKYTEDALVEIDILRNLEKTGNSNGKYLSHCIRLLDSFL
DDB0229841     74 IDPNTIVDCGTNGIM.FIAFNFETQK........RVILKKLSKR.........................MFDNELNGHRIIRNLIFQNLFQG........GKHISTYQSIFK
DDB0229333     46 ITKKTIYACDINGTM.FQTYNMDTNR........LLIIKKISLL.........................QFKNHLQGRMMLRNCHYQRFFNH........HPFVSGFKVSKR
DDB0229296    102 INLKSITDCGPDGVM.FRAKNEDSKE........EVIVKKISVF.........................LMKDDKMARKLLRNLLFQRHFQQ........HPLVSTFQSVFK
Dyrk1         304 FEIISSLGKGSFGQV.VKAFDSVLKE........YVAIKIIKNK.............................VPFYNQALIEIRLLELMNNKD...PEDQYKIIKLKHHFK
Dyrk2         605 FEIVSILGQGSFCQV.VKGYDYKTGE........MVALKILRNQ.............................KRFHNQALTEIKILEYLKTND...PNSTASIVHLNNYFY
```

```
                              Subdomain I                          Subdomain II                                                 Subdomain III                     Subdomain IV
YakA            205 YKVLDSLGQGTFGQV.VKCKNCDTDE.........LVAIKILKNK.............................QAYFQQGRLEIQTLKSLNDQHD..PEDKNHILRLLDSFI
Prpf4B          490 YQIFSPIGSGVFSTV.VSAKETKTNE.........DVVIKIIRNR.............................PSMHRSGLKEIEILQKISNTPTS.SNQKSHCIQMKDHFN
GSK-3            56 YITEGVIGNGSFGVV.TQAIVADTKE.........VVAIKKVLQD...................................QRYKNRELQIMKMLN.........HINIVSLKNSFY
GlkA             91 YEIIKQVGQGTFGKV.YEAKNQDNKR..........VAIKKVEKS..................................NHFISREYDILKIVA.........HPNCLRILDMFY
Erk1            149 YSIVKCIGHGAYGVV.CSAKDNLTGE.........KVAIKKISKA.........................FDNLKDTKRTLREIHLLRHFK.........HENLISIKDILK
Erk2             14 YEVLQKIGKGAYGIV.WKAIDKKTKQ.........TVALKKIFDA.........................FQNATDAQRTFREIMFLQELHG.........HENIIKLLNVIK
DDB0229430        4 YQFIKQVGDGAYGDV.IKAIDVKTGE.........IVAIKRMKKK..........................FSDWKECIQLREIKALKKLK.........HPNIVKLLEIIL
Sky1            135 YQVVDKLGWGHFSTV.WLCNDKDTPITTSS(29)QVALKIVRSA.............................RTYSETAEDEIKILNAISKY...NAQDKCVARLLDHFT
DDB0230051      414 QIHKVSIGNGCGHFV.IKLIPTVSGTNVG.....NSYSKFIYNS.....................IYSSTLAESEILLFREAMLLYKLN.........HNNILKLESITK
DDB0230126      654 YHLGPLISE.NSTTV.FRAFDCKSLI.........PVAVKVLPPV.....................................ALNIPV.........HENLTKVYQVCQ
Cdc7            622 YRILEKIGQGTFSGV.YKSVCIDGPNI......GLIVALKRVAPT...........................SSPARILNEIHSLLRVGG.........HYNVSALFGALR

DDB0229971       77 VISDIAIGKGAFATV.FKGLNTLSGD.........FVAIKRFEKS.........................KISNEQHSSVSTEFDILQRLN.........HENIVRILGREE
DDB0229896       18 YNLGVVIGKGGFGTV.YQGLDIEDGD.........FVAIKQINLT.........................KIPKDQLQGIMNEIDLLKNLN.........HANIVKYIKYVK
DDB0229877       13 YQIGEAVGKGAFGKV.FKALNAETGD.........FCAIKQIEKT.........................IISEKQLPSIIQEIKLLQTLQ.........HPNIVKFIESYE
DDB0231212       22 YLLIDIIGKGGFGVV.YKGLHKTKGY.........FSAIKKIKIM.....................KKKKLQSESQISLMAEINLLKVLS.........HHNIVRYYEHIP
DDB0229915       36 YTLGEKIGRGAFGQV.FKGLNGKTGE.........FVAIKQIDSN.........................KIDESSLQSVKGEVEILHKLR.........HNNIVKVLGVVE
FNIPK-A          11 WEILSQLGTGAFGRV.VKAKKINCDGTGI..IIDICAIKIIKKS...................................VFTKNEIEILKQLD.........HPLIVKYYGYGV
FNIPK-B          11 WEIVETL....KSNV.FKVNNKNNIVLNG.NSFKTCILKIIINK........................................DKNVWKEGQVLEKLKN.........IDSIVKCYGWCC
FNIPK-C          33 FEIIRILKKDEFSTT.CKAKKLSGQFNEK..RVNFCIIKIIKKR...........VKKFDSKKNEIIIEIDPRKEREILEKIK.........HICVLEYYGYSE
FNIPK-D          33 WEIITQLESNEQFTV.YMSKKLKGPNFTN.NRFGLCTVKKIKSQ.................................YFNENEILNHIKLRD......NKYVLKYYGCGK
FNIPK-Ea        368 WEIIKP..........QKNISEN.............KVYYT...............................TKHKNAKIIDVLN.........ICKCSLKLYGYVK
FNIPK-Eb        860 WEIISTLGSGNFGKV.FKARKINGIINGS..KVSLCAIKKIEKK.........................DKLKIKLSTEVEILNKLKD.........NEHSMKYYGYGY
DPYK4-a           9 WEEISSIGSCNSKSRVLKCRKKNGLIENE..KVDIVAVKIINKK.................................FFKRNETDILEKIR.........LFNIPRYYSHAE
ZakA-a            9 WEEISFIGEGQYGRV.IKCRKKNGFILNE..QVDYVAIKIISKD.................................KFKRNETDILEKIR.........LFNIPRYYSHAE
DDB0230002       16 IEKNYR..KGGFSKI.SKAKKKNGIINGTIVNKKMIAIKKIKFD.............................DKIQFNEVNNLELIKNKNK.........EMQISSIIDYFG
DDB0229378       31 WVIERQLSKGSFGQV.YKAHKKLDPN.......FVCAIKVIQYC...............................KFTMKEVDYLKKLN.........DPKFVKYYSLEF
Mek1            292 LKIIRVLGRGAGGVV.KLAYHETSGT.........YIALKVITLD.........................IQENIRKQIILELKTLHKTS.........YPYIVSFYDAFY
MEKKalpha       495 WQKGQILGRGGYGSV.YLGLNKDTGE.........LFAVKQLEIV.................DINSDPKLKNMILSFSKEIEVMRSLR.........HDNIVRYLGTSL
DDB0229911       71 YELKETIGKGGSGLV.QRAICLPFQE.........NVAIKIIDLE.........................HCKNVSLEEIRKEIQAMSLCH.........HPNVVAYHTSFV
DDB0230012       97 YNLIEPIGEGTEGRV.FKAYCIPLKE.........NVAIKVVELD.........................KMDPQFVKDVIKEVKVMNGNN.........HPNLIHYHTSFL
MkcA            585 YTFREKVGKGGFGTV.YLVRNNMDKSK........SRIAIKKLPHV.........................RKKEKKFNVKEIRVLEFTN.........HPNIITYYNSHM
MkcB            438 YKDSDQVGKGGFGTV.YFAKSTKEKR.........LVAIKKMPHV.........................TKRQQQQNFREAAILAKCD.........HPNIVKLHTCHI
MkcC            616 YKNLKQIGSGGFGSV.FLAKSTVDKC.........EIAIKKIAHV.........................SAKAQRTNLNEIGFLNFCK.........HPNIVSYLRSHL
MkcD            369 FKNLDFEARGGFGSV.FCAKNKNPHSAYD...KQMVALKKMPIK.........................TLRHKRMNLSEIGFLKYFN.........HPNIVKFLCAYQ
MkcE            429 GENAEV..KGAFGTV.YQVFYVNGQYN.....NVDVALKKMDHK.........................SEKKRRNNLNESILRYLK.........HPNIVTYINSYE
MkcF            401 IKFTHMVGRGQYGKV.YDALYDKKR..........VCVKVVNYS.........................TPKEQHNVLQEIGFLTQCD.........HPNILKYNCSVL
PakA            911 YYNINKIGEGGAGEV.FEAINSRTNQ.........TIAIKKMKLK.........................AQNLKTVINEIGMMKNSN.........HENIVQYIDSYI
PakB            570 GEGSTKIGEGAAGEV.FVVTQLKTNN.........KVAIKKMPLN.........................QQNMKLIVTEIGIMKSCR.........HQNIIDYIDSYL
PakC            205 YKNMTKIGEGAAGEV.FVATSSKNNK.........RVAIKKIEIN.........................NDNAKLLVTEIAIMKTSH.........HDNIVNYIDSYI
PakD           1376 YKVREVVGGGSTGKV.YVGENSITGE.........KVAIKKMKVD.........................NNNIKNIINEISTMKNSK.........HKNVIKYVSSHL
PakE            650 FEFKEKLGQGGYGAV.FKVLHRETNF.........PLAIKVLSIT.........................PTRIKDIEKEIDLLKKCR.........CPNVLSYYGSIS
PakF            394 FILDEKLGDGAYGSV.YKGTHKDLGF.........TLAIKVIEMK.........................ESESVSLQNEINILKNCK.........SPNIVSYFGSLQ
PakG            139 FLIDCILGTGSYGTV.YKAIHKDTNF.........VLAIKSIPIK.........................ESEEIEKEISILKKCK.........SQNIVSYFGSGQ
PakH             42 FEIQEKLGEGSFGSV.YRAIHKSSNT.........SIAIKEFEIF.........................EANDVEPISKEIQILKKCN.........NPYVVSYFGSIM
Krs1             20 FTIVEKLGEGSYGSV.YKAINISTGI.........VVAIKKVSVD.........................NDLEDMEKEISFMKQCK.........SPYIVTYYASFR
DDB0216375       23 FDLIECLGRGSFGSV.YKATYKKTGN.........IVAVKLVPIN.........................EDFQEILKEINIMKQCK.........SKYVVQYYGNYF
SvkA             12 YVRQEKIGKGSFGEV.FKGINKKTNE.........TIAIKTIDLE.........................DAEDEIEDIQQEINVLSQCE.........SPFVTKYFGSFL
DDB0216374       21 FRVLEVIGQGSFGVV.CTCINTVNNE.........VVAIKFLEME.........................GEENSSLKKEITTILKNTVR.........CPYIVKYHGCYI
DDB0216379       23 FQIVEVVGSGSFGTV.CACRWMKKKDRES.NGNRLIACKFVEVN.........................ADDVETNLNLVKEIDILKESMD.........CPYIVEYKGCYL
DDB0216377       29 YHIQERLGKGSFGQV.FKAVHFANGK.........VVAIKIISLD.........................DQEAIKDVRKEISILAECN.........YPNIVQYFGSYF
DDB0216378       20 FELIEEIAEGSFGTV.YKGKHLPTGN.........IMAVKIIALD.........................EDETFEDLVVEIDILNRCN.........HNNIVKYYGSWV
DDB0230010      935 FRDKIKLGTGAFGNV.FYAIRKSDSS.........PVAIKVLMER.........................TKKDSPIIPELYIHSACN.........HSNIVTYIESYL
DDB0229972      173 YESPPTLGK..YDKV.ILVILSQQYLQN....QVNIFPIKLIDRE.........................RINLIHQNILSNEFQLLKSLK.........HSGIVNYIGMIN
DDB0229973      848 IKLCEQIGSGGSGCTVHRCTVDGFT...........CAVKVLKLK.........................NTSPFLVEQFISEITIMVKLS.........HQNIAKYLGHRL
IfkA-a          449 WKKGSCIERKSNYSV.YRGVDEDTDRKLIC(55)VVELKKRLIE.........................KVKMIQKEIESMKYLG.........NPYIIKYLGTNF
SAMK-A          191 YEYVESISLGVFSVV.GKYKRKGQENE.......FIAIKKIDIL.........................SLNEEKIIKEINKLYSIN.........HPNIIKIIGYCK
SAMK-B          186 YKLIEEIGRGAFSIV.EKYENKLKPN.......EFISIKRINVL.........................ASEKEMIVKEINMLYSIN.........HPNIIKIIGYYK
SAMK-C          181 YVFIKQMKGSVNCSL.EKYINKKTKE.........RILIKKIGKS.........................NINEETIINEISILCSID.........HPNIIKTYGYYK
SAMK-D          136 YIETISKNKFCEIEKY.KKSQTKVNEY........IIIKKIIKN.........................STLNEEKLINEIDTIYLLD.........HPNLIKIIGYCK

DDB0230038      521 AQPSDIIGSGNNGTTQRAGIHKDKR..........IVMKQWNFI.........................TSQATPMLFNEIEQLVAIK.........HPNILALAGASF
DDB0230124      961 IQVGSRLGLGSFGDC.YTGNAFSIP..........TILKKLRTQ.........................RFTDQFLGQFKNEVMQIRELQ.........HENLVPVSGCCL
DDB0229871b     695 IKTIIVKDSYSILEGQYKGKL...............VSIKQINGS.........................INDFEMKQLGVLASIK.........SPLAVRFIGVVF
DDB0229850       51 IEYVCRLGSGSLCRV.YKGRLNGKP..........VAIKVFSPI.........................RFEEFKTEFLMMQSLRS.........SPFLISFYGVSI
ARCK-1         1112 LEYTEKIGSGASGKV.FKGIYRGRV..........VAIKVLKSA.........................DDEMTREDFLKEFGVLASLE.........SHTIVGLYGVVL
```

```
                    Subdomain I                        Subdomain II                                         Subdomain III              Subdomain IV
SAPKalpha     351 LEYTEKLGAGSSGKV.YKGLYRGKE..........VAIKVLKSM.......................................TESKEIEEFKKEFQIMSAIR..........SKHVVHFYGAVL
DDB0229849    529 LEYTLKLGSGSSGKV.YKGLYKGKE..........VAVKVLKSI.......................................TTQSQLEEFKKEFQIMGSIR..........SQFMVTFYGACI
DDB0229848    601 LTYNVLLGTGASGKV.YKGTFNGQE..........VAIKVLKSF.......................................TDKKDIAEFKKEFQIVSALR..........APGVVYFHGAGI
Phg2          807 LEYIKRIGSGTYSKV.YKGKYRDKF..........VAIKTMRGS.......................................NMTTEQIESFKKECDILSTIQ..........SPLLISFYGSCI
DDB0229847   1949 IVFLNKLGEGTSAKV.YRATWRNQE..........VAVKVLRSE.......................................PESQKLLDFLKELEIMSSLR..........SPHVVYFYGMVL
SplA         2058 LEFGQTIGKGFFGEV.KRGYWRETD..........VAIKIIYRD.....................................QFKTKSSLVMFQNEVGILSKLR..........HPNVVQFLGACT
DPYK4-b       299 NKDDTVGGDGFFSVV.KKGYYNETE..........VAIKLIKKA.....................................HGENVTVCDTFYHEVLIISNLR..........HPNIVQFIAACI
DDB0229872    177 LTVEKEIGQGFFGKV.YKARWRGKS..........VALKKITLI.....................................KFRDLTETEIFDKEVSIMSKLC..........HPTCVMFIGACS
DDB0229844   1476 LFLIKKIGAGSFSKV.YKAKYMGE..........IVAIKVLKGE.......................................ATSEQIELFKKEYDILSLVS..........SQNLIKFYGACK
ZakA-b        379 DKDDSEGGAGNFGDV.KKGLLNETE..........VAIKFVKKA.....................................HCEAITVCDTFYHEVLILSNLR..........HPNIVQFMAACI
7TMK1         363 FKFGQVIGEGYFGEV.RKAVWKGAV..........VAVKILHRN..................................SFRNTDGNKEENVFLKEVAILSILR..........HPNVLQFLGVCS
rk1           374 IKIGVRIGKGNYGEV.YLGTWRGSQ..........VAVKKLPAH.......................................NINENILKEFHREINLMKNLR..........HPNVIQFLGSCL
rk2           391 IKIGVRIGKGNFGEV.YLGTWRGSQ..........VAVKKLPAH.......................................NINENILKEFHREINLMKNLR..........HPNVIQFLGSCL
rk3           491 IVVQNRIGRGSCAEV.FTGTWRGI..........IVAIKKAKLL.....................................NEDDEDFLNELAQEATIMSQLR..........HPNICQFLGTCN
DDB0214883    851 IAIGARIGRGGYGQV.FRGSWRGTE..........VAVKMLFND.....................................NVNLKLISDLRKEVDLLCKLR..........HPNIVLFMGACT
Shk1           45 ITTESILGDGSFGTV.YKGRCRLKD..........VAVKVMLKQ.......................................VDQKTLTDFRKEVAIMSKIF..........HPNIVLFLGACT
Shk2          174 YNREAKLGSGAFGSV.YKGIVRGKE..........VAIKKLTQT.....................................VFEENTMNEFKKEVSLMAKLR..........NPHLLLFMGACT
Shk3           24 INFEELIGTGSFGKV.YKGRCRQKA..........VAVKLLHKQ.....................................NFDAATLSAFRKEVHLMSKIY..........HPNICLFMGACT
Shk4          277 IDRTDFLGQGSFGSV.YKGKCRGQE..........VAVKIPRKQ.....................................KLSLYELTSFRHEVKIMSKIF..........HPNVVLFLGACT
Shk5          237 DVKTDLLGGGAYGKV.YKATCRGKK..........VAVKVPKKQ.....................................TLSESELKSFKNEVEIMKQIF..........HPNVVLCLGACT
DDB0229963   1073 IKIDKEIGKGHFSKV.IKGNWKGKD..........VAVKKLNSN...................................KDKAREEMIQEFKAEVELLGSLQ..........HPNLVTCYGYSL
DDB0231199   1377 LQILQFLGEGALAEV.HKGIWKGKE..........VAVKIFNEG.....................................SFSFRLEDFLKEVAILGLIS..........HPNLLKLKGACI
DDB0229940    386 VAFGDVIASGASGKV.YKGIYKGRD..........VAIKVYSSE.....................................NFCFNIEEFDREVTIMSLIDS.......DHPNFTRFYGANK
DDB0218878e   702 VIFGDVIAAGASGKV.YKGIYKGRD..........VAIKVYSSE.....................................NFCFNIDEFDREVTIMSLIDS.......DHPNFTRFYGANK
Rck1          842 VSIHKWIASGSSGRV.YNGQYKGKD..........VAIKVLGPE.....................................VCVHFDLNEFKREVALMSIFK..........HDNLARCLGAGQ
DDB0231197    599 VKINVEIYDSPLCTV.YSGVYNGMD..........VAIKEFSQD.....................................GMGFDWVSFYKEITIVSASQ..........HPKVIKCYGAHT
DPYK2         853 IQFIQKVGEGAFSEV.WEGWWKGIH..........VAIKKLKII.....................................GDEEQFKERFIREVQNLKKGN..........HQNIVMFIGACY
DPYK3-b      1057 VAIVEKVGAGSFANV.FLGIWNGYK..........VAIKILKNE.......................................SISNDEKEIKEVSSLIKSH..........HPNVVTFMGACI
Gdt1         1372 IHFQELIEKYSWEIL.YRGTVGDKN..........ALLKLIKLK.......................................TKNCEEAYRELNIISRLK..........HQNILPLIGCVI
Gdt2         1290 IIIKNYISEGTFGIV.YRGIWRSSP..........VAVKLLKNE.......................................YVDHDEIEKEISLLERLR..........HPNILLYVGRFI
Gdt4         1349 IVLEKYLSEGSFGVV.YSAIWRSSS..........VAVKLFKHH........................................YSKDEIDKETSILSKIK..........HPNIVSFIGSL.
Gdt6         1194 IEVKELISQNNLSTV.NIANYRGLQ..........VVLRKIKTH.................................SANYTKTYSQAIKKFYTEMDILSKLK..........NKNIVSFIGCTH
Gdt8         1038 TEQLIASYLPSKVYV.FKGYYRDLA..........VAVKFAIS.........................................DNSKSFEKIKNEVQILSKVN..........NINVVRYIGSAQ
Gdt9         1290 LDFDEICGQGTYGMV.YKGKWRSQV..........VAIKMMRVG.......................................TVNCDEVYREMDIMGKIR..........HQNVISFIGCVV
KinX           22 LDFISEIGSGGFGKV.FKGEYLGAP..........VAIKKIHIL...................................PDDPNRVDLEKFLNREIETIKLFT..........HPNVIQFVGISE
KinY           32 LKVGESIGSGAYGIV.YRGTLFNSD..........VAIKKIQNE.....................................KSEKNEFIKYLKREVAVLKNIQ..........HPNIVQFIGVYY
DDB0229853     33 LFMDVEIGRGSFGQV.QKASYFGTD..........VAVKQLSTL.....................................VSIDPDYFKFMLREIKILKGMR..........HPNIVQYIGACC
DDB0229863    336 ..VGNEIGSGKYGSV.SLGYWLGTP..........VAIKKLHEN.......................................NEETEILVQRELQILKEIR..........HPQIVQFLGVSR
DDB0229866     35 LEFGQEIGKGAYGKI.FKGEYFGTP..........VAIKEISLQ...................................PNDVKYKDLTKFIQREVAMLRFS...........HPNLVQFIGVSE
DDB0229851     23 FDPLSIIGSGGFGKV.YQGVLNGKE..........IGIKKITIS...................................DNDPNRDILLKFLEREIYTLKMLS..........HPNVIKFYGIAE
DDB0229865     15 IEENEF.SKGSFAKV.YKGSYYGNP..........VCVKVIKKD.....................................TLIDKESWVFLKREIGILKNLLNQ......GHKNIIRFIGIGE
GefX           21 LEFNEKIGKGSFGSV.FRGCYLGLD..........VAIKKIEKA.......................................DDPEYLKYIDREVSMLQSLR..........HPFIVNFSGICV
DDB0229867     18 LVFKDIIGKGNFGCV.YRGNYLGVE..........VAIKQIPSF.......................................DDPDYCKYTEREVKALRYIR..........HPFVVHFFGACK
HH498         530 FKTARKIGAGAFGDV.YLVEWRNKN..........VAVKRVKIE.......................KILESGKSYQWIRDKFILEAVLMVKLSN.........FSSFVKLYATCI
GbpC          878 VKINKEVGRGAFGIV.YEAEWMSEW..........IALKKLLLP..SSTDTTNLNGNGEVMVYDDPDQLIEDRLRVFREFRHEVYYMSGLN..........HPNVMKISGFCI
Pats1        2247 LMIEELIGEGGAALV.YRARWQGQT..........VAVKKLKTI.............................ENLDSPIEINDISLSKAFNEFRRECWVMSELE..........HPNIVQLKGLCL
QkgA         1242 ILYERQIGEGGFGLI.HKGKLISNKS........DIAIKSLILCNSNNNNNSNNNNNSNNSNNFKNNNNEDEFIEKFQEFQHEVFIMSNLN..........HPNIVKLYGLMH
Roco4        1026 IEYEKQIGKGGFGLV.HKGRLVKDKS........VVAIKSLILG.............................DSEGETEMIEKFQEFQREVFIMSNLN..........HPNIVKLYGLMH
Roco5        2175 LEIIEKVGEGGFGIV.YKGKLRGQ..........LVAIKQITID.....................................SGQAEAASEIYREFRREVWLSNTLT..........HPSIVSLKGYCL
Roco6        1356 FESPKLIGRGASGKI.YRANLNDTM..........VAVKQLEVV.....................................GEDAPRIFSEFRREIHVMSDLK..........HSNVVNLLGFTL
Roco7        1775 LKFGPQLGSGSYANV.YRGIWNGSE..........VAIKVLNFD.....................................DGHANTTEKYREFRNEAHITGELR..........HANTVSLMGVSL
Roco8        1456 LIYQEEIGVGGFSRV.YKGIYKNNT..........VAIKQFNFE(120)LSTSSLSSNSSQGEDSMNQISSGKLNAINEERREVWLMSGLS..........HSNIVLMKGFC.
Roco9        3008 ELDPNPIGEGGTATV.YKGKWRQSD..........VAIKLLKTD.....................................VVGSDFSKVFAEYRREIFCLSSFI..........HDNILDLKGFCL
Roco10       2069 HSNQNENGTSTQSMMGMYGKQF.............VNIKVFNAP...................................LLGADFQNNCARILSSFRHEALSLYNFR..........HSNVLSIIGISM
Roco11       1185 VVCEEQIGVGGFGLV.HKGKLILQDKS......LVVAIKSYIVG.....................................NSSASDIIRKFQEFHHEMYIMSSLN..........HLNIVKLFGSMQ
DPYK3-a       726 IKLLEKLESNSKSSNIWQIEYKSTQ..........LVLKQPKDQ(11)LFNGSNVSGSNNSGSSGGNNHNHHHCNNSNGSNSEVIPSKYTMIQ..........HKNLGLLVGWCG
DDB0229956    758 LELTKEIGRGVSGVV.KRGKWKGYE..........IAVKQFNEE.......................................ELGFSEREFHSEATIMSVLR..........HDNIVHCVGGSA
DDB0229957    986 VVLHERLGTGATGDI.YRGLISQDNLNRHI(42)IVAVKKLHPL.....................................ADPSPEMIQDFYNEVRVLSMFN..........HPNVVKYVGGCT
DDB0220138    946 IQFFNLIGQGGFSKV.YHGVWRSKD..........VAIKQIELQ.......................................NNKSLDDFRREVGILSKLKP..........HENLLAYYGACK
DDB0220436    345 FNDSNKIGEG..GQC.SIYKYMG...........TAMKRFKPS.......................................LSSSLISKEFENEVLILERLN..........HPNIVKIITYST
DDB0230133    192 LEIGNVIGGGGYGAI.YKAKWRGLS..........VAVKVISEL.......................................SDGSEFEKELEMHKELLH..........HPNIVHFVGFCV
DDB0229871a   360 LLFIKKIGSGACGEV.CQYEWKGTP..........VAVKTIFKS.....................................LLRKDKKEEFEKEVSILKCLR..........HPNVVLFMGTCL
DDB0229955    662 LKISSKLGEGTFGVV.YKGLWRGSS..........VAIKQIKIN.....................................EDVNNQVLEEFRKELTILSRLR..........HPNIVLLMAACT

Bud32          35 DEIGILISQGAEAKT.Y.....ETD.........LYGLKCIVKE..........................RFSKAYRHPILDQKISSKRILQEVRSLNKCK..........KKGIQVPSLYLV
DDB0231196    459 IDFHTQIGSAGNASV.FEGTYKGIP..........IACKEMPVS.....................................GTYEQSVDSIKEIAAVGQIKK......LGCATVVETIGVLK
```

```
                          Subdomain I                          Subdomain II                                                  Subdomain III                   Subdomain IV
DDB0229339    482 IDFHTQIGSAGNASV.FEGTYKGMP..........IACKEMPVS...........................GTYEQRVDSIKEIAAVGQIEDL......GGCTVVKTVGVLK
DDB0231195    777 LSDFSIIGEGGFSTV.HKATYINLQNT....IT.PVAIKSFKNY..........................DDDSFERIYKEVSVHQSLQ.........GENILKLIGVSK
DDB0229335    312 IERRNELGRGGNGTV.YSGVLKEIDSQGN.EISIPVAIKIPTQF..........................YKSKLVEVYKELAIHQKIN.........GICGPKLFGCVK
DDB0216331      4 YEVVKLIGVGGEAKA.LLVKRINSDK.........LYVMKQRMFF..........................LLEEANEGLCEAMSLAKIQ.........SPYIVRFEEVFL
DDB0229344      8 YKLIKDLRSGGEGKA.ILYEKDGVK..........YVGKKR.........................LFDNLKDANQGLKEAMSLARIV.........HPNTVRFEDAIM
DDB0231281a    25 EERLISIAKDKSGKL.CVLKEIKY...........LNNK...............KKLEE.FKKEEKNEEGIQKRIYQRNFNLEFNHLKNFSQ......MNHTSIIKYIEHIE
DDB0230037a    27 ..............V.YIVLNKSNEK.........FICKEIIYT..........................QNSNIDNEKRKSEYINLKKFSDTI...KYPHLNISKYIEHFI
DDB0219988a    24 ..............V.QLVKNKKDGK.........LYVCKTIDFN.......................NNNETKDSKVKREREYLFLKQYSSP...SFSHLNIAKYIKHFE
DDB0231281b  1328 GKNYGGIDLSNPRSLGTYNLIGDSVFR....NISFFENKLFEK.......FDTNVNFQKEIEFVKNYSKFLNFDISNRIPREYSILKSIK.........GMNGVVQIDSWYL
DDB0230037b  1187 EVGSISKIREWKK.FGIISSHPDAV..........VFLKYGKKD.........LKSAMLDNGKVNDENYYGIAVEDYMKSNELNILYYLLSK......QEEPDFTNIERYYL
DDB0219988b  1108 .............V.WLIRDKNNNNYIR....KLSGYIKSKFKE(16)PYLKIAVENGWPISFLSSHFKRDNSNLFDYLFNKELIILELLKE......KGVDGISQLESYFV
DDB0231182    186 INNNMQKTGGRNGSV.FFVNNNNNSNNNNN(52)TFAIKQQLVS...........................TPKNKLSQTYREKVILEHLNILI..ESNKACNFPKLINWYK
IksA          261 FKEDIKIGSGGFGSV.YLCRHLINGVD.....LGEFAVKKVPVG...........................ENLPWLFRVLREVKALETLTK.........HRNIINYKHSWL
IreA          575 IITNKILGTGSCGTI.VYEGKMEGRK.........VAVKRMLSQ...........................FVKFADREVSILIHSDE.........HTNVVRYYAKEE
IrlA          987 RDENNIIGRGSNGTL.VFRGIWNDRI.........PVAVKQMQKA...........................FNPHISKEIEVLIRLTSN......NCSNMIRYIDQEE
IrlB         1027 RNESNILGRGSNGTL.VFKGLWSDKI.........PVAIKQMQKA...........................FNPLINKEVEALISLTSK......NCSNMIRYIDKEE
IrlC          981 RKEENVLGRGSNGTL.VFKGIWNNRI.........PVAIKQMQKM...........................FNPLISKEIEVLITLTNK......NCYNIVRYIDQEE
IrlD         1054 RKDEFIIGRGSNGTL.VFKGIWNDRI.........PVAIKQMHKA...........................FNPLISKEIEVLITLTNK......NCNNIVRYIDQEE
IrlE          903 KKESNILGRGSNGTL.VFKGIWNNRI.........PVAIKQMQKM...........................FNPLISKEIEILIGLTN.......KNLNLVGYIDQEE
IrlF          950 RNESNILGRGSNGTL.VFKGIWSDKI.........PVAIKQMQKA...........................FNPLINKEVEALITLTNK......NCSNMIRYIDKEE
DDB0216373     22 YTVNRILGEGGFSFV.YLVKDNSNSK.........KYALKVMICQ...........................TQESINTAKREINAFQTFN.........HENIMKLIDHSI
DDB0229347     54 VTEVKLVAEGGFGFV.YLVRDDYNNM..........YALKRMFIQ...........................ERERLEAMKNEIDVMQKLRN.........NPNIVKLEGFKI
DDB0229350     16 LNFVKQIAEGGFSYV.FLVKDSNTSK.........HYALKRILIR...........................DEDELKGVKHEISIMKRLTK.........HKNIVKILDYHK
Nek2            4 YEILGALGKGSFGVV.SKIKRKEDGR.........VLVWKEICYE...........................NMQEKEKQLLVNEVNILQKLK.........HQNIVRYYDRII
Nek3            4 YEEIKTIGKGSFGRA.ILVKRKSDGL.........LLVLKEINVM...........................EMQPKERSDAMNEVNLLSMLD.........HENIIGYYDSFI
Nek4            4 YDIIKQIGNGSHGDV.YLVRSTIDKK.........KYVMKKIFLK...........................EREKTKDTLHEVNVLSQLK.........HPNIVEYFESFQ
DDB0229345     12 YLIKSQIGSGSYGNT.FKVTHKESGQ.........VFCKKSIQVN...........................NQENIEKVLEEGKILTVMD.........HVNVVKLNDSFF
IfkA-b       1024 FEEIEMIGKGGFGVV.VKSRNKLDCR.........YYAIKKIKTK...........................GYTDSNQEPLTNKLLREVTTLSRLH.........HQFVVRYYQAWI
IfkB            1 .....MIGKGGFGVV.VKSRNKLDCR.........YYAIKKIKTK...........................GYTDSNQEPLTNKLLREVTTLSRLH.........HQFVVRYYQAWI
DDB0216407    494 FEEIQLLGRGGFGQV.VKVRNKLDGR.........YYAIKKIKL...........................DSNQS.LNRRILREVITLSRLH.........HQHVVRYYQAWI
DDB0229432    379 FKEISKLGSGGFGSV.YLSEYVLDGH.........KYAIKKVNFS...........................ISNNQSPTNASSKIEKVVREVVALAKLD.........HINILRYHNAWL
DDB0220611e    83 FCEIEKVGKGGYGSV.FKVKNKLDGE.........YYALKKIPFK...........................NTTKTFLEKVLREVKTLASLN.........HRNIVRYHSAWL
DDB0220652    236 FNDVRVLGKGGFGIV.FQCCNIFDQM.........EYAVKRIKVN...........................QKIPTKELM.EVRAMARLN.........HPNIVRYYGSWI
PLK           163 YRQGEFLGKGGFAKC.YLMTEVETNR.........IYAAKIIPKS...........................TLQKTRARSKLKSEIKIHSSLS.........HENIVKFEHCFE
Vps15          21 IVFKKSLGNARFLKT.VKAYHSEEGY.........VVVKIYKKR...........................NTKESLEKYKIMLKEIKDNFNITP.........SFNIMPYQHFIE
TBCK           77 NQNNGSIG.GSSSSS.STSNSVSTPKPQSFYVDDAGIAKQLNQS...........................VFDILSRSRYLSSLS.........HENLAEFIAAEP
TtkA          714 YLRIEFIGKGGSGKV.YK.VLSGDLK.........IYALKYVCLS...........................DPNEIEAQLNEIEMLKRLRK.........QVNIIQLIDYEV
DDB0220004    508 FQELDLIGEGSFGHV.YKVRHRIDGC.........LYAIKKTKKP...........................LKGQKDRDIVLREVYGLSAIKD.........HTNIVRYFNAWE
DDB0229384    177 FDVVCKLGSGSFSDV.FKVKSKFDGN.........SYAIKQARHQ...........................FRGFQERERAVREVKAAVSLPP.........HTNVLQYYSSWE
DDB0216398     77 FEYINQIGEGSFAKV.YKARSFVDGR.........LYAVKKSKKP...........................IWETSERNQHIQEIENGMKLGH.........HNNIAQVMCAWE
Scy1           22 GPIVTGYVGKSIWTL.HSGTKKEDG..........SAVSIFSFD...........................IKKNPSKLEVAKNGFKRAKTTR....HPNVLKYLDGLE
Scy2           30 HDLKEVVGQDKFWKI.YQSTKKTTNTEC...SLFVFEKKLYEKV...........................SKSNLENVITFLKKEATTLQRLRHPSILQVVSVME
SLOB1         129 ...KNSMGGGEECVV.SIITPTPSSC.........PWS..MSNE...........................KKKRKFEKTLL.SLK.........HPYILTPINVEV
SLOB2          90 KVYFGVLGN.......SLNKLENDRI.....MAMVPVSKHWPIP...........................LNSEAGRTTFRTIIKSLE.........IHPFISVPLLVDF
DDB0229334      1 ...................MDIE............CDIRKINKL...........................NYNNENEIYKLKKLN.........HPSIIKHYDIDE
DDB0231326   1730 FKDLKRVAKGAYGTV.YKGTLGNDEG........LEIAVKLMPVP...........................KTIHDRCVLYDIFTEILIMDTFRS.......DSRGCHMFDYGV
DDB0231559    508 SELGKLIGKGANGKV.YELHYNFGGVEK......HCAVKEIKVD...........................KYRVGAVLKEIESTALSQ.........SPFTVGIYGYFE
DhkG-a        366 YNVLSLL.NGVGGCA.SFTNYNLNYN...............INNS(42)SASAFKPFQLNSSTNSTGSPLIITSQPMPFQLNSNSNTTASSSSP.....ITHSNLNTAITSTT
DDB0231198    865 ETEIEPFASGGQANI.YMVKHIDWPMLSN.CPEGSYVFKQFIET(19)YQTYKTGESVDNFKYNFSNVEQTDKKSLETLFYEEVDKLKRLQ........FSDYIIKMPAISD
DDB0231179    136 QQNRVLIGEGHYGKV.YRSMYVSSDG.......RSLVNKMLKG...........................KRECTKEVEALIDVFD........ASKMVTTQYFT.T
DDB0231335    355 IQFDALQSNGMNGIV.KSGT.I.............GSMKVVVKF(93)NNNNNNSNNNNNSDGSSGDDNRNSDNLIKSDLIDECFLNETVS.........H.LLINEFQIN.
7TMK2         317 IAIHELIGMGGSGAMVHKCTVKKGPLR.....GGTFAVKVMKDC...........................TNEDIESLENEIRVYEKLK.........SPYIVSYQGSSK
DDB0229381     13 YNNIKELGRGVSGVV.YKASHKKTGQ.........IVAIKLVDMK...........................QSKISTEQIQGEIRALLTLNPEN...ERTHINIIKLIQCFI
Bub1         1003 ..................................WLSCKVQDPPSIW..........................EFYIGNQIHQRLQERSLTISNSK.........FPTIHSLYYYTD
DDB0230007      8 .........................................KRIKSD...........................HQNQNQDQINYVDE.NVKENLS.........TYYLVKRISFE.
DDB0229337     57 .............................................................................................................
DDB0229346     72 ..........................................................................KVQKKSNKAMKELTVMQKIG.......YYSNHVVNVYGYFN
LvsG          403 .............................................................................................................
DDB0230125    364 ...................................EFVYKWFNYP...........................YFFQTEVKYLTFID..........GIEGTPSI....
DDB0204911e   420 ...................................EFVYKWFNHP...........................NFFKTEVKYLTFID..........GIEGTPTIKQQ.
CK1             9 YRISRKIGGGSFGEI.YLGTNISTNE.........EVAIKLEPAK...........................AIHPQLLFESKLYKIFQ.........GGIGIPAVKWFG
DDB0216336     16 WTVVKKIGQGAFGEI.FSGKNIINNE.........QIAIKVEKVD...........................TKKQVLRLEVAVLKKLQ.........LCPYVCRFITCGR
```

| Sequence | N-term | Subdomain V | | Subdomain VIA |
|---|---|---|---|---|
| Akt1 | T | EDKLYFILDYV.NGG | ELFYHLQ | KDKKFTEDRVRYYGAEIVLALE |
| DDB0220670 | T | QDSLCMVLDYI.GGG | ELFYHIS | ERETFSEETARFYIGQIMLAIG |
| DDB0216386 | T | KKKLYMVMEFL.HGG | DCASLLH | SLNTLEEREAKNIIAQIVNGLE |
| DDB0220701 | S | TDKLYLVMEYL.IGG | DCASLLR | ALGCFEEHMAKHYIAETVLCLE |
| DDB0220700 | T | DKKLFFVMEFL.NGG | DCASLLR | SFNNNMPEDLTKNIIAQIIICLE |
| DDB0185113 | T | KEYYYLVMEYL.QGG | DCFSLLQ | MLGSMDENMAKMIIAETVLALE |
| DDB0229452 | SP | CKEKVLIEMEYL.SGG | DCAFHLN | DVGVGFPEELAKQYTAETVLCLD |
| NdrA | D | DNYLYLIMEYL.PGG | DMMSLLI | KYDIFTENQARFYIAETILAIE |
| NdrB | D | ANFLYLIMEYV.PGG | DMMTQLI | KYDTFTEDATRFYIAETVLALH |
| NdrC | D | ANYLYLAMEYH.CGG | DFRALLN | NLGTLSEDEARFYMIEMIEAIS |
| NdrD | D | SQYLYLAMEYH.CGG | DFKTLLN | NLQRLEDDIAPFYMAEMILAVE |
| DDB0231558 | D | DQYLYLAMEYV.PGG | DLRSLLS | ALNSLDEDSARFYMAEMVEAVD |
| DDB0216387 | D | NEGVFYYVMEFI.AGG | DFNSYCYKRLV | DGKKFTEEEIKFYIAELVLCLE |
| Pdk1A | S | EQCLYYVLELC.SQG | DLLHQIK | KVGSFDYRSCQYYVAEIISGLE |
| Pdk1B | D | ENNLYFILEYC.PNG | DLLGALK | KAGCFSIDVVRFYAAEILIALE |
| PKA | D | EKKLYLLFEYV.AGG | EVFTHLR | KSMKFSNSTAKFYAAEIVLALE |
| pk4 | D | DRYLYFLQEFI.PGG | ELFDYIR | ANGSLSLYVTQIYAAEIVLALE |
| DDB0185224 | T | PTKIHFIMEYA.GKK | DLFHHLR | ANKCFTEQTTKLIVAEIVLAIE |
| pk2 | T | KDKLYMVLDFV.NGG | ELFFHLK | REGRFSEPRVKIYAAEIVSALD |
| DDB0220702 | N | EKKLYLVMDFV.NGG | QLFYHLQ | KEAIFSEDQVRFYMAELILALE |
| pk3 | N | EDFLFMCMDYV.PGG | ELFHHLQ | KAGKFPEELAKFYIAEVICSLH |
| Aurora | D | DKRVFLIIEFA.KGG | ECFKELQ | KVGSFNEQTAATYTLQIADALR |
| MLCK-A | T | PEKLYLVMELV.TGG | ELFDKIV | EKGSYSEADAANLVKKIVSAVG |
| DDB0216385 | S | DEDICLVLEWI.PNG | DLFDRIV | KKGVFNEEEARLTMKSLLSAVE |
| DDB0216309 | T | TKHLHLVLELV.TGG | ELFDKIV | QRGEYSEQDASKIVRQIVSAVG |
| pXi | T | DNEVLLVLELV.TGG | ELFDKIV | EREFYTEQDASTLIGTVTKVIQ |
| DDB0216308 | S | NDYLYLVLELI.RGG | ELFDKIV | EKGNYSEKDACNLVRQIVSAVE |
| DDB0229351 | S | ELYYYIVTELL.QGG | ELLYQLEK | NHPSTNENVSNYTEEHARKIIKQVIQAVG |
| DDB0216307 | S | DSHLTLVMELV.TGG | ELFYKIV | ERGSFTEKGARNVVRQVCAGVE |
| DDB0216312 | S | TDKIYLVVELV.TGG | PLFDRIV | DKKSFTEKEAKLITQQLLQSLV |
| Snf1 | T | TTDIFMVMEYV.TGG | ELFEYIV | KNGKLLEDESRRLFQQMISGVD |
| DDB0229364 | T | SKYLFLILEYV.EGG | ELFDYLV | EKGGLESGEALFFFQQIIIGLE |
| Lkb1 | IE | EKGKLYIVYEYV.GGG | TSQNILE | NAPNGRLPPHQSQFIFRQLIEACE |
| MARK-A | T | SRALYLIMEYA.GEG | EVMDFMI | AHGVLTESQARTFFTQIVSAIN |
| MARK-B | ED | DVGRICLILELV.SGG | ELFDYIV | ARGRLSEKEGRKFFRQMLCGLI |
| MARK-C | KR | EEEGTTYLIVEYV.SGG | ELFDYIV | AREYIKEKEARKFFRQMISAIE |
| DDB0231454 | K | DDAMFILMDYC.QGG | DLFHYVNKFGLKGVK | MVNSPDIGDQVLMGVPLPEDDTKRIFTQICLGIA |
| DDB0219986 | | DRNAFMQMEYI.SGG | NLLDWCRN | NNNDNENNKRRQPWEIQKIFQQIIQAIA |
| FHAK-A | T | KDQQLFFILELA.NGG | ELYNKIG | FNEPLLNENQSKFIFKQILNAVS |
| FHAK-B | S | STDCFFIVELA.TN | DLSNLLR | KRCVDEDDIKRICKQLLLGFN |
| FHAK-C | S | DRYFYIVLELA.TGG | ELFEKIK | QKGRFSEPEAKDTFKQILEAVS |
| FHAK-D | T | QKNLYLVLELV.TGG | ELFDKIV | SERKFQEDTCRYILKQLCDSVR |
| FHAK-E | N | EKVLSLILELV.ECG | ELLNDIVS | NLFYTEDKAKTLFRQIVDGVL |
| DDB0220010 | DP | QEEYIYIVMEYI.EGG | SIM | SANETSEDLARKYFRDIVFGLE |
| Apg1 | EE | TDPTFIYMIMECC.EGG | DFSKYIR | THKKLTEEKALYFMKQLANGLK |
| tsunami | N | KNEFTLVTEYA.DG | DLSQIIS | EEKTLSVDLIQSICYQLVIALN |
| Cdk1 | C | QNRLYLVFEYL.DQ | DLKKYMD | SVPALCPQLIKSYLYQLLKGLA |
| Cdk5 | T | ERKLTLVFEYL.DQ | DLKKYLD | ECGGEISKPTIKSFMYQLLKGVA |
| Cdk7 | H | KSNVYLVFELM.QW | DLQEVIE | DKSIILKPADIKSYMKMLLQGIE |
| Cdk8 | NP | KDKLLYLIFDYA.EF | DLFGIIK | YHRENGSHFSDATIKSLIWQVLNGIH |
| CDK9 | SKASA | SNNHKGSVYMVFEYM.DH | DLNGLMDSP | AFKYFAPGQIKCYLKQLLEGLD |
| Cdk10 | GS | TGDKIYLVFEYL.EH | DVASLIDNI | NKPFKLSEIKCFLLQLLRAVE |
| Cdk11 | | GKNINSIFMAMEFI.DH | DLRGLMEVI | KKPFLPSEIKTLIQQLLNGVS |
| DDB0229294 | TNF | ETSEYIIVFPYF.EH | DLSGLLS | EHRLSIPQVKCYFKQLLEGIN |
| DDB0229424 | S | NDDKLVMVFEYL.EY | DLWKFIS | PKNKYMTLSCTKFFIHQLLEGLD |
| DDB0230050 | ID | KNSVLVLIMEFI.EG | DLWKIMS | NPQCTLSLGQIKNFTKQLLEGVK |
| CK2 | DP | QSKTPSLIFEYI.NNT | DFK | HLSPTLTDFDVRYYIRELLHALD |
| DDB0219953 | DQ | SSKVCSLVFPFV.NKT | DIRELVY | TLDDYDIRYYIFELLKAID |
| ClkA | F | KDHICLVFKRY.GL | SLYEFLK | KNRYRPLPLSQIQNISKQLLTAIY |
| DDB0229841 | RKCSDSYQPPLTSILNSIISSNDNPQQHSTLIQQKDDEDFYFESIQP.QY | | SLLNLIS | NNMLDQDDICQLFYEILMGLK |
| DDB0229333 | KSLNIKTKN | LIESPVDDGTDLYFEFDQS.DF | TLYQMIR | SDKAASRKQCPYVLYQLLTTIK |
| DDB0229296 | RKSSENYLISNKNN | RNNVRLPLLQQKGDDDIYFEYLLP.EF | TLLQMIH | NKLLTEYNIMIFLYQLLTVVK |
| Dyrk1 | F | RNHLCIVTELL.SY | NLYDLLR | NTHFHGVSLNLIKKFAHQILTALF |
| Dyrk2 | F | RNHLILTFELL.SM | NLYDFLK | VNHFQGYNLNLVRRFGAQILTSLR |

```
                                                       Subdomain V                                          Subdomain VIA
YakA         H...................................KMHLCIVFELL.SV....NLFELIK................................QNNFRGLSTNLIKVFLIQILDALI
Prpf4B       Y...................................RNHLCIVFEPM.SM....SLHQLIK...............................KYGKDIGLSLNAVRVYAKQLFLALK
GSK-3        TS...............................DNDEVYLNLVLEYV.PD....TVYRVSRHY..............................SMSKQPVPNIFVKLYIYQLCRSIN
GlkA         TA...............................EDNKKMQNLVFDFI.PY....TLASLLK...................................KRQLSINFIKVLFYQLCQAIK
Erk1         PNS..............................KEQFEDVYIVSELM.DT....DLHQIIT..................................SPQPLSDDHCQYFVYQMLRGLK
Erk2         AD.................................NDRDIYLVFEHM.ET....DLHAVIR...................................AKILEEIHKQYTIYQLLKALK
DDB0229430   E...................................RDELFFVFEYL.EN....NLYESIK.................................DRTKLLPETTIRNIIYQILQALH
Sky1         HRG...............................PNGRHYCMVFELL.GN....NLLDLIK................................HHRYRGMPITLVKTLMKQTLIALD
DDB0230051   DE................................SSGKYYSVLSPFV.PK....DLEFILA......................ENSQNFGGLGKISPTTLTFSDIKYIVYQLISVVH
DDB0230126   T...................................PQSIYVIMEPS.TT....SLKPLVE................................SMDQHQLPISMVISIMKQLLQCLL
Cdc7         Y...................................KDQVTLILPFF.EHD...SFKDYFF.....................................QMSNENIKHYLYALFDSLR

DDB0229971   N...................................ENYIYIFLEYM.ENG...SLSTILN..................................NFGTFPESLICNYVENVLKGLV
DDB0229896   T...................................KDNLYIVLEYV.ENG...SLSGIIK..................................KFGKFPETLVCVYIRQVLEGLV
DDB0229877   T...................................SRYLYFALEFI.EGG...SLAKIAK..................................RYGCFQEPLLSRYFSQVLKGLA
DDB0231212   S...................................SSHSYIVMEFI.ENG...SLEKIIK..................................RHGLLPESLVTVYIAQVLNGLE
DDB0229915   V...................................QAQLNFILEYV.ENG...SLRDVIE..................................KFGPLSEELCIIYLYQMLQGLA
FNIPK-A      GE.................................IDDNIYIYMEYI.DGY...PVSSILR................................KQPKNQFPEDIISKIVIDLALILS
FNIPK-B      N...................................KHTTYIFIEYI.NGY...TLEEYVL..................................KNHPIPEKELSEIIEDLIKSLA
FNIPK-C      D...................................EDNGYIYTEYI.QGGI..DIVETVTN...........................KLLKEHSHSFQSEDIIRRCAFQLVLLLN
FNIPK-D      D..................................NNQDVYIYLEFI.EYSI..PISKVKLI..........................PNDSSIDNFNNVSNTIIELVTCKLVESLN
FNIPK-Ea     D..................................RNNEFNIYFEYI.DKAI..PLSRLLE.....................................KLNKKEQFIVATEIIKSIK
FNIPK-Eb     D..................................EDDNLFIYTEYI.EGST..SISDLIK................................KKPNNRFEEEEIKSLMIKIVKALS
DPYK4-a      D...................................DNYIYIYMEYI.EDK...KQL.........................................RFKESEIISMIADLTETLS
ZakA-a       D...................................DNFIYIYMEYI.EGE...NLANILK................................TKKQGRFKESRIISMIADLVETLS
DDB0230002   YGI...............................KGKNKLYIYLEFI.EGK...TIY..................................ELKKKKFPLNEKQISIILKKCLETMK
DDB0229378   NN.................................SKTYAYIIMEFI.EGE...SMKSIIE...................................NKKFSDIEIKEIIKELLKALV
Mek1         T...................................EGSIFIALEFM.ELG...SLSDIMK..................................KTSTIPEPVLGKIAFQVLQGLV
MEKKalpha    D...................................QSFLSVFLEYI.PGG...SISSLLG..................................KFGAFSENVIKVYTKQILQGLS
DDB0229911   Y...................................NESLWVIMDFL.SAG...SCSDIMR................................FSFPQGFEEHVIATILKEALKAIC
DDB0230012   E...................................NNQLWLVMDYL.GGG...SLADIMK................................FKYPDGIPEVLAVTVLKSLLKGLE
MkcA         L...................................HDEVWIAMEFM.EGG...TLA...................................EASNQYPFQESNIAYVAREVLQGLA
MkcB         D..................................KDSNLWIVMEFM.EGG...TFE...................................EAAKAWKFNENNLAYVAKELLKGLQ
MkcC         V...................................DDTIWIAMEYM.QGG...TLT...................................EASQGHTFNESCIAYVAKGMLEGLM
MkcD         K..................................SNDELWMIMEFL.SGG...TLK...................................NAASNFKFCERKIAYVCREILQGLD
MkcE         K..................................NDEEIWMVMEYM.DGG...TIRDAIS...................................NFTFTEKYVAYITKEILHSLE
MkcF         Y...................................GSDLFIVSEFI.QGG...TLE..................................QASASSHVFKETQVGFIGLELLKAIS
PakA         V...................................ADELWVAMEFM.SGG...CLTEVLDQY................................RDIQLNESQIAFVCQEVLRGLE
PakB         V...................................GDSLWVAMEFM.GGG...CLTEILE................................QFNSVKLVEAQIAYVCAETLKGLA
PakC         V..................................NDRELWVAMEFM.GGG...CLTDILE................................AFDNIKMSEIQIAYVVKETLKALQ
PakD         V...................................INHIWLIMEYM.SYG...SLTDLISNCINIQCN..............GNNQQQQQQQQQQQTYFIESHIAYICQQVLQGMD
PakE         S..................................KLTELWILMDHC.AVG...SINDMMK.................................ICCDTLDEEQIAVVTLNVLNGLG
PakF         T...................................ESHIWILLDFC.ALG...SIRDIIE.................................STEKTLNEAQISFVVKNTLKGLI
PakG         Q...................................GDNLWILMEYC.SAN...SIRDMLE.................................LTEKSLTEKQISVILQQALKGLH
PakH         L...................................KNKYWILMDYC.SLS...SFNDIMQ.................................SIGKTFKEKEISLILQQSLLGLV
Krs1         K...................................ENEVWIVMEHC.GAG...SVCDAMK.................................ITDKTLSEDQIAVVSRDVLQGLA
DDB0216375   K...................................DETCWIIMEYC.AFG...SVSDMMN.................................ITNRVLNEEQIALVCYSTLKGLY
SvkA         K...................................GSKLWIIMEYL.AGG...SVLDLMK...................................PGPFDEGYIAIILRELLKGLE
DDB0216374   K...................................ENNLMIVMEYC.DGG...SILDIMQ.................................MCSITLTEAQIAAILYQIVEGLV
DDB0216379   K...................................SSMLLIVMEYC.KGG...SLLDIIE.................................LCGKRLIEEEIAAVCAGVVKGLV
DDB0216377   K...................................DHQLWIVMEYC.GGG...SVSDLLQ..................................VIDTISEDEIALICREALKGLN
DDB0216378   K...................................GDELFIAMECC.GGG...SITEIYQ.................................ELNIPLNESQIAYVCRETLKGLE
DDB0230010   C...................................KGHVWIILEYC.DGG...TVRDLLQ.........................ATCTPGNPNNLQLFEETLIAYIITELLEGLV
DDB0229972   D...................................NTGFGIVQEFY.ENG...SLSDIYN..................................RSGAFQESLVAKYTLQILEALG
DDB0229973   S...................................GNQLWLFMEFY.PH....SLKDVIT.................................KRTSPFPATEVIWMALEIAKGLE
IfkA-a       QE.................................GQNTLYIFQEYN.STL...TLQQKILS.................................NGIGFEESQVKKFTYQLLLGLL
SAMK-A       D...................................QKNYYIASKYY.PKG...SIK...................................KNTKQSPYSEMNAKRISVKILSGID
SAMK-B       D...................................EHYYYIATPYY.KKG...SIAKIVK.............................SIKSKNNSGFSESNVRNISKKILNAID
SAMK-C       D...................................ENYYYVASKFY.PKG...SIK...................................TKSKSTPYNEINAKKVFGKVLKAID
SAMK-D       D...................................KNYFYIGMKYY.ETF...KFK................................QSNISKFGKNF.EQVIRKISFKILSAID

DDB0230038   D...................................NQTFTTFTEYI.TGS...NLDIVIK.................................NLDEKNELQLILRLSEEIASAMS
DDB0230124   D...................................DNNIYIVHPYY.DAS...NLQTLLFEQSHN......NNNNNNNNNNNNNNNNNNNNNNNNHTIISNEFIHRVSLGISKAMT
DDB0229871b  N...................................TDEYAIISEHVGNNG...SLLTLMQ.................................NHSNQLNWSNTIDLAIQITQSIQ
DDB0229850   VEE...............................PQKQCYCIITEFC.SRD...SLYHIMT.................................DRLIEIGWNRFFQFSMQIILGLQ
ARCK-1       ....................................EPKICLVMEYC.SNG...SIYHSIR...................................KNPPSWERFFSFVQQMLAGIN
```

```
                                                           Subdomain V                                                    Subdomain VIA
SAPKalpha   ..................................EPKLCMVMENC.SRG...SLYHVMD..................................NNSLDIGWERTFRFAIETVRGIE
DDB0229849  ..................................EPKLCMVMEYC.SRD...SLYHVMN..................................TKKYDIGWDRFFQFTMQMTLGVQ
DDB0229848  ..................................KDKLCIVMEYC.SRG...SLYHILK..................................DDTTQFTWDNFFNLGTQAINSLD
Phg2        E.................................ESQLSMVVEYC.SKG...TLHKVLN..................................NPLLDFDWDKWFKWMTEVVEGVR
DDB0229847  ..................................DPKICMIMEYC.SNQ...TLYHLMH...................................LQMNFTWDWVFKFAIEMVRGVN
SplA        AG...............................GEDHHCIVTEWM.GGG...SLRQFLT................................DHFNLLEQNPHIRLKLALDIAKGMN
DPYK4-b     KFD.............................NKEVNHCIVSEWM.SGG...NLSQFIS................................NERKILEINPHLRVKILLDIAKGML
DDB0229872  LD..............................GPSNDRSIIMEYM.EGG...SLRRLLD.................................EKSSYHLPPSLQLSIARDIAEGMN
DDB0229844  ..................................EKKLRMVTEYC.QHG...SLYHIMS..................................KRKMDISWPLVFKWMHQAVDGIN
ZakA-b      KYG.............................EKETNHCIVSEWM.SGG...NLTQFLM................................NNHKVLENNPHLRVKLLTDIAKGIL
7TMK1       .................................ETNLNGIVTEYM.GGG...SLDRLLT................................DRYFLIKQNPILAWNMAISIARGMF
rk1         I.................................PPDICICTEYM.PRG...SLYSILH..................................DQALQLQWSLLIKMMIDAAKGVI
rk2         I.................................SPDICICTEYM.PRG...SLYSILH..................................NEKIKISWSLVKRMMIDAAKGII
rk3         N.................................PPEILIVMEYM.PLG...SLYRILH..................................DPSISLDWPRMKSMALDIAKGMN
DDB0214883  E.................................PSSPCIVTEYL.SRG...SLANILL..................................DESIEMDWGLRLQLGFDCARGMT
Shk1        S................................TPGKLMICTELM.KG....NLESLLL..................................DPMVKLPLITRMRMAKDAALGVL
Shk2        A.................................PEDLSIVTELM.PKG...SVHSLLRA...............................KEDTSDFITFKRAILIARDTVLGMT
Shk3        I.................................PGRCVIVTELV.PKG...NLETLLH..................................DQKIQLPLYLRMRMARDAALGIN
Shk4        Q.................................SGKMQIVTELC.QTD....LEKLLHND................................RTKKEFSLFRRMQMAKDAALGMN
Shk5        K.................................PGKVMIVSELM.QTD....LEKLIHSS.................................EVEPPPLYERMKMCLDAALGIN
DDB0229963  ...................................NPMCIVMEFL.PSG...NLFELIHSKPSE............................QQQSIKLDSTLILAIAFDIARGMQ
DDB0231199  APR.............................SHKSTFMIVTELM.HKG...TLLEVIN...................................KNKPLSLEDIIKYALSVAQGLA
DDB0229940  Q................................NKKYLFHVSELV.KSG...SLRDLLL..................................DKEKPLAYFTQLSIASDIANAMK
DDB0218878e Q................................NKKYLFHVSELV.KSG...SLRDLLL..................................DKEKPLLYFTQLSIASDIANAMK
Rck1        Y.................................DDKYFHLTEYC.HNG...SLFSYLR..................................DQRNNISFGQRLHFALGIARGMR
DDB0231197  K................................NTNKPFIVTELC.SRG...TISNALNQIR..............................KTTGQPPAIPLIVHMAIDAAQSLE
DPYK2       ...................................KPACIITEYM.AGG...SLYNILHNPN..............................SSTPKVKYSFPLVLKMATDMALGLL
DPYK3-b     ...................................DPPCIFTEYL.QGG...SLYDVLH..................................IQKIKLNPLMMYKMIHDLSLGME
Gdt1        .................................SKDYLCLAFEYP.PLG...SLDYIIS..................................KKKLKMSITQKIRILIDVAKGCK
Gdt2        .................................FSGIHGIVTDYC.SKG...CLSKYIH..................................ENKYKITIIQKIRILIDISKACA
Gdt4        N................................FLNTYGIVIDYH.PRG...SLNYYTR..................................NQNIKLSMVQNIRISLDISRACS
Gdt6        .................................DSSHLWIVTEYA.PLG...SLSSLIH..................................QKKEKLSLIQKVKLMLDTAKGCE
Gdt8        N.................................ISHVCIVTEYA.KEG...SLGSLIH..................................DSKIKLSFIQKVKFMLDAAKGFT
Gdt9        S.................................LEHLCIVTEFS.PIG...SLGDLIHGDNNQKKSNTNT......ITNTITNTNTNTNTSTSTSTKLTVKQKLRIAIDIARGCQ
KinX        N.................................NGILFIVTELI.EGG...DLQYYLK..................................NQSIDLPWFLRANIALDVSLAMS
KinY        EPLASPS...................LVNRLLNSSSTWIVTEYI.GGG...NLHERIK..................................DTKKDFPIELRIKLSLDIALAMA
DDB0229853  H.................................EGRYMIVTEYI.KGG...DLHQFIK.................................ARGVSNISWTLRMKLALDIASAFS
DDB0229863  N................................EKDEIHIITEFM.DGG...DLFDALI..................................FGDIPLTWKEKLRISLDIAQSCR
DDB0229866  R.................................GSSLYIVTEFV.QGG...DLAYYLFRNKFDDTPEQYIHRKVNVGSSSTPDLSDPTVHNGEKLVQLAWPLRIKIAYDVACAMA
DDB0229851  K.................................ERSLFLLTELV.SGG...DLHWYIK..................................NKSIDITWKLKVKIARDIAASMA
DDB0229865  K.................................DSLLFLVTELI.NGG...DLGNILL..................................DHKFHIPWSLRVKIAKDIAEGME
GefX        H.................................SSGLYIVTEFV.SGG...DVRQLLK...................................KTPPIGWDKRVSIAVDLAKAMV
DDB0229867  H.................................ESGFYLITEFI.EGL...DLRRYLK..................................SVPKPPKWLSRVNIALGVAKTFL
HH498       E.................................EKELLLVLEFC.DNG...SLYTILNTIPIGGAG.................ANNNNNNNNNNDIIQSLPSINTLSLNIANGMN
GbpC        ...................................QPLCMALEYV.RYG...SLYSLLS..................................NSSIEISWGLRLQIASEIAKGMQ
Pats1       ...................................DPLCIVTEYL.PHG...NLYSFLH..................................KPEMEFSWLFRLKVALDISSGMA
QkgA        N...................................PPRMVMEYV.EHG...DLYHLCQ................................DKERYASLGWTLKLRLMIDIAKGIG
Roco4       N...................................PPRMVMEFV.PCG...DLYHRLL..................................DKAHPIKWSVKLRLMLDIALGIE
Roco5       ...................................DPCCIVMEYI.PNG...TLYSHLR..................................KSFSSITWQLKLKIAINIADAIK
Roco6       ...................................SPFTMVMEYI.DCG...DLHKFLH...............................SPIGDQLNGNWALILKLALDIAKGME
Roco7       ...................................SPFCLVTELL.QYG...DLAKFIR..................................NTAETFSWGTVLKLAIDVAKGMN
Roco8       ..................................FEPYAIVMEYM.DIG...SLSSFLKK...............................KKEDGQVLDWQMLLKIVTDIASGMA
Roco9       E...................................PLAIITEFQ.SGG...NLYDYIH..................................DLKNPLDWQLRIKIAKGIAASLQ
Roco10      ...................................NPIAIITENP.TFG...SKGSTTLSEYIQD...........................RQKHPEIPWNIKLKIALDIARAMD
Roco11      N...................................PPRMVMEFA.PHG...DLYHFLE...................................KKKNIKWSFKVRLMLDIAKGIE
DPYK3-a     D.................................S....IIFESF.KGMN..SLHDLIH..................................RDGLKIDMALFIKISKDIASVMG
DDB0229956  Q.................................PGKMYLVCDYY.SRG...SLYSVIT..................................ANICPLSNARIVHLALQVAKGMN
DDB0229957  K.................................IANWSIVMEYV.PGG...NLMDVLA..................................NPVLVIPYKLVLRMALDIAKGLH
DDB0220138  H.................................ANYCYIITEYL.PRG...SLHDLLH.................................REQLMKLDFKQKVSFAICVALGCY
DDB0220436  ...................................IERIILLEFI.DGN...SLDKYPSQSLPL............................SPSSSLPNPLKVIQDFQQIVDAMI
DDB0230133  S...................................PKCLVLEYI.EGG...SLDKYLH..................................NPNYTFSPQLRLKMANDIAKGMC
DDB0229871a L.................................NGNLAIITEYL.NRG...SLRDVLT.................................TMNKSELSLSVKVKMLIDVAQGMN
DDB0229955  A.................................PPNLCFITEYL.PGG...SLYDALH..................................SKKIKMNMQLYKKLAIQIAQGMN

Bud32       DI.................................GNNRIYMEFI.KGE...TVKHYLY.................................KNQESTQHQNQIESIMKELGNQIG
DDB0231196  Y.................................NQKLFLVMVKE.KCN...LLSFLCNKSEILK............................MQREGI.WTSIFKISKDILKGLV
```

```
                                                       Subdomain V                                                     Subdomain VIA
DDB0229339   Y...................................NEKLFLVMVKE.KCN...LLSFLSNKSEILK............................MQRGGI.WTSIFKISKEILKGLI
DDB0231195   L...................................KFGLSIIIEKC.DM....DLKNFIS....................................CNQINLDLFFILSTQMVNSVS
DDB0229335   L...................................NVGFGIIIERF.DC....SLHDYIQ....................................NNNIDFDLFFELALKMVITIR
DDB0216331   D...............................NNSNMFSLCIVMEYC.EGG...DLMDNLFKRISDSFLKNSG(328)NIESSLNINGNNNNNNCSTLLKLPRKLFYTWIYQICLGVQ
DDB0229344   TQI...............................GDQIEITIMMEFC.EMG...DLLDFLIELSEPQHHHTVT(189)LNIEEHVIHSNEIKKGVDSIYLIEQTQLIEWLLDLSYGVQ
DDB0231281a  SKDE.............................FGLVDRCIFVTEYY.EDG...DLS.......................................SIKEIEFKDIINLFLKMLINLK
DDB0230037a  ILENQ..........................QEKEKIKKQYIITEFY.DGG...DLSSLKIT................................NKKCFSIKNGDIIYLFLKMLIIFK
DDB0219988a  TRDN.............................HGYLQKLTIIIEYY.EGG...DLT.......................................NLKNLNEVLPKDIIYLFFTMLV
DDB0231281b  ...................................ENDIIYILMDYF.DGQ...NLL.....................................EVSQDLINDFTLFSILNQLSKILF
DDB0230037b  E...................................NDKIYAMFPYV.NG....SCN..LS...................................EIDNLNEMDLLNILYQLCFQLH
DDB0219988b  E...................................NNIIYILTKYH.GDY...SNL......................................EEINELNQEDLFEILVQMSDKLK
DDB0231182   AAPTSNLSNVNNNNSNNN(45)TKTITTTTASIKPNIQYMHLILEYA.NMG...TLKEYGK..................................DNNFEFTISQMKSLLFQVIYSLA
IksA         EYDQP.........................ADFGPKVPCLYILMEYA.NNG...NLQDYMA..................................EKRDLIPENEIWSFFIDLCHGIG
IreA         D...................................DEFIYLAISFC.QK....SLDMYVQQTLSLQISP....TDSPSIQSSNNNGNGNNGNNNNNNQIIIDNKTKQMILELFKGLE
IrlA         D...................................QLFVYLGLTLC.EE....SLQDLMESK...........RYKEFIEKTTTTNITTTFNNNIIDENLYEQRILSLFKDVINGIN
IrlB         D...................................KLHVYLGLTLC.DG....SLQNLVE..............................SGKLNDFVISSNKSIIELAKDILFGIQ
IrlC         D...................................ESCVYLGLTLC.DG....SLQNLVEKGDNNLT...................LTQFLGYDINSSSKNNSRLLELIKDIVYGIQ
IrlD         D...................................DMFVYLGLTLC.NG....SLQNLVEKDLEIN..............LISTSNNENNNNNKLKNFIGSELRLLELIKDIVYGIQ
IrlE         D...................................ENCVYLGLTLC.DG....SLQSLYDQS......................KLNEFINQNNNQNNNNNNNRVLDLIIGMINGVI
IrlF         D...................................KHFVYLGLTLC.DV....SLQ.YLV.............................ENGKLNEFINSSGKSLNELAKDIINGVQ
DDB0216373   SNH..............................SQDTKEYRLLLPYY.KDG...TLQDLIDN.........................QRTIYGKDTKKPLFKEKKCLQFFLKVCEAIQ
DDB0229347   NENR.............................NTRETEVLMLMEYC.SGG...SVLDIMN................................ARGEFTRLEEREILAIFSDVCNGVL
DDB0229350   VSD...............................KNNTEMFILMEYC.SGG...HLVEIMQ...............................KRLSSGSKFTDQEILKIFQDICESVA
Nek2         DK.................................PSSRLYIIMEHC.SGG...DLSQLIK...............................KCRNERTYMDEEVIWRTLLQILSALQ
Nek3         L...................................NGCLYIIMEYA.NAG...DIN...............................LEIKKRTLQNKTFSEFEILSWFSQICKALQ
Nek4         IE.................................NNQFLCIIMAYC.ESG...DLFTTLQ.................................KKKNEFISEYQILDWFIQIALGLL
DDB0229345   E...................................NGNYVIIMEFA.ENG...DLFQKIE...............................NQKRSGKPFSDFEIMHYFCQLVIALN
IfkA-b       EKSCDSFQSLEEGNEDLS(263)LSTSSSRKKPPKETHTLYIQMEYC.SKK...TLKTLID...................................NVGGIAEEEAFRLLRQIVEGLN
IfkB         EKSCDSFQSLEEGNEDLS(261)LSTSSSRKKPPKETHTLYIQMEYC.SKK...TLKTLID...................................NVGGIAEEEAFRLLRQIVEGLN
DDB0216407   ESAESLSLTNDNSDDDDD(178)SSSKLKNIKKSTSIPYLYIQMEYC.QK....ILR...................................NLTETGMNLEDDDIWKLFRQIVEGMA
DDB0229432   ELDPKNNKPRSSSFSSVE(256)NNDKNSNSNNLRLKYTVYIQTQYC.EGK...TLRDLLE.................................NPDFKNNSKTTILSLFKQIITGVN
DDB0220611e  ETEELLPPSSSMASTSLS(305)NNKNNIVSPKINRITTLFIVMQLY.SQ....TLSQWLENR.................................PPDQVNEEENLNIFKQICIGLR
DDB0220652   E.EEIITNNSIDHYGEND(70)TNDDNNNYNQLKQKKFSLYIQMELC.KYS...TLRNLINEINNIKSI...........TSIQSTSSIANPIGTNILISLDIKQCREITRQILVALK
PLK          N...................................EENVYILLELC.NQK...TVMDIH...................................KKRKYLMEYETKYYVYQVIMAVQ
Vps15        T...................................DRSGYLIRQYF.HN....NLYDRLS...................................TRPFLSMIEKKFIAFQLLKALE
TBCK         SQ................................KHHDQMLIVSEDY.KN....SLEKQIEIQ...............................AKRQTPIPSNTIGRYAYQILKALS
TtkA         NM.................................AKNYILLVLEFG.EI....DLSKLLQ................................RLQTPNGTNVNFIRIYWQQMLQAVH
DDB0220004   E...................................DSHIFIQMEHC.NGG...NIYKWVT...................................EHIKQSESNLLLLAKQILTGIV
DDB0229384   Q...................................NNTLFIQTELC.ENG...SLQDFLD................................SLSPDQILSEELIWNFLLDVCLGIQ
DDB0216398   E...................................GGHIFIQMELC.ERG...NLKDALN............................LAVQEEGGLGKLPEYMIWQYLCDIANGLS
Scy1         T...................................ETNIYIVTEPI.Q.....LLDELL....................................EDIRNFENAISWGLYQ..EGLS
Scy2         E..................................TKTHIHFATEPI.LA....TLEDLLGYY.............RQRKKSTVDQSSQSEEGYKKKDFTFEELELKAGIFQILDGLL
SLOB1        SG.................................ANDKILVIRTFF.KQG...SLRDQVYKSKPLSPYD...TKYIYKQPKSSSSSSSSSSSSSQSQSVLPIKSIQKYCKQILESLL
SLOB2        IPEKH.................................VAVSVRPFYADRG...SLRDFIHKSKP......................KMPYADKYDTHLQLNEKIVSKFGRQILEALI
DDB0229334   KK.................................SEKNVIIYQESI.NGGI..SID....................................KFDNNNFFSQEVIREICEQILLALR
DDB0231326   D...................................GENYWIVMKSY.KC....SLK................................EWRLKQTLPFLELLPLLLNIFTNVLQSVQ
DDB0231559   DE.................................KNNFLYIFLEYC.PNG...NLFDIIN...................................KEKMKSFDEFFSYAFGVVHCTH
DhkG-a       TSNSNSNNNSNNNNSGG..GGGGGGGGGGGGGTKINNKRRASLTPTS...T...PLS..............................ASPLLFNTKKYADIDINTFLSIAIQLTTILE
DDB0231198   D..................................NPNKRGMLLECA.TAG...SLKTNLLE.....................................YRSWKLVIRFMMDICLGMI
DDB0231179   ....................................LGFSSIIMENM.VDIGYVTLKQLLS........................MDDKQFSALASPFTCRSQMIKPIINAMVIALYS
DDB0231335   .............................NTPSIVGIGINMIVMEKV.DGT...ELE.......................................KYDK.TKLTKKIFMDLVIYLME
7TMK2        VV.............................GSSGQIFEIRLFMEYI.PH....TLDKYLLARSVCGDSNGGG(29)NNNNNNSPLIQPQQQQQYVYPYYFRYSQVVWYLYQISIGLD
DDB0229381   H...................................KTTAIFILEYV.DGG...TLEDFMY..................................SFERGMPLSLISHCLYQSVNAIE
Bub1         K......................................SIMLMDHTIDQG...TLDDVVS.................................STNGVPMEEPLAMFHCIELLKMIE
DDB0230007   ........................................YSFKEFL.NSDQ..YL.............................................SIDTSIIFSKNIMNALY
DDB0229337   .....................................................................................................SGSLPSKLIQSIFKIIL
DDB0229346   LKGCDRM......................LKPRLLSDNDELIFMECE.DMGQYQTLEMIIER..................................RKCKGSLVMTRFIMRGILASAY
LvsG         .......................................................LQYLQRNQKIT........................................TFMIYQLIQLFS
DDB0230125   ................................KQQNQTENW..IQIS.PRG...QLIKNL.....................................EGKPIDISFYTKVFCRNTQ.K
DDB0204911e  N...................................QTENW..IQIS.PRG...QLIKNL.....................................EGKLIDISFYTKSVEILKN.I
CK1          F..................................DGDYNIMVMDLL.GPSLE.DLFNYC.....................................GRKFSLKTVLMLGDQMLRRIE
DDB0216336   H...................................NDYNYMVMELL.GE....NLSELRR.................................KQLDGKFSLGSTLKLGVQMIQSLQ
```

```
                             Subdomain VIB                                                Subdomain VII
Akt1          HLHLS......GVIYRDLKPENLLLT.NEGHI..............................................CMTDFGLCKEG..............................
DDB0220670    FLHEH......GVIYRDLKLENLLMD.LDGNI..............................................CLVDLGLCKEG..............................
DDB0216386    FIHSC......GIIHRDLKVENLIFD.KNGII..............................................KIVDFGFSEVGFTG...........................
DDB0220701    YLHKS......AIVHRDLKPDNMLID.GLGHI..............................................KLTDFGLSKIGIIDDKKMEDSGNTNTNTHFNFSTSPTNTSM
DDB0220700    YLHNH......GIIHRDLKPDNILID.SNGHI..............................................KLADFGLSKFDFVDESCNIKI....................
DDB0185113    YLHSH......GIVHRDVKPDNLLID.KKGHI..............................................KLTDFGLSKVGLLDRQTVVPPSYFSPTLSGKNNQSSSSSSV
DDB0229452    FIHEK......SIIHCDIKPNNMVID.SDGHI..............................................KLLDFGNAKKF..............................
NdrA          SVHTL......GYIHRDIKPDNLLLD.SKGHV..............................................KLCDLGLCTGFHRLHSSEFYQMLVGDAMTIK..........
NdrB          SIHKL......SYIHRDIKPDNLLID.QKGHI..............................................KVSDFGLCTGLQTNRVPTLAEIYKKYEGDNN..........
NdrC          SLHEM......GIVHRDCKPSNFVLD.KLGHI..............................................KLIDFGLSKEGIEKRNGWNKATMNELRKSCLSA........
NdrD          SLHRL......GYIHRDLKPSNFVVD.KLGHL..............................................KLIDFGLSKEGFISRNSKYHTTWKNLREDNTPSNNNNNP..
DDB0231558    SCHRL......GYCHRDLKPENFLIS.RDGHV..............................................KLADFGLSKNVVTRYHLRSASADNTINTTTSTPTTSSNINQ
DDB0216387    AFHSY......GLLHRDVKPENLMIN.KDGHL..............................................VLGDFGSSKLANNSGGGGGNFGNTLTVPMSTSYSTSPSSSI
Pdk1A         HLHSL......GIVHRDLKPENILMS.SDLHV..............................................KITDFGTGKILPPPQSSQQQQQQQQQQQQLPTNSSGNLSSL
Pdk1B         YLHGK......GIAHRDLKPENILLG.KNQHL..............................................KLSDFGSAKQLSI............................
PKA           FLHKQ......NIVYRDLKPENLLID.NQGHI..............................................KITDFGFAKR...............................
pk4           YLHNQ......DIIYRDLKPENLLID.QYGHI..............................................KLTDFGFAKR...............................
DDB0185224    YLHAE......NIIYRDLKPENILID.EKGHI..............................................KLTDFGFSKK...............................
pk2           HLHKQ......DIVYRDLKPENILLD.SEGHI..............................................CITDFGLSKK...............................
DDB0220702    HLHDS......NIVHRDLKPENILLD.SQGHC..............................................ILTDFGLAKLE..............................
pk3           YLHSN......NIIYRDIKPENILLD.EEGHI..............................................KLTDFGLSKSGITSVV.........................
Aurora        YCHSK......HVIHRDIKPENLLIG.VGGEI..............................................KIADFGWSV................................

MLCK-A        YLHGL......NIVHRDLKPENLLLKSKENHL............................................EVAIADFGLSKI...............................
DDB0216385    YLHDK......SVVHRDIKPENILFSDSYGGI..............................................KLGDFGLAKF...............................
DDB0216309    HLHAN......GIAHRDLKPQNLLCAGEEGDD.............................................IRVADFGLSKI...............................
pXi           YLHSK......DIVHCDLKPENLLYS.DNSDQA...........................................IIKLCDFGLSQR...............................
DDB0216308    YMHQH......GVCHRDLKPENLLCSGDDEKEE...........................................IVRIADFGLSKI...............................
DDB0229351    FLHSN......KIVHRDLKPENILFR.DKSLGS...........................................ILKIIDFGLASYVDHDCNCNNNNDNNNNN..............
DDB0216307    YLHSQ......GIAHRDLKPENLLCNGEGEDM............................................TIKIADFGLSKI...............................
DDB0216312    YLHSI......GIVHRDLKPENLLLK.TPTDL............................................TVALSDFGLSKI...............................
Snf1          YCHHH......MVVHRDLKPENLLLD.PINKC.............................................IKIADFGLSNM...............................
DDB0229364    YCHSR......NICHRDLKPENLLLS.GDKRI..............................................KIADFGMGSI...............................
Lkb1          YIHSQ......KILHRDIKPDNILFT.HANVL..............................................KLSDFGVAEDSS.............................
MARK-A        YCHSK......RAVHRDLKPENLLLD.CNRQI..............................................KIIDFGLSNV...............................
MARK-B        YCHSN......MVCHRDLKLENLLVD.EDGNL..............................................KISDFGYSNI...............................
MARK-C        YCHAN......LIVHRDLKPENLLLD.SNGDI..............................................KISDFGLSNN...............................
DDB0231454    HCHKL......NIAHRDIKHKNILFD.NQMNV..............................................KIIDFGLS.................................
DDB0219986    YMHSN......CIIHRDLKLENVLIR.QDGTP..............................................VISDFDLSKDIS.............................
FHAK-A        YLHSK......GIAHRDLKPENILFDSYGDDYL...........................................KIKITDFGLARF...............................
FHAK-B        YLHNL......GIVHRDLKPENILYNEFKQGFS............................................IKITDFGLSSF...............................
FHAK-C        YLHDL......NISHRDLKPENILISAVSHGKS..........................................SVIKVTDFGLAKI...............................
FHAK-D        YLHSN......GIAHRDLKPENILLATPNSFL.............................................LKISDFGLSRA...............................
FHAK-E        YLHNK......GIAHRDLKPENILLKHKNFNQND..........................................AIKLTDFGLSRT...............................
DDB0220010    YLHEQ......KVIHKDLKPENLLVN.SEGVV..............................................KITDFGVSHI...............................
Apg1          FLRQK......QIVHRDLKPQNLLLSDDSEHP............................................ILKIGDFGFAKF...............................
tsunami       YLHYK......KVIHRDIKPQNILIT.SGGQI..............................................KVCDFGFAKTI..............................

Cdk1          YSHGH......RILHRDLKPQNLLID.RQGAL..............................................KLADFGLARAV..............................
Cdk5          FCHDH......RVLHRDLKPQNLLIN.RKGEL..............................................KLADFGLARAF..............................
Cdk7          ACHRN......WVLHRDLKPNNLLMS.INGDL..............................................KLADFGLARQY..............................
Cdk8          YLHSN......WVIHRDLKPSNILVM.GEGKECG..........................................TVKIGDFGLARIFQ.............................
CDK9          YCHRN......NVLHRDIKGSNLLLD.NNGIL..............................................KLADFGLARPFN.............................
Cdk10         YLHSH......WIIHRDLKCSNLLYG.NNGNL..............................................KLADFGLARKF..............................
Cdk11         YMHDN......WVIHRDLKTANLLYT.NKGVL..............................................KIADFGLAREY..............................
DDB0229294    EIHNA......GVMHRDIKAANLLVN.NKGSL..............................................FIGDLGTATS...............................
DDB0229424    ELHSQ......KIMHLDIKPSNLLIN.PRFDL..............................................KIADFGFTT................................
DDB0230050    QCHVN......GIMHRDIKPANLLIT.TNGVL..............................................KLTDFGLSTSY..............................
CK2           FCHSN......GIMHRDVKPSNVMID.HQKRK.............................................LYLIDWGLAEF...............................
DDB0219953    YTHSK......GIIHRDIKPLNIAID.HSKRK.............................................LSLIDWGLAEY...............................
ClkA          SMHKL......SLVHTDLKPENILLE.SSRFTYFDNSIPLQFKNSIDT.............TSNNSVDHYCHLVDTDIVVIDFGGA.................................
DDB0229841    FMHSA......GVIHRDLDPENSIFVDENFNI..............................................KFTEFNNCFLLDTD...........................
DDB0229333    YINSA......GVIHRSLDPKTIAVN.KEYKT..............................................KLFDFSFS..IST............................
DDB0229296    FMHSA......GVIHRDIDPSAITID..QNQC.............................................LKLTEFYFC..FPSNC..........................
Dyrk1         FMSTP....EVDVIHCDLKPENILLRNPKRSA.............................................IKIIDFGSSC................................
Dyrk2         FLSKR......NIIHADLKPENILLKSPTKSGI.............................................KLIDFGSSCF...............................
```

```
                            Subdomain VIB                                                        Subdomain VII
YakA         VLANA......NIIHCDLKPENILLQ.NVNSP..........................................AIKIIDFGSAC................................
Prpf4B       HIKNS......KILHADIKPDNIVVN.EAKNT...........................................IKIVDFGSAG................................
GSK-3        YIHSL......GICHRDIKPQNLLLDTSTSTL............................................KLCDFGSAKI...............................
GlkA         HIHSK......AICHRDITPNNILLS.SKGEL............................................TLADFGSAKI...............................
Erk1         HIHSA......NVLHRDLKPSNLLIN.EDCLL............................................KICDLGLARV.E.............................
Erk2         YMHSA......NVLHRDIKPSNLLLN.SECLV............................................KVADFGLARSITSL...........................
DDB0229430   FMHTN......GFFHRDLKPENIMLV.GER.............................................LKIADFGLARE...............................
Sky1         YIHTKC.....KIIHTDLKPENVLLE.KSFDFFTSNDYIWSEKYGYFKNRTSSS(135)YKFKKNNELFNENHYPRAQLVDLGNAC...............................
DDB0230051   YLHTQ......DLVHRDLKPTSILLF.DDYQI............................................KLCSFGNAISVFTN...........................
DDB0230126   FLHSS......SLVYRDLNPSRVLLT.TTTTTANDDVDCEL.........................QQQQQQQVKVKLTHFGIMRSLQ.............................
Cdc7         HIHQN......NICHRDVKPTNFLYSIKNNSF............................................LLIDFGLAQEMPNSNSNSNSNSNSNSNSNSNSNSNSNSNNN

DDB0229971   YLHQE......GVIHRDIKAANILIN.KAGEA............................................KLSDFGTAGEI..............................
DDB0229896   YLHEQ......GVVHRDIKGANILTT.KEGKI............................................KLADFGVAT................................
DDB0229877   YLHEK......GVIHRDIKSDNILIT.KEGVI............................................KLADFGSCT................................
DDB0231212   YLHRQ......GVIHRDIKAANLLIS.TDGSI............................................KLADFGVATKVS.............................
DDB0229915   YLHSN......KVIHRDIKASNILIT.KEGVI............................................KLADFGVASQI..............................
FNIPK-A      YIHDNE....RKIIHRDLKCDNIMLV.NDNTHSCDVNGNCSRNGGGSAKSANQS(60)GQSSTCRLINCIYSVSKKIKLIDFGLSKG................................
FNIPK-B      SIHEI......GVIHRDLKLENVMFD.KESNK...........................................WKLIDFGLSFSFS.............................
FNIPK-C      HLHNN.....LHIIHGNINTDNVLIADQEFSL............................................KLFDFSLSKDISYYSKDNNNNNNNNNNNNNNNNNNNNNNNN
FNIPK-D      CIHKDN.....KIVHGNIKGENILLV.NDESE................................................YGFSVKF..............................
FNIPK-Ea     SIHEM......GIIHFDIKCQNILIL.YDENEKMLP....................................TDFIKIIGFDHST................................
FNIPK-Eb     KIHES......GVIHRDIKSDHIILA.QDKNNET........................................IVKFIDFGLSKQ...............................
DPYK4-a      FLHKH......QILHRDIKPSNIILD.KNGVL............................................KLIDFGSS.IIDQQQ..........................
ZakA-a       FLHKH......HVIHRDIKTANLVLD.KNKNL............................................KLIDFGASTIQNKKQFEQYLNETTNNNNNPNNNNNNNNNNN
DDB0230002   FLHNNC.....NLIHMDLKCDNIILKPNNYDNNNNNNNNNNNNNNNNNNNSN....INDDNNNSNSNINDDNNFDIKIIDYGLSQKI..............................
DDB0229378   YLNDK......GIMHRDLKPENIMFQ.NQNQNQNQN...................................NKINLKLIDFGLSKAINE............................
Mek1         YLHRKL.....HLIHRDIKPSNILVN.NKGEA............................................KIADFGVSGQL..............................
MEKKalpha    FLHAN......SIIHRDIKGANILID.TKGIV............................................KLSDFGCSKSF..............................
DDB0229911   YFHKT......GRIHRDIKSGNILID.SNGNI............................................QLSDFGVSATL..............................
DDB0230012   YLHSH......QRIHRDLKSDNILIG.EDGAI............................................ELADFGVSAMF..............................
MkcA         YLHGI......QLVHRDLKSQNIMMT.TSGEI............................................KLIDFGLCA................................
MkcB         YLHEN......HMVHRDLKSANIMMS.VEGKV............................................KLIDFGLCE................................
MkcC         YLHAH......NIVHRDIKSGNIMMT.IDGKI............................................KIVDFGLCV................................
MkcD         YLHKQ......NIAHRDLKSANVMVN.DKGEI............................................KLIDFGLCIDF..............................
MkcE         YLASL......NIAHRDLKSSNIMIN.SKAEV............................................KLIDFGFSID...............................
MkcF         YLHEK......KLIHRDIKAANVMVS.TDGEV............................................KLIDFGLCA................................
PakA         YIHKF......NRIHRDIKSDNILIG.ANGEI............................................KLADFGYAAQL..............................
PakB         YIHSQ......HRIHRDIKSDNILLG.SDGSV............................................KLADFGYAAQL..............................
PakC         YIHSL......HRIHRDIKSDNILLG.SEGSV............................................KIADFGYAAQL..............................
PakD         YIHKG......HRTHRDIKSDNILLG.KDGSV............................................KIADFGFVANL..............................
PakE         YLHSK......GIVHLDVKAANILLT.EDKQI............................................KIADFGVSQQL..............................
PakF         YLHSQ......NIIHRDVKAANVLLS.EGCDV............................................KIADFGVSEKL..............................
PakG         YLHQS......NIIHRDIKAANILIN.EDAIV............................................KLADFGVSSQLE.............................
PakH         YLHSK......QIIHRDIKSANILLD.ETGQV............................................KIADFGVSQQ...............................
Krs1         YLHSV......RKIHRDIKAGNILMN.HKGES............................................KLADFGVSGQL..............................
DDB0216375   YLHRN......SKIHRDIKPGNILVS.EEGEC............................................KLADFGVSGQL..............................
SvkA         YLHSE......GKIHRDIKAANVLLS.ASGDV............................................KLADFGVSGQL..............................
DDB0216374   YLHSN......KILHRDIKAGNVLVN.KLGQA............................................KLADFGVSAIL..............................
DDB0216379   YLHSK......RTTHRDIKAGNVLLD.EEGLP............................................KLADFGVSTI...............................
DDB0216377   YLHEF......KKIHRDIKGGNILLN.DRGEV............................................KLADFGVSAQL..............................
DDB0216378   YLHHT......NVIHRDLKGANILLT.ESGDV............................................KLADFGVSGL...............................
DDB0230010   YLRSK......GIIHRDLKSRNILLT.RKGKV............................................KIADFGLATT...............................
DDB0229972   YIHSQ......GIVHRNLKSNNVLLA.KGGKV............................................KISDFAIGIRS..............................
DDB0229973   FLHTQK....NPIIHRDLKPGNIMCS.LDDKG.......................................RVCSIRVCDFDTSKE...............................
IfkA-a       YLHSQ......HIPHRDFKSHNIFFN.QSTSK...........................................MLLSNYG...................................
SAMK-A       YLHSLN....PPIIHRDIKCDNILLD.ENDDP............................................ILIDFGLSYKT..............................
SAMK-B       YLHSSN....PPIVHRDIKGDNILLN.DSDEP............................................ILADFGLSYLA..............................
SAMK-C       YLHSLD....PPIIHRDINSDNILFD.ENDEP............................................ILIDFGLSYKELKDDNNNDDDNYDNH...............
SAMK-D       YLHSLE....PPIIHGNINAKNILLDNENNEP............................................ILIDFGLSYKS..............................

DDB0230038   FLHSF......NIVHRSLHPKNILLN.SDLKI............................................YIKDYGFTSLKDETLKK........................
DDB0230124   YLHSH......DVVHRALKLNNILID.RVSGN...........................................VLVRDYGF.N................................
DDB0229871b  YLHKHQ....PPILHRNITSDCFLLS.SLNNNSNQNNNNNNNNNNNNNNNNNNN(25)TTTTATNITNELNINEIKIKVHDFGLSR.FN............................
DDB0229850   SLHNRK...PKPIVHRDVTSLNILVN.EDWEC............................................KISNFSASR.FNC............................
ARCK-1       ALHQST....PQVLHRDIKTLNFLVN.HNNKV............................................KVADFGLSR.FN.............................
```

```
                            Subdomain VIB                                              Subdomain VII
SAPKalpha    CLHKWD....PPIVHRDLKSLNLLVN.DKWEI.............................................KVCDFGLSR.FN..............................
DDB0229849   CLHNWT....PQIVHRDFKSLNLLVN.EDWEC.............................................KVSDFGLSR.FN..............................
DDB0229848   SLHNWT....PQVLHRDLKSLNLLVT.ENWTV.............................................KICDFGLSR.FD..............................
Phq2         YLHSMN....PPMVHRDLKTLNILIS.SDWTA.............................................KLCDFGLTRTMT..............................
DDB0229847   CLHSWK....PVIVHRDLKSLNLLVS.DNWTI.............................................KVADFGLSR.FA..............................
SplA         YLHGWT....PPILHRDLSSRNILLD.HNIDPKNPVVS................................SRQDIKCKISDFGLSRLK...............................
DPYK4-b      YSHRQ......GIIHRDLTSNNVLLN.FRKKKLLNNNSSNNDE........................QFYDSDEIIAKVCDFGLS..................................
DDB0229872   YLHTNFK..EGPIVHRDLTSSNILLN.SSYTVA............................................KINDFGLSKEM...............................
DDB0229844   SLHTMR....PALVHRDIKSQNLLIN.SQFDL.............................................KVADFGLAKP................................
ZakA-b       YLHKQ......HIIHRDLTSNNVLLD.FKREILPNQLYG................................SNEFTAKVCDFGLS..................................
7TMK1        YLHDWK...PNPILHRDLSTKNILLD.ESLTIA............................................KVADFGLSK.................................
rk1          YLHNST....PVILHRDLKSHNLLVD.ENWKV.............................................KVADFGLST.................................
rk2          YLHGST....PVILHRDLKSHNLLVD.ENWKV.............................................KVADFGLST.................................
rk3          YLHCCD....PIVIHRDLKSHNLLVD.EHYRV.............................................KISDFGLSTRFK..............................
DDB0214883   YLHSRN....PIIIHRDLKTDNLLVD.DSWQV.............................................KVADFGLAT.................................
Shk1         WLHSSN....PVFIHRDLKTSNLLVD.ANLTV.............................................KVCDFGLSQIKQR.............................
Shk2         WLHAS......NILHLDLKPANLLVD.QNWVV.............................................KVADFGLSKYMK..............................
Shk3         WLHESN....PVFVHRDIKSSNLLVD.ENMRV.............................................KICDFGLSALKQ..............................
Shk4         WLHGI.....TRIVHNDLKTANLLVD.INLRV.............................................KVTDFGFSQ.................................
Shk5         WLHGIC.....NIIHRDLKLANLMIS.KDKTV.............................................KIGDFGFSQVIK..............................
DDB0229963   HLHTR......NIIHRDLKSSNLLMD.KHFNI.............................................KIADLGIAR.................................
DDB0231199   YLHSV......DFIHRDIKAANILVD.KNNNA.............................................KVGDFGLSRV................................
DDB0229940   HLHSI......GVIHRDLKSLNVLIT.EDFTA.............................................KVIDFGTSRN................................
DDB0218878e  HLHSI......GVIHRDLKSLNVLIT.EDFTA.............................................KVIDFGTSRN................................
Rck1         YLHSM......SIIHRDLKSMNILLT.KRLKI.............................................KIVDFGTSR.................................
DDB0231197   FIHSK......NIIHRDVKGNNFLVN.ENWEV.............................................KLIDFGVSR.................................
DPYK2        HLHSI......TIVHRDLTSQNILLD.ELGNI.............................................KISDFGLSREKS..............................
DPYK3-b      HLHSI......QMLHRDLTSKNILLD.EFKNI.............................................KIADFGLAT.................................
Gdt1         FLQQS......SIIQKTLRARNIFLYDTNENAEV.........................................CAKVLDLTSSKT................................
Gdt2         YLHRN......GITHRDLKPENILIT.SLNPKSL........................................VCAKVSDFGTSKE................................
Gdt4         FLHKN......GIIHRDLKPDNVLIV.SFDPDSS........................................ICAKISDFGTCRE................................
Gdt6         FLHSN......DITNCNIKSNNLLLFSTMEKDDV.........................................CVKISDFGIKKF................................
Gdt8         FLHAS......EIVHGDIKPDNLLVF.SKE.ISG........................................VCVKICDFGNSEE................................
Gdt9         FLHQC......GIIHRDLKPDNVLVF.DINHNAP........................................VCAKISDFGTSKET...............................
KinX         YLHSK......SIVHRDLKSTNLLVD.KNWKI.............................................KVCDFGFARIV...............................
KinY         YLHSR......DIIFRDLKSKNILID.DSSSP..........................................IRGKVCDFGFARILN..............................
DDB0229853   YLHSK......KVIFRDLKAKNILVD.EIGDG.........................................LYRAKVIDFGFARIF...............................
DDB0229863   FLHAR......GILHRDLKSQNILLS.TNRRA.............................................KLCDLGLARMF...............................
DDB0229866   YLHSR......NVIHRDLKSTNLLVG.DNWRI.............................................KVCDMGFARTAQV.............................
DDB0229851   YLHEN......GVIHRDLKSTNLLVA.ENWVI.............................................KVCDMGLARKM...............................
DDB0229865   YLHSK......QIMHRDLKSNNLLLG.RNWTI.............................................KICDFGFAKEI...............................
GefX         FLHAK......KIIHRDLKSKNILLD.EFQRI.............................................RLCDFGFAR.MS..............................
DDB0229867   FLHSK......NLLHRDLKSKNILLDISRNQI.............................................KLCDFGFARVGSQYSNGSDSSSSEDESDSEC...........
HH498        YLHSLK....PQIIHRDLTSQNILID.RNGIA.............................................KIADFGISRFK...............................
GbpC         HLHSHN....PPVIHRDLKSPNILLNGITEGQNS.........................................VATIIDFGTST.................................
Pats1        FLHSST....PPIIHRDLKSPNILLA.SINENAQ........................................TIAKVVDFGLS..................................
QkgA         YMQNQN....PPIVHRDLRSPNIFLK.SLDENSP........................................VCAKIADFGLS..................................
Roco4        YMQNQN....PPIVHRDLRSPNIFLQ.SLDENAP........................................VCAKVADFGLS..................................
Roco5        HMHGFT....PKICHRDLKSPNILML.SDMNAAV.........................................VCKVSDFGETRA................................
Roco6        FLHSVT....PPLLHRDLKSPNVLL..SMKDGVY.........................................TAKVGDFGLSSRMFI.............................
Roco7        FLHSCK....PMIVHRDLKSANILLGGSSMDNLVA.........................................KVGDFGLSI.................................
Roco8        FLHNIT....PPLVHRDLKSPNILLA.SHPTNPNE.......................................ISAKVSDFGLSR.................................
Roco9        TLHDSR....PSVVHRDLKSPNILLS.SKDSLTM.........................................ECHLCDFSLS..................................
Roco10       KLNSHS....PPFLLTNLTSSGIILE.KSNDHQSSGNGLE..............................IEPFVNAKIIDFSQS..................................
Roco11       YLQNQN....PPIVHRDLRSPNIFLF.SLDENAP........................................VCAKVADFGLS..................................
DPYK3-a      LLHSK......DVAHGNLTSRSIYLD.RFQI.............................................VKVSF......................................
DDB0229956   YLHSL......GIIHRDLKPGNLLID.QDWNI.............................................RISDFGLSR.................................
DDB0229957   YLHSL......GILHLDMKSPNLLVS.SLSTSAK........................................VNIKVADFNTCINRSRITAGFF.......................
DDB0220138   HLSTYEP....PIYHTDLKTKNLLVT.NALKI.............................................KIADFGLASFAKKSLTTIINNNNNTNNTAT............
DDB0220436   YLHNEI.....GIIHFDLKPSNILKN.SKNNK............................................LKLIDFGISKFLNN.............................
DDB0230133   FLHRN......EILHLDLKPQNFLVV.SISPEAP........................................VSIKLADFGLA..................................
DDB0229871a  YLHTYS....PPIIHRDLKSLNLLVD.NNFNV.............................................KVSDFGLSRFIS..............................
DDB0229955   YLHLS......GVIHRDIKSLNLLLD.EHMNV.............................................KICDFGLSKL................................

Bud32        IIHEM......NVIHGDLTTSNMLLR.ESTNE............................................LVFIDFGLSYT................................
DDB0231196   SLREA......GMYHRDFKTANFLVS.NTGKI.............................................LISDFGTSR.................................
```

```
                            Subdomain VIB                                 Subdomain VII
DDB0229339   SLREV......GMYHRDFKTANFLVS.NTGKI.............................................LISDFGTSR................................
DDB0231195   QVHNHYPEAEINVYHRDIKLENWLIK.TIDGNHT...........................................VFISDFGLSR..SN...........................
DDB0229335   NLHKCH...LNEIFHRDIKPHNWLVKKTKDELV............................................VVLSDFGLSRENS............................
DDB0216331   SIHKN......HLVHLDLKSENIFLS.ESQKI.............................................KIGDFGLAK................................
DDB0229344   ALHRA......SMIHRDLKSENIFIS.GSNKL.............................................KIGDFGLAIQ.S.............................
DDB0231281a  EFQN.......VIIHRDIKPENIFLR.KFKNNDGK.....................................VELDCYLGDYGSAQ................................
DDB0230037a  EFQT.......VIIHRDIKPDGSIVN..DYYL...............................................CDLGSST................................
DDB0219988a  ILKEFKN....SIIHRDIKPENIFFVKNGPDL...........................................EFYLGDMGSSS................................
DDB0231281b  ELETV.....YSIHHRDLKPDNILIK.NDLTI.............................................CLADFGIS.HFSK............................
DDB0230037b  QLENL......GIFHRDVKPENIISL.RYGNGGIV.........................................VFIVDFGISQYK..............................
DDB0219988b  ILESL......QIYHRDIKPENILFK.REIVNGNI......................................QSKVCLIDFSIS.................................
DDB0231182   ISQKE.....FEFVHNDLHFGNVLLT.SFPVDKKYIVYQDKLDNG...................EFNNWIVGGDFIVKISDFGLSRIK..............................
IksA         YLHHS......GIIHRDIKPPNILIHQSYDSITDR.....................................EVTHLMISDFGTCDTIGPLES.........................
IreA         HLHSL......NIVHRDIKPHNVLID.PNNRV.............................................KISDMGLGKL...............................
IrlA         FLHCQ......DIVHNDLNPRNILVH.KGNF..............................................VISDLGLSKMQ..............................
IrlB         FLHSH......DIVHNDLNPRNILTL.IGKTSNNNNSS...................................NNSFIISDLGLSKME..............................
IrlC         FLHQQ......GIVHNDLNPRNILIK.DDRFI..............................................ISDLGLSKME..............................
IrlD         FLHQQ......GIVHNDLNPRNILIK.DDRFI..............................................ISDLGLSKME..............................
IrlE         FLHDQ......NIVHNDLNPRNILVK.DNRLI..............................................ISDLGLSKMN..............................
IrlF         FLHSH......DIVHNDLNPRNILTL.STNKNNNNNNKSNNKIKSN................NNSNNNSNNNSNNSFIISDLGLSKME..............................
DDB0216373   VFHNHS....PPLAHRDIKPGNIVLQ.NLRRPSNNNNNNNNNNNNNNNNNNNNN(17)NNNNNNNNNSEDSDIEAIPILMDFGSVREARIKIE.........................
DDB0229347   AMHQQQ....PPIAHRDLKIENVLYC.EHSNR............................................YKLCDFGSSTIKTFNT..........................
DDB0229350   YMHSQQ....PLIIHRDLKVENVLLD.EESGI............................................YKLCDFGSATEEITRM..........................
Nek2         EIHNRK...DGVILHRDIKPGNLFLD.ENKNI.............................................KLGDFGLAKI...............................
Nek3         YISSR......NILHRDLKTQNIFLS.IVNGD..........................................YFIKLGDFGIAKIL..............................
Nek4         YMHKK......KVIHRDLKTQNIFLT.KKNII.............................................KIGDFGISRV...............................
DDB0229345   YIHSQ......NIIHRDIKPKNIVLS.SSDSGSGSGSGSGS............................SNDSIPLLKIADFGVSKLM..............................
IfkA-b       HIHSQ......QIIHRDLKPANIFID.NEQNV.............................................KIGDFGLATSGAPVSKSDDLNSSTSNTANN...........
IfkB         HIHSQ......QIIHRDLKPANIFID.NEQNV.............................................KIGDFGLATSGAPVSKSDDLNSSTSNAANN...........
DDB0216407   YVHGQ......GIIHRDLKPSNIFFD.SCGDI.............................................KIGDFGLAINNKTTSTLSPTTNINSSTSSAGSLTTSSNSVQ
DDB0229432   YIHSM......GMIHRDLKPANLFLS.S.GVI.............................................KIGDFGLVKDITATTPSTTPLTNNTPSTTPIDTTPTSSANT
DDB0220611e  YIHSK......GIIHRDLKPGNVFLV.REEDHHQQSSFSNISSS.....................FEENSDGGGLLVSLGDFGLAVQHTIVSSTPSPTPTSTPSLTPVNSHISPIDQI
DDB0220652   YIHSQ......GFVHRDITPDNVFVCQSPFGI.............................................KIGDFGLATTIESLTVDSNN.....................
PLK          YLHNN......NIIHRDLKLGNLFID..NMRI.............................................KLGDFGLSTK...............................
Vps15        QSSFK......GVFHGDIKSENVLVT.TSNWV.............................................YLSDFACYKPT..............................
TBCK         YLHSQ......DLTHRSLSLDNIKLD.DNNQI.............................................KLTNYGLYY................................
TtkA         TIHEE......KIIHGDLKPANFVSV..QGSL.............................................KLIDFGIAKAIQS............................
DDB0220004   YIHSL......GLVHLDIKPENIYII.YKCNQNQIITNNNNTCSINNSSNGSDS(43)QNNYLIIDGNKINFNSITFKIGDLGL..................................
DDB0229384   HIHSY......NMLHLDIKPENLFIS.SQGNI.............................................KIGDFGMAVKLETTNNNNNGNGGCQS...............
DDB0216398   HVHEK......GIMHLDIKPENLLFS.NDGVL.............................................KIGDFGVCS................................
Scy1         FLNNKC.....NLTHGNIQSSSIFVN.KGGDW.............................................QIGGLDFVSDVKD............................
Scy2         FLNQTA.....KLLHRNISPESIFIT.KDLKW.............................................KLGGLGFTCSIETKEPPISNLSLQDL...............
SLOB1        YLKSK......GIQFSHLHLSNILINQSNDTC.............................................QLVDIENCL................................
SLOB2        FLKNH......NFPYFHLNSANVLVD..DQIC.............................................LISDYENSF................................
DDB0229334   FMDNH......QIVHRDLNCSNVIIFPETLTI.............................................KIIDFGVTKT...............................
DDB0231326   FLGDH......KINHFDIKCDNFLVHPLKKGTIEEDFWNQP............................TNDPNFAVCLADWGEAKVY..............................
DDB0231559   DIHSNP...KGALIHRDIKASNFLVD.KNNLV.............................................KIGDFGTARFD..............................
DhkG-a       SIHGK......GCIHRDIRPNNIYIN.SECKV.............................................KLANFQFSIFKKTSSFLKKSKFQQQHQQSENNNNNTLDSDI
DDB0231198   DIHKC......QIIHRDLKPDNILVF.ENFNSSEASDENDE..........................RQREFGGLICKITDLGASIQKE.............................
DDB0231179   VNIEK......NYTHFDLNHGNIFVNIHTLDTCQSISGSGSGS......................GSGSGSNTRNPVVLGDFGCARKM..............................
DDB0231335   MNIF.......EIQHRDLHAANILIK.EDGSL.............................................SIIDYGY..................................
7TMK2        NLHAN......KIAHRDLKSNNIFVTLSESEVK..........................................ICKIGDFDISRSFNNPKVLNVLS....................
DDB0229381   YMNSK......KCSHRDIKPANILML.RNRKPKLKQQSEQQQQEDGGYIRYSGQFQLEPSSQENNNYQFDPDELPILKVTDYGYAS.IS.............................
Bub1         DLHLV......GIIHGNICANNLHFL.FGQTSDWPDWTAG..........................ECKGAWKSKGFTLTDFSRSIDTT.............................
DDB0230007   YLHSN......NIIYADLKPSNILID.SKGIFKLNSINNSILIENNNNNNNNNN...NNNNNNNNNNNNNQQIMITSRQQIFNLYNNNIDNDNNNNINE...................
DDB0229337   SNYDY......NAIGKHFKTESILIG.ESLF.........................................KWPSPRIQQFD...................................
DDB0229346   YFESH......QVIHQTLHPGNIYIL.EDSLG......................................................................................
LvsG         FLHQR......EIVHGDLQPSNIHLN.NQMW.......................................................................................
DDB0230125   H.TSR......EVIHRDIRLSNLLMD.SGGDP.............................................LLVDFGFANF...............................
DDB0204911e  H..QR......EVIHRDIRLSNLLMD.SGGDP.............................................LLVDFGFANF...............................
CK1          FIHSN......NFIHRDIKPDNFLMGIGKRGHV...........................................VNLIDFGLAKRYRDPK..........................
DDB0216336   AVHDL......GYLHRDVKPSNFAIGLNPSKRN..........................................ITYLIDFGLARRFVLA...........................
```

| Sequence | Subdomain VIII | Subdomain IX |
|---|---|---|
| Akt1 | LLTPTDKTGTFCGTPEYLAPEVLQ | GNGYGKQVDWWSFGSLLYEMLTGL |
| DDB0220670 | IQEGHVTYTMCGSPEYVAPEIIN | GTGYSKSVDWWALGTLFYEMIAGL |
| DDB0216386 | NSIKNGNKKNNKCLGTPYYMSPEVIN | KRGYGKTIDWWALGIIIFELLSGE |
| DDB0220701 | MDDS.........STTGNPNGNGNTSLNSSQTNILSPYPQRKNTLKTPLKKPVKKVVGTPDYLSPEILL | GTGHGQTVDWWALGIILYEFLTGS |
| DDB0220700 | LKNSFTLYNTNNNNTTENQKILGTPYYIPPEVIL | GKGYGKTIDWWSLGIILYEFLIGY |
| DDB0185113 | SNIGGSNTI(98)SNIPTTTTTTTTTTTGQQSQQQSQQQQQTTPPLPPHNIHRKLSCVGTPDYLAPEILL | GIGHGASADWFSLGVILYEFLCGV |
| DDB0229452 | NQKKPTSNDGILGSPRYISPEVLL | FEPQSPAVDYWSLGIVMFELITGT |
| NdrA | MKLIEATPLTQTERIASWKKARRALAYSAVGTPDYTAPEVFL | QIGYNKEVDWWSLGVILYEMVVGH |
| NdrB | IREEDQTPQSRSARFDSWKRQRRVLAYSNVGTPDYTAPEVLM | KDGYSAECDWWSVGVIMFEMLVGY |
| NdrC | SFTSNTMKAASAYLSGGTNVMAYRRPIAHSAVGSPEYMSPEIVN | DQGYDMTCDYWSLGCVFVEMLCGF |
| NdrD | NNNSLKSSTSLKNSSDCVGNIHGNNFKQ.PLNKKV.TFSKVGSPEYMAPEMLA | GKGYDISYDYWSLGVVFFEMLIGD |
| DDB0231558 | LSS.........SMIEHTPMKFGAAANGNGPLNSSVTDFGSFKD.LSVAARLAYSVVGSPFYMAPEVLQ | ATRGYGDEVDWWSLGCMFYEFIFGV |
| DDB0216387 | SSSLLGGSGGFTPSHWNGMAGSAGSSFGSSSLGRSFDNTPPNFNSFLRNPHSSYTSYIGTPQYMAVEVVQ | GVNYSKLCDFWSLGAILFELVTGQ |
| Pdk1A | LNNVNNLSV(48)NNNNTAAGSNTNTNTNTNINTNINANINNIKTTEIPKLTRNNSFVGTAEYVSPELIS | NKETSTDSDLWALGCIIYQMASGR |
| Pdk1B | GSHHKGSRSGSFCGTAEYVCPELLT | EKSAGVEADIWSYGCLLYQLVSGK |
| PKA | VEDRTFTLCGTPEYLAPEIIQ | SKGHGKAVDWWALGILIFEMLAGY |
| pk4 | ITENTKSMCGTPEYIAPEILS | GHGHGKSADWWSLGILIYEMLVGV |
| DDB0185224 | TVGGKNTSSVCGTFDYMAPEILN | SSNGHGKPVDWWALGVVVYELVTGK |
| pk2 | IETTDGTFTFCGTPEYLAPEVLN | GHGHGCAVDWWSLGTLLYEMLTGL |
| DDB0220702 | VKTNNETFSFAGTLEYMAPEMIQ | HATCGKAVDWWSIGILMYDMMIGK |
| pk3 | GSKNGGEGGFATTFCGTPEYLAPEIIT | GAGHGKAADWWSVGILLFEMLTGR |
| Aurora | HAPNTKRSTFCGTLEYLPPEVIE | KKGYDQTADVWSLGILIFEFLVGR |
| MLCK-A | IGQTLVMQTACGTPSYVAPEVLN | ATGYDKEVDMWSIGVITYILLCGF |
| DDB0216385 | YEESIGLELACGTLAYSAPEITN | NQVYRKSVDMWSCGCILYFILFGR |
| DDB0216309 | FGDGDYLETCCGSPEYVAPEVLE | CKPYDKACDLWSVGVITYVLLTGC |
| pXi | CGSGSPLRSLVGTLTYMAPEISS | CTGYGKPVDLWSIGVISYILLCGF |
| DDB0216308 | FEGGEELKTACGTPDYVAPEILE | CKPYDTSVDMWSIGVITYILLCGF |
| DDB0229351 | NNNNNNNTNGEVIQPSSPPPTKLIDVCGTPEFQAPEMVK | RLGYSYPVDIWSTGIILYILLCGH |
| DDB0216307 | FGTGEALETSCGTPDYVAPEVLT | GGSYDNAVDMWSIGVITYILLCGF |
| DDB0216312 | VGDDVFMKTTCGTPSYVAPEVLNNI | SNSPTAYSDAVDMWGVGVITYILLCGF |
| Snf1 | MQDGDFLKTSCGSPNYAAPEVIS | GKLYAGPEVDVWSCGVILYAFLCAK |
| DDB0229364 | VRKDMLLHTSCGSPHYASPEVVS | GIDYDGQKADVWSCGVILYALLTGK |
| Lkb1 | QLEDFECLSRSYGSPAFQPPELTQ | FQTTFSPFKIDIWAMGVTLYLMTIGK |
| MARK-A | FTPGSYLKTFCGSPTYASPELIL | RKEYNGPSVDVWSMGVVLFVLVTGY |
| MARK-B | IKPGNLLSTFCGSPVYAPPEILL | EKRYNGNEVDIWSMGVILYAMVTGQ |
| MARK-C | IQPGKLLESFCGSPLYAAPEILK | AEKYLGPPVDIWSLGVIMYAVLCGN |
| DDB0231454 | NWSYQEKMSFCGTPAYAAPEMLL | GINYNGPEVDIWSLGVILYSLVTGK |
| DDB0219986 | ANVNVTVFNINGGTELYKAPEMKE | QNVKGSYSTDIWAFGVMLYKCIFQFVNNNNNGNNGKN |
| FHAK-A | IHEGELAKTLCGSPLYVAPEVILSLHHK(16) | DINSVGYGKSCDAWSLGAILYIVLCGT |
| FHAK-B | VEESQYLQTFCGTPLFFAPEVIAN | NFFSNGYGKSCDLWSIGVTLYLSLCKY |
| FHAK-C | IGEKEMATTLCGTPLYVAPEIIRNCLHG. | DGGAQVNTGYGKEVDVWSLGCILYILLSGR |
| FHAK-D | MDEGTYMKTMCGTPQYVAPEILTK | GEREGYGKSVDLWSIGVITYILLCGF |
| FHAK-E | VSDGSFMKTMCGTPQYLAPEILTS | SGGHNGYGLEVDCWSMGAILYIMLCGY |
| DDB0220010 | FDDDDVVRCSRGSPAFLAPELCRN | ESQPISGKGVDVWALGVSLYCLIFAR |
| Apg1 | IDPFSLSDTFCGSPLYMAPEILH | RKNYTVKADLWSVGIILYEMLVGE |
| tsunami | SSNSILLTSLKGTPLYLAPEIIQ | EQPYDYKADLWSLGIILYQILVGS |
| Cdk1 | SIPVRVYTHEIVTLWYRAPEVLL | GSKSYSVPVDMWSVGCIFGEMLNKK |
| Cdk5 | GIPVRTYSHEVVTLWYRAPDVLM | GSRKYSTPIDIWSAGCIFAEMASGR |
| Cdk7 | GSPNKVFSPQAVTIFYRAPELLF | GAKSYGPSVDIWSIGCIFAELMLRT |
| Cdk8 | SPLKPLNENGVVVTIWYRSPELLL | GSKHYTRAVDIWAIGCIFAELITTK |
| CDK9 | SSEKKQILTNRVITLWYRPPELLL | GTFHYGPEIDMWSVGCIMAELLSKK |
| Cdk10 | GYPIESITPCMVTLWYRSPELLL | GCQKYSTAVDLWSIGSIFGELLIGR |
| Cdk11 | GSPLKPLSKGVVTLWYRAPELLL | DTEIYTPAIDIWSVGCIFAEIISKE |
| DDB0229294 | YTKRSVFSSKVVTLWYRAPELLL | GSTQYGPEIDMWSIGCVLIELVTSR |
| DDB0229424 | YIGNPHLAHQVISLFYRPPELLM | GSRNYGPEVDIWSVGCIIVEMLTGF |
| DDB0230050 | SKRSEKFLSSNVVSLYYRPPELLL | GSCIYGPEIDMWSVGCILMEMINNS |
| CK2 | YHPNQDYNVRVASRPYKGPELLV | DMEDYDYSLDMWSLGCMFAGMLFQK |
| DDB0219953 | YHPGKNYNVRVASRHYKPPELLV | NMFDYDYSLDMWSLGCLFAGLILDR |
| ClkA | TFENTHHTAIVCSRPYRPPEIIL | GMGWSYPCDIWGVGCILVELYLGY |
| DDB0229841 | PT.FLFNKEYITNTYSYRAPETIW | GDSLYTEATDVWGAGVLFAELLLGK |
| DDB0229333 | DYPTDIFF.KDYEETPWPYRSPETII | MLKEFSFECDVWSIGVIFAELILGE |
| DDB0229296 | PVDLFF.NDYDTSSFIYRAPETIW | RNTTYTTAIDVWNIGVIFGEMILGK |
| Dyrk1 | HSNERMYKYIQSRFYRSPEILL | ELEYSFSIDMWSLGCILVEMHVGE |
| Dyrk2 | ENEQIFTYIQSRFYRSPEVIL | GTKYDKSIDIWSLGCILVEIFTGV |

```
                                                                    Subdomain VIII                    Subdomain IX
YakA          ................................................YEKSTLYTYIQSRHYRSPEVLV............GTVYCASIDMWSLGCISAELFLGL............
Prpf4B        ...............................................EIHESEITPYLVSRFYRAPEIIL............GHKYDYSIDVWSVGCCLAEFFTGK............
GSK-3         ...............................................LIKGETNVSYICSRHYRAPELIF...........GSTNYTTTIDVWSLGCVLAELLLGQ............
GlkA          ...............................................LESNHTSMSYICSRYYRAPELLV...........GCSNYTTKIDIWSIGCILAEMLIGK............
Erk1          ..............................................DATHQGFMTEYVATRWYRAPEVIL...........SWNKYTKAIDIWSVGCIFAELLGRK............
Erk2          ............................................ESIAEANPVLTEYVATRWYRAPEILL...........GSTKYTKGVDMWSIGCILGELLGEK............
DDB0229430    ...............................................IESKPPFTDYISTRWYRAPEVLL...........RCTYYNAPIDIWAVGAIMAELYSLK............
Sky1          ...............................................WTDKHFTDDIQ.TRQYRAPEAIV............KAKWGTPVDIWSAACMAFELATGD............
DDB0230051    .............................................FGNSITQPTYSNPSSYSYLSPEYICSVLDKENHSKLSNEIDWKAFDMWSIGCIFLELIYKK............
DDB0230126    ................................................GNVDSEPAEDG.MIYGSPEI.............FGRVINTESDIWSLGVIFIYLLQTKQE.......RTK
Cdc7          NNNNNNNTN(64)PSTNENGTTTSNASSTSNTTSSSSSSSNKSKNLRNDPKPQPAPRAGTRGFRAPEVLL...........KYNKQTTAIDIWSVGVILLCMISGR............

DDB0229971    ...............................................IKESDKRYSVVGTPYWMAPEVIE............ISGHCQVSDIWSLGCTIIELFTSY............
DDB0229896    ...............................................KFDDTSAAAVVGTPYWMAPEIIE............LNGATTKSDIWSVGCTVIELLTGS............
DDB0229877    ...............................................YSAIDRKLTVVGTPFWMAPEVIQ..........MDMNARSTACDIWSLGCTLLEMLTGN............
DDB0231212    ..............................................DLSSDNPDDTFAGTPYWMAPEVIQ............MQGISTACDVWSLGCTIIELLTGT............
DDB0229915    ...............................................DSESQLRFSVVGTPYWMAPESIE............ISGCSSASDIWSLGSTMIELLTGN............
FNIPK-A       ................................................CDGDSKYYSLVGTSTHMPPEVALR..........NNTACRKSDIWSLGCTIIEMAGGN............
FNIPK-B       ..............................................PSNDGSKCYTQCGSIGYIPPEIKL............GGQCGRKSDIWIFGCLVIKMLGGE............
FNIPK-C       NNNNNNANN(28)GGGGGGEASITSTISTILSGSSSSQLSTPSTSLESSFSLEINLNDSSCYLIAPEIRN.......QQKITSKGLSKKSDIWGLGCLLLMVGG..............
FNIPK-D       ...............................................INFS................LNI............GYEDSIHLDMFNLGCVLIQMLG..............
FNIPK-Ea      ...............................................LDSEVNSNIIGVTETHMAPEIKLK...........NGKLGYKSDIWSLGCTLIEIVGGN............
FNIPK-Eb      ...............................................IEKNSKYYSFVGTDSHMAPEVKLQ...........NGKAGSKSDIFCIGCTMIEMAG.L............
DPYK4-a       ...........................................DDGDNICKESSFAITGTHTYMAPEVKK............LHRSTKKSDVWSLGCTVLEIVGG.............
ZakA-a        NNN..........NNNNNNNNNNNNINNINNNNDLNGSGSGISTYLNEQYKQSSFAIIGTFNYMAPEVKR............NYRATRKSDVWSLGCTIIEMAGG.............
DDB0230002    ...............................................GDLKRDHYG.....TYLPDECIL..........NNVSIVNYTFDVYSLGFISFELFYPTQF..........
DDB0229378    ...........................................NIINKTVKLQTISSVGTTLYMAPEILL............NNKGSNSSLDIWSLGCIIVEMKWG............
Mek1          ...............................................QHTLSKAVTWVGTVTYMSPERIS............GRSYSFDSEIWSLGLTILECAIGK............
MEKKalpha     ...............................................SGIVSQFKSMQGTPYWMAPEVIK............QTGHGRSSDIWSLGCVIVEMATAQ............
DDB0229911    ..............................................IDTGETSRNTFVGTPCWMAPEIME............QVNYDYAVDIWSFGITALELARGK............
DDB0230012    ..............................................EKNTCCSRKTIVGTPCWMAPEIIS............ERGYNQGVDIWSFGITAIELIRGK............
MkcA          ...............................................NLSKGERIRMCGSPIWMPPEMIQ............QKQHGYTCDIWSTAICLLELANRN............
MkcB          ...............................................DVATSTPMHMVGSPFWMAPEMIQ............QKYHSTPVDIWSFAISLLEMANQR............
MkcC          ...............................................DANERKLVHMAGSPFWMSPEMIR............GESYGCPTDIWSFAICLLELANGE............
MkcD          ............................................... SIEKEEINMLGSPSYISPEMIN............GNPHSLSTDIWSFGICALEMLLGK............
MkcE          ...............................................LTHLKQDINMCGSPFYMSPEQIQ............DKAHGLAVDIWSLGIVVAEMVRGR............
MkcF          ...............................................SVEKGGSQHMVGSPYYMSPEMIR............GEECSYPSDIWSFGICILELLFKK............
PakA          ...............................................TQIRQERNSVVGTPYWMAPELIR............GNNYDFKVDVWSLGIMTREMAEGE............
PakB          ...............................................TKSKQKRVTIVGTPYWMAPELIR............GQNYDRKVDIWSLGIMAMEMAESE............
PakC          ...............................................TQKQQKRNTVVGTPYWMAPELIR............GHDYGVKVDIWSLGIMMMEMAEGE............
PakD          ...............................................TRKKLQRNSVVGTPYFMAPELIR............GNQYNHKVDIWSLGILARELSEGE............
PakE          ...............................................QTEYGQANVYIGSPLYMAPEVIL............KAPYNSKADIWSLGITLIELAEGR............
PakF          ...............................................NGALDQSKEMIGTPLWMAPEVIL............KKNYDYKADIWSLGITIIEMADGL............
PakG          ...............................................DSLRGEASQLVGTPLWMAPEIIK............RQNYNNKCDIWSLGITAIEMAESF............
PakH          ...............................................IQSTFSKGSIAGTPYWMAPEILN............QTDYNNKIDVWSLGIVAIELADGE............
Krs1          ...............................................SDTMAKRQTVIGTPFWMAPEVIQ............EIGYDYKADIWSYGITCIEMAESK............
DDB0216375    ...............................................SERTRKRNTVIGTPFFLAPEVIQ............EVGYDNKADIWALGISAIEMAEFH............
SvkA          ...............................................TDQMTKRNTFVGTPFWMAPEVIK............QTGYDSKADIWSMGITALEMAKGE............
DDB0216374    ...............................................VNTGFKQKTVVGSPYWMSPEVISP........PKGSNGYDSKADIWSLGITAIEMAESK............
DDB0216379    ...............................................AEQGQKMNTVIGSPYWMAPEIIM............GQGYDQKADIWSLGITAIEIAELV............
DDB0216377    ...............................................FNTFSKRNTFVGTPYWMAPEVIQ............ENKYDGKADVWSLGITAIEMAEGL............
DDB0216378    ...............................................LDKSSKRNTFIGTPYWMAPEVIEN........RSNPVPYDTKADIWSLGITLIELAEAE............
DDB0230010    ...............................................CSLGRGRTRMCGTMGRIAPEVIR............REPYDTQSDIYSLGCLIIEMAEGT............
DDB0229972    ..............................................SAIEPSIRFSLNGYPFWTSPEVLS............MKPFNHKVDSWSIGCLIMELIVGH............
DDB0229973    ...............................................LGNGVTLKTCIGTPCYMAAEVLNVA.......DGGNSGYSLKADIWSFAMLCFEIISLL............
IfkA-a        ..................................................GRNIKIFDHFEKLNSLKNLNSWISS(28)NQINNEIDRREDIINLGIVVLEMLCGSDLAN(22)FIQP
SAMK-A        ...............................................IDDSTNLKTLCKKPFWASPDVNN..........QEIQIFSEKTDIYSFGCTIFEMIVGW............
SAMK-B        ...............................................IENNTNFKTACCSPFWAAPEILN..........KSTSDFSRKCDIYSFGCTILEMIVGS............
SAMK-C        ..............................NHNHNHNHNHNHDNDNDNDTNVKIKTQCMEPRWPSPEIHR...........EPPHFSKESDIFSFGCTLFEILG..............
SAMK-D        ..............................................IDLLTNQKTQFISPCFITPEYFYK..........KTKNKISKEADIFSFGSTISNMIKGG............

DDB0230038    ..........................................KFMSFQLKNQLLHTQ.....YLAPELFNVL.......SGSKGGYDTKVDVFSFGVLLWEMFA..............
DDB0230124    ...............................................FVKDGIFRTGQQSSPYLAPELFNSN..........CNQYDTNSDQFGFAMILLQLFTRS............
DDB0229871b   ..............................................TQENEESLKEIKGNFLYSPPELLS............LNTYSNKSDIYSLSIVLYELFETCLTK......TYK
DDB0229850    ..............................................LNTEYINSNNQNKSFAFCSPESSDFQDID.DDYTSLSSSITSKSDIYSFGIIMFELISRIING......EYS
ARCK-1        ..............................................TESNQETLNKTRGTSVYCAPEVFE............GKEYNERSDMYSMGIVMWEIVYCVVYG......CYM
```

```
                                                                  Subdomain VIII                   Subdomain IX
SAPKalpha     ..............................................TGSNLETLVKMRGTFAYCAPEVYY............GEQFSGKSDVYSIAVILWELVTRCING......RYE
DDB0229849    ..............................................TADNLETLSKIRGTFAYCSPEVAVG..........NGSLYTTKSDIYSIGIVFWELVTRVING......EYS
DDB0229848    ..............................................TGSNLETLGKLRGTYAYVAPEVYF............GKKYTTKSDVYSMGIILWEMTYRCIKG......THL
Phq2          ...............................................MTNVSTLGMLRGTMAYTAPEIYD............GLLFNTKSDVYSLGVIMWEAIQRCISG......VYL
DDB0229847    ...............................................TAKSASNRTTRGTFAYCAPEVF.............YGIHTTKGDIFSIGIILWELAVRCIKS......KYE
SplA          ...............................................MEQASQMTQSVGCIPYMAPEVFK............GDSNSEKSDVYSYGMVLFELLTSD............
DPYK4-b       ...............................................SNQSESKKLRGGSIHYMAPENLN............GSPINEKSDIYSFGLLVWQMFS..............
DDB0229872    ...............................................KPGPTEMTAAMGSLAWMAPECFK............AENYTEKVDVYSFAIILWEIVTCR............
DDB0229844    ..............................................TELQTGSNSTIKGTMAYCAPELYN............GISYSEKADVYSLGIVLWEITTRVITG......KYQ
ZakA-b        ...............................................SNQSESKKLRGGSIHYMAPENLN............GSPINEKSDIYSFGLLVWQMFS.Y............
7TMK1         ...............................................EQGFE.MTSTVGHLCYQAPEVFI............GELYTPKADVYSFGLLVWCIITGE............
rk1           ...............................................IEQQGATMTACGTPCWTSPEVLR............SQRYTEKADVYSFGIILWECATRQ............
rk2           ...............................................IEQQGATMTACGTPCWTSPEVLR............SQRYTEKADVYSFGIILWECATRQ............
rk3           ..............................................KHLDKKTAMTPVGTPCWTAPEVLR............NDPYTEKADVFSFAIVLWEIVTRE............
DDB0214883    ...............................................VKSHTFAKTMCGTTGWVAPEVLA............EEGYTEKADVYSYAIVLWELLTRL............
Shk1          ..............................................GENLKDGQDGAKGTPLWMAPEVLQ............GRLFNEKADVYSFGLVLWQIFTRQ............
Shk2          ...............................................PDSKDKLLGQAGSPLYMAPEMLV............NQPYDGKVDVFSFSILLWELLTKQ............
Shk3          ..............................................KHKMLKDQSSAKGTPLYMAPEVMM............FKEFNESSDVYSFGIVLWEILTRK............
Shk4          .............................................IKEGEEFQDKAAKGTPLWMAPEVMM............GNPYNEKADVYSFGIILWEILTKE............
Shk5          ..............................................TGTTLSDQKGPKGTALYMAPEVMM............KHEFNEKADVYSFGLILYEMATCE............
DDB0229963    ...............................................ETSFTQTMTTIGTVAWTAPEILR............HESYNQKADVYSYAIVLYELLTGE............
DDB0231199    ...............................................IDNNFNMTAVAGTPKWESPECLM............GEAYTSASDVYSYGMMLFELATGD............
DDB0229940    ...............................................VDLAKQMTLNLGTSCYMSPELFK............GNGYDETCDVYAFGIVLWEIIARK............
DDB0218878e   ...............................................VDLAKHMTMNLGTSCYMSPELFK............GNGYDETCDVYAFGIVLWEIIARK............
Rck1          ...............................................VANKYNMTTHVGTQAWMAPEIFT............SRTYTNKVDVYSYAIILFEIFTRK............
DDB0231197    ...............................................FVEARLGYTIVGTPNYMACELFN............GQPYHQPADVFSFGVVLWEMFTQD............
DPYK2         ..............................................REGSMTMTNGGICNPRWRPPELTK...........NLGHYSEKVDVYCFSLVVWEILTGE............
DPYK3-b       ...............................................TLSDDMTLSGITNPRWRSPELTK............GLVYNEKVDVYSFGLVVYEIYTGK............
Gdt1          .............................................IKGLACNNYIERVDTPINLTREISIIRDPK(29)NNSNNSSSLKYNIHSFAVLSYELLIDE............
Gdt2          ...............................................TNERYNLQTRRGTVAYMSNEILS............QGSYTKSTDIYSFGMLCYELFSER............
Gdt4          ...............................................INGKHELNSKAGTTRYMSPEILE............GLPYTYSTDVYSFAILFFELLSGR............
Gdt6          ..........................................................EVQNQNEKEIFK............DEKDQKLIDVYEFGITFYETMLEE............
Gdt8          ...............................................LGEKESKSKPDSTMNYLPNEVFD............GEGYQKSADVYAFGVSLYEVMVEK............
Gdt9          ...............................................NEFSNKNTNCVGTPIYMSNEILE............KKTYDNSTDVYSFAVLFYEMMIEE............
KinX          ...............................................EEDNNKSMTICGTDNWMSPEMIT............GLDYDERSDIFSFGIVLLEIISRVK..........P
KinY          ..............................................KKQQGNRHLSICGTDSIMAPELIL............GMEYDESVDIFSFGVVLLEMILRKKV........SK
DDB0229853    ...............................................DGKDTNNLTICGSENTMSPEVIV............GSSYNDSCDVYSYGVLLLELICGS............
DDB0229863    ...............................................EERINKRYTCVGTEIWMAPEVCL............DQSYSTAVDVFSFGIVLVEIITEKI.........PD
DDB0229866    .............................................GGGSRAKRTMTICGTTNCMAPEVVL............GQDYNEACDVFSYGIVLSEIITRMDTTNNL...RPS
DDB0229851    ...............................................DKSEKSKMTICGTDDWMAPEVLI............GEEYDASCDVFSFGMVLIELITRE............
DDB0229865    ...............................................TIQNPLSMTICGTDEFMSPEVIL............GIQYSYSADIYSFGMVLLELIT.R............
GefX          ..............................................EQTKKSRHMTMCGTEGWVAPEILL............GMSYDTSCDVFSYGVVLAELITG.............
DDB0229867    ..........................VNGAAGGGGDDVYKNNGNGKPANYRLRRMSICGTPSFMPPEILL............QQKYDWSVDVFSFGILCTELIT..............
HH498         ...............................................NDIGDKTMTSIGNPRFRSPEVTK............GQKYSEKVDVFGFGMILYEMFTRR............
GbpC          ..............................................ALYGGAALIRCVDQPLWLGPEVLA............GTAYSEPSDVYSFGIILWELYTRA............
Pats1         ...............................................GLQHTITNRGVENPLWLAPEILN...........KTKEASTQTDVYAFGVILWELVTRK............
QkqA          ...............................................QQSVYSVSGLLGNFQWMAPETIG..........VEDESYTEKVDTYSFAMILFTILTGE............
Roco4         ...............................................QQSVHSVSGLLGNFQWMAPETIG..........AEEESYTEKADTYSFAMILYTILTGE............
Roco5         ...............................................VVTSALGRDKLSNPIWLSPEIMR............GDEYTEKADVYSFGIVLWEILTGL............
Roco6         ..............................................QALKHKLRNFPVGNITWVAPEILR............EEEYTVKSDVYAFGLILHELLTRK............
Roco7         ..............................................KPIGKEVKGRKVWNWRWLAPECMG............DGQYTEKIDIYSYAIVLWEIITRD............
Roco8         ...............................................SIVQNFSSKVVDNPTWQSPEVLK............GMEYNEKSDIYSFGMILWECYHLE............
Roco9         ...............................................GFSTTVANRSVQNPVWLAPEVIN............NELCSDKSDVYAYGVILFELLSRT............
Roco10        ...............................................SFLPSLFPTSTTPKSYHSPEVLQ............KLNYHENSDVYSFGIILYELLTRS............
Roco11        ...............................................QQSLYSVSGLLGNFQWMAPETIG...........AEESYTEKIDTYSFSMILFTILTGE............
DPYK3-a       ..............................................PKLNATDLNNPAIEPRYMAPEMTR..........MEEDQISCSIDVYAYAFVLWEALTSH............
DDB0229956    ...............................................VVDNR.MTKTVGTPCYMAVEVLK...........GLTDYSQQADVYSFAFVLWECISRQ............
DDB0229957    ........................................NRHTGSGDNNKVEKDSKKGTTLWMAPEVIR............GAMYSEKCDVYSFAIIMWEMVTRK............
DDB0220138    ............................SSTTTSSANGANSISNNNNNGTTSVDQSRLAYAFYAAPEILN............SKHFSEKSDVFSFGTILWELVTNK............
DDB0220436    ..............................................NNQNNNNNSLNMGSYRYSPPELLCNN.......NQNNNLINKSVDVFSFGIMLWECLNWT............
DDB0230133    .............................................TSSSRSFYGPTVEGSFLYMSPEVFT............QKKFSRAADVWSYGACLIEILTNK............
DDB0229871a   ...............................................GGIGSSAKTFCGTLSWIAPEVFN............GSGYTTKVDVYSFGIVLWEILTHK............
DDB0229955    ...............................................KSKSTEMTKSIGSPIWMSPELLM............GEDYTEKVDVYAFGIILWELGTGE............

Bud32         .................................................................................SNSVEDKAVDLY........VLERA............
DDB0231196    ............................................DENEKRLNTFAKTIGTMWYRCPRLGDCSED.....EKTLTHYNEKSEIYSLGIILWELVCVAMTGTYI...SPK
```

```
                                                                 Subdomain VIII                    Subdomain IX
DDB0229339  ............................................DENEKRFNTFAKTIGTLWYRCPRLGDCSGD.....EKTLNHYNEKSEIYSLGIILWELICIAMTGTYV...SPK
DDB0231195  ..............................................TESNGCTLQQIRGTNLHIAPECYK............GFLFSSQSDIFSVGISLYQLAYKLVYG......KFV
DDB0229335  ..............................................ETNENT.LQKYRGTSVFIPPELND............NILYNEKSDIYSLGVSFMMLLYKVVYG......KME
DDB0216331  ...............................................KYENS.MSGVAGTYYYLSPEILL............NKNYSRPADIFSLGCIFYEMYTLNL............
DDB0229344  ..............................................AHHTGSIHSETVGTYCYSSPEILN.............STYDKTTDIFSLGCIFYELITLK.............
DDB0231281a ...............................................TLYTQNKSTLIGTNQYIAPEVIE............KKGHTNLIDIYSLGKTLLSLSKRN.............
DDB0230037a ...............................................QVKTINSSTLIGTNQYIAPDVIK............RGGYTGTIDIYGLGKTLLLLLN...............
DDB0219988a ...............................................MVITDQKNTLIGTNQYMAPEIDLG.............GYTCKVDIYSLGKTMLSLIT...............
DDB0231281b .............................................ELKNDQDLYYSKDGTLGYQSPEIYSSEL....RGNGDIKNQPYKMDVFGLRCVMKYLMSK..............
DDB0230037b ...............................................GKHLENYYSRDGTFGYQAPEIYREEL....RGDGDIKTQKYKMDVFSLGCTMAFLIKK..............
DDB0219988b ...............................................DFGFILKTNESGSKMYQAPEIYQEEYRS...KENDITNQHYKLDIYSLGFTLSHLMKK..............
DDB0231182  ...............................................LPSNEIIYNQRNDR...SKEFC..................FYSDLYSFSSLLNKIKIKE.............
IksA        ..........................................LAPPLYKNNIKRTGNTGTIEYLAPELLQKGV....NGEYNSDYDEKCDIWSLGILLYQMAYGT.............
IreA        ............................................LDNDDQ.SLTFTSDSHGWQPAEYLN............GTNRNTKKVDIFSLGCVVYYLLTGA.............
IrlA        ............................................VETSYSF.TNNAPTGQEGYHPIEVLQ............EKRKTKSVDIFSLGCILFYLLTNGQ............
IrlB        ............................................VESSYSF.TSNIPTGQGGYHPFEVLQ............SKRMTKSVDIFSLGCILFYLLTNGQ............
IrlC        ............................................VTSSYSF.TMHAPTGQEGFHPAEVLL............EKRKTKSVDIFSMGCILFYLMTGGQ............
IrlD        ............................................VTSSYSF.TMHAPTGQEGFHPAEVLL............EKRKTKSVDIFSMGCILFYLMTGGQ............
IrlE        ............................................VSSTYNFSTNAIPTGQDGYHPVEVLL............EKRKTKSVDVFSLGCLIYFIMTNGA............
IrlF        ............................................VESSYSF.TTNVPTGQGGYHPVEVLQ............SKRMTKSVDIFSLGCILFYLFTNGQ............
DDB0216373  ..........................................NRKDALSLQDEVEQNTTPFYRAPELFDIPS..........DCQIDERIDVWALGCLLYTMAYNN.............
DDB0229347  ...........................................ATERGKAEDDINMFTTLFYRAPEMVDLYR..........GQIIDEKVDVWALGCLLFKMAFYV.............
DDB0229350  .........................................KNKTEMQNAEDDISRHTTLQYRAPEIVDFYR..........SPVINEKIDIWALGCLLYKLLFYV.............
Nek2        ...............................................LNESLYAHTFVGTPYYMSPEQIH............GLKYNERSDVWSVGCLIYEMATLS.............
Nek3        ...............................................NSETSLASTVLGTPYYLSPELIQ...........NEKGYDHKSDIWSLGCVLYELTTLK.............
Nek4        ...............................................LNSSEFAKTMIGTPYYMSPECFG............SRAYDFKSDIWSLGCCLYEMITLK.............
DDB0229345  ...............................................SETDLYANTTAGTPQYVSYEICN............KKPYTNKTDIWSLGVVLYELMTLS.............
IfkA-b      ............................INLSSSTNSTAQQTPMWDLNDENLSMTGGVGTPFYCCPEILE..........KNTKHYGTKVDMYSLGIIFFEMCFQ..............
IfkB        ............................INLSSSTNSTAQQTPMWDLNDENLSMTGGVGTPFYCCPEILE..........KNTKHYGTKVDMYSLGIIFFEMCFQ..............
DDB0216407  SVIN.....TKQQQQQQPQNYWEDEVEGQQQQQQQQQQQQQQQQQQQQQQQQQHTARVGTLFYTSPEQEA........GTNGDSAYDDKVDMYSLGIVFFEMWY...............
DDB0229432  IPISTPPTS(21)AATTHLNSSLGCTISSSSSTTTATATTSTTPFMFYNSISVNTVGVGTLTYASPEQLSNKGVF.GGGGYTNTWYTNKTDIYSCGIILFEMIVG..............
DDB0220611e ININQQLQQ(>63)PTGQSQQSIPSTPSSLSSSSTTPSVSTGTSLSRSLEHCKHTSAVGTLTYSSPEQ.............KKGLYNEKTDIYSLGIILFELYF...............
DDB0220652  .......................................NNNNNINNNNNNNKKVGGLGTYLYSSNEQEQ............GDNYNQKTDLYSSVGVIFFEMLSQFK...........
PLK         ...............................................VEHGERKKTICGTPNYIAPEILD...........NSNGHSYEVDVWSIGIILYTLLIGK.............
Vps15       ........................................FIPEDNPADFSFYFDTSGRRTCYIAPERFYETNRG....APSNNELTPKMDIFSLGCVIAELFLDG.............
TBCK        ..............................................LSDHGENVSFPIGNLLYLSPESIL.........RGAKGSSNTKADVWALGCILLHICLGY.............
TtkA        ..............................................DDTTNIVRESQIGTINYISPEALIDTSQ...GGPKQCMKLGRASDIWSLGCILYEMAFGY.............
DDB0220004  ..............................................LNEATNTKIYSEGDSRYLSRELLH...........DDMSALKKSDIFSLGCTLYELARCK.............
DDB0229384  .................................NNTSMDSDCNNLSLDEDDIFFDFLEGDSRYLAYEFLL...........DKKQISKPSDIFSIGVTFFEMVTGNEMP......TNG
DDB0216398  .................................................TTTGGDDSEGDQIYMAPELLN.............DIHTPSADIFSLGITLYEMATNY.............
Scy1        .............................................INNSILRNHNDLIPN.KYKSPEIMKSQ.....WQQIQQSPSYSIDSWMLGCLMYECYNGT.............
Scy2        ....................................REYQYISGGGGGESSNNSNYILPQLDYLAPEFIS............QRKFETNSDLFSIGRLIFELSINLEQKALDSHLISQ
SLOB1       ..........................................................LGMKPLFNDYIYG.......................IG.LPQSFKDNLE............
SLOB2       ..........................................................LGLEPRFSDFIRQ..........HNEKIDP..DVLSFGLVLFEMACG..............
DDB0229334  ........................................................NFLNQDYQSPEVLK............SNNFTHKSDIWSFGFILLKMIDK..............
DDB0231326  ..............................................TQDVEGYTTRNRGTEFIKSPEMLTIAYAS(7)DRRKKVGSNTASDVWSLGCLFYELLTGD.............
DDB0231559  ..............................................CTLNLSSLKNGAGTVCFQAPEASR.............GKATIQSDIYSLGVVLFELCGAIPYKNY....NYF
DhkG-a      TMID.......NIDLTNSYNNNNNSSTNNIFFNFNNDYDEESINELFKSYPDLSRFSTSTYYYISPEDTGRTIH..........PVDEKSDLYSLGVTFFELLTGR.............
DDB0231198  ................................................LVHDNNFTSTFHTDDYVPEEYG...........RFQYDGEKVDIYSFGCTFFEMVTKHR............
DDB0231179  ................................................DVPIRLCDTNGHENYKSLERYWDYNQC...RIKTTSFIRDNEDIFSLGVLIFEMLLTF............V
DDB0231335  ................................................................................SNIGDDRKGYDISNLKSIYYDFFNNY.............
7TMK2       .....................................NPTALQQQKLNNFFQSATTTTTTEQTTEPIEVAA......TTTPQTIHSAFQHDILSFGFIVLDFLTLSN............
DDB0229381  ..............................................GNDSAEIHSTLAGSPLYMAPEIIHIILSP(11)DSSEGYNPLLVDVWAIGAVAFRLITGDDL.........IS
Bub1        .............................................IYSQDARYQSPIELLPIGTPLEWLKI.....NQSSTTNVGWSYNLDYLGICKVMFHILTCGK............
DDB0230007  ..........................................................SIYYLSPEVLK.............GYTYSFSSDIWSFGVLLFKCITGY.............
DDB0229337  ......................................................QFFFQDYLYSSPENYK..........GSLVGVNEYSMVWTLGIILYQCLTGE.............
DDB0229346  .......................................................................................................................
LvsG        .......................................................................................................................
DDB0230125  ...............................................TENEEFYQGTMNTASNRIYNILIN......NRTNHAFSVVESDDLESL.VKVYIMENEQ.............
DDB0204911e ...............................................TENEEFYQGTMNTASNRIYNILIK......NETNHAFSVDESDDLESL.VKVYIMENEQ.............
CK1         ............................................THQHIPYREHKNLTGTARYASLNTHQ............GIEQSRRDDLESLGYVLMYFNRGS.............
DDB0216336  ............................................SGEVRPARESTGFRGTARYASINSHL............SKDLGRRDDLWSIFYVLIEFAEGQ.............
```

```
                                Subdomain X
Akt1        PPFYNQ...............DVQEMYRKIMM.....................................................................................
DDB0220670  PPFYSE...............DPHEMTKLILS.....................................................................................
DDB0216386  TPFVGD...............NQNMIFEKITNYT...................................................................................
DDB0220701  PPFNDD...............TPELIFQHILHRD...................................................................................
DDB0220700  PPFQEDEPNESINPKSDNDEKNVRIIFNKITNHK...................................................................................
DDB0185113  SPFNGS...............SVQETFQNILQ.....................................................................................
DDB0229452  TPFIGE...............TPEEIFESILS.....................................................................................
NdrA        PPFLSD...............NTTETCLKILNCKETL................................................................................
NdrB        PPFCSE...............SIRETYHKIMNWKQTL..............................................................................PK
NdrC        NPFCAD...............TPNDVFINILRWKETL................................................................................
NdrD        TPFNGD...............TPEEVFMNILDWKNIL................................................................................
DDB0231558  PPFDGD...............SPEEVMETVLKWK...................................................................................
DDB0216387  ALFVES............PDTTEQKIRENIGNWRGLL................................................................................
Pdk1A       VPFRGK...............TEFLTFQKVSN.....................................................................................
Pdk1B       LPFKGF...............NEYQTFLLITK.....................................................................................
PKA         PPFYDD...............DTFAIYNKILA.....................................................................................
pk4         PPFVSE...............GSQNDIFRLIR.....................................................................................
DDB0185224  LPFSNSK................ESLLNRKAD.....................................................................................
pk2         PPFYSQ...............NVSTMYQKILN.....................................................................................
DDB0220702  PPFEHK...............NRALMQEKIIS.....................................................................................
pk3         SPFLAS...............NRNDMYKSMIQ.....................................................................................
Aurora      SPFTSD...................EEKNIFH...................................................................................NI

MLCK-A      PPFYGD...............TVPEIFEQIME...................................................................................AN
DDB0216385  PPFYSD...............DESEMFELITK...................................................................................GQ
DDB0216309  FPFWDK...............NNAVLYEKIRN.....................................................................................
pXi         PPFDET........................TG...................................................................................YV
DDB0216308  APFYAD...............THHELFQKILD...................................................................................LE
DDB0229351  PPFQGK...............NNMIVMSLIIK..................................................................................GEL
DDB0216307  PPFYAS...............SQNLLFEKILT...................................................................................AD
DDB0216312  PPFYSE...............DIRKLFESILS...................................................................................AS
Snf1        LPFDDE...............SIPMLFKKIRE...................................................................................GV
DDB0229364  LPFDDE...............NIRRLLNKVKN...................................................................................GA
Lkb1        FPFSGA...............NMFVLFENISK...................................................................................CK
MARK-A      LPFDGD...............NYVELFQKILA...................................................................................GN
MARK-B      LPWTLT.............DGVQVEGMDRLLR...................................................................................GE
MARK-C      LPWEGD...............SQAEISFNSVH...................................................................................GN
DDB0231454  LPFINV...............TDMIVGKFILP.....................................................................................
DDB0219986  NNSDGNENDNN......NNNNFLIIREPFLLP...................................................................................EE
FHAK-A      PPFDDD..........DDEEMSTPQLFEKIVS...................................................................................GN
FHAK-B      KPFIVDC................RDLYHSFIN...................................................................................GN
FHAK-C      PPFDFDH..............TNNFNLKLINQ...................................................................................GL
FHAK-D      PPFGDP...............QTKDFFEKIKN...................................................................................GG
FHAK-E      PPFDDS...............REVSIFEQIRN...................................................................................AK
DDB0220010  TPFISK.................TNSLLDIYD...................................................................................QI
Apg1        PAYNSG...................SVPDLLN...................................................................................QL
tsunami     PPFSAN...................SLADLVHMTLES..............................................................................NI

Cdk1        PLFSGD............CEIDQIFRIFRVLGTPDDSIWPGVTKLPEYVSTFPNWP.............................................................
Cdk5        PLFPGS............GTSDQLFRIFKILGTPNEESWPSITELPEYKTDFPVHP.............................................................
Cdk7        PYLPGT............GEIDQLRKICSALGTPNESNWPGVTCLPNYIK.FTDHP.............................................................
Cdk8        PLFPGKEKDPKI...PSLFQDDQVEKIIRVLGKPTLDMWPDIKHLPEWKRLSSMEAF...........................................................P
CDK9        TLFPGR............NSIDQLDKIYQVCGSPNANNWPEAMDLPFWDALKPKRE.....................................................YNSLSLK
Cdk10       PLITGN............NEVDQIMRIFNLLGEPNEQIWPGFSSLPNFKRLNNIPHQ...........................................................P
Cdk11       VLLQGS............SEIDQMDKIFKLFGTPTEKSWPAFFKLPLAKYFNLTDQ............................................................P
DDB0229294  NFLPGS............SEQQQIEAICKLCGTPTDEIWPNVSQLQNFNQISHLPV............................................................Y
DDB0229424  YMFAGS............NDSLQLELIFKTFGTPTEKNWPGISKLSGYSPYLGSKSKKY........................................................PSK
DDB0230050  YLFAGA............DETAQLDLIFKLFGCPTEKSWPGVSSLPGYNDLFNKQQQQQNE(38)YNNISTSCLQSPSSSPPIGGYASSLSSDFNSNEYNGYNQTFSNEDCLAISQK
CK2         DPFFHGH...........DNIDQLVKIVKILGTEEFYAYLDKYGIVVDHTILSIIGKHPK.......................................................KPWS
DDB0219953  DPFFNGD...........NNNDQLLKIVKVLGTDDLFNFLDKFGLSLTDEQSSLIKP.......................................................RQKSNWE
ClkA        TLFDTH............NNVQHLAMMEKVMG.PFPNSMSNVSKKYFNDYGTLNRPQNSDE.................................................IKSMERVEG
DDB0229841  RLFRSF............NSKEHLKSIYKLIGAPKASEGAYVVKGELLFFLMEYNRKN..........................................................SF
DDB0229333  QLFRGE............SKQSLLIEISELIGTPTYDECKESTNGFARNFILNIPLK...........................................................SR
DDB0229296  RLFKTQ............DFEDHLISISKLIGNPTAEDLSIVLSKSIFQYMEKIPKST.........................................................LTP
Dyrk1       PLFSGQ............NEQDQLTKIIEVLDLPPSHMIDSSPKAKKFFTKDPINSTYQLKKNEKLKTNVEFC........................KKKLSEIIGVETGGPQSRRKN
Dyrk2       PLFPGS............DEPEQLACIMEVLGAPPKSVIDNSTRKDIFFEDDGTPKPVKNSTT.................................................GELYTIG
```

```
                                Subdomain X
YakA         PLFPGN............SEYNQISRIVEMRGIFPSDLLDKGKSSTRYFHRHLGSNSDDNNNNNNNNNNNGKPYYYTLKSEEDYQRD.SKTTLLPSKKYFNYKTLPEIIQNYGFKKSMSP
Prpf4B       FLFPGK............TNNDMIRLFMEYRGAFSKKMLKKSEFVSNHFNENLVFMKQEIDNIEKTVRKVPH................................DITKPTKDILQFLLPK
GSK-3        PLFPGE............NGIDQLVEIIKVLGTPTKEQIHAM...NPYYTSFKFPEIKA............................................................N
GlkA         PLFPGT............NSNDQLGRIIEVLGSPTKDDMEAMKPSKPYH.L.QLPNI..............................................................N
Erk1         PLFQGK............DYIHQITLIIETIGSPSEEDICNIANEQARQFIRNMGNQ..............................................................P
Erk2         AMFPGN............STMNQLDLIIEVTGRPSAEDIEAIKSPFAGTMLESLPP..............................................................SN
DDB0229430   PMFPGS............SEIDQLFKICTIMGSPTSATWIDGIKLANSMGFTFPNVQP............................................................PS
Sky1         HLFKPKSG......KGFEKSDDHLALMIELLGKPPRFIFAGGDESRVYFTHKGDLRKIPDLSD..................................................QWPLFSV
DDB0230051   KLFNNLNLDNNNNN(52)QLYSVLNNISTYKESAPKNGNLYFKENHNIIIKSKVERDFNGANLPT.......................................................
DDB0230126   PLFHGH............NNKSVIESIVKIVGRPFQKDIDRMIANGNMTSDAIQLLQYAASL...................................................PVPFEDS
Cdc7         YPFFIS...........PDDMTSLAEIVSIIG..TKKIVDIAHLLEKKISISHSIPPTPW.....................................................RDLSRRL

DDB0229971   PPYFDL..................NPLGAMYRIC......................................................................................
DDB0229896   PPYYDL..................GQMPALFRIV......................................................................................
DDB0229877   PPYWDL..................GTMPAMFAMV......................................................................................
DDB0231212   PPYFGL..................APAAALYKIV......................................................................................
DDB0229915   PPYYTL..................QPMAAMFRIV......................................................................................
FNIPK-A      .............................LFEKD......................................................................................
FNIPK-B      LEETEI..................QIDGASFNNNNNKP..................................................................................
FNIPK-C      ...DPK..........................IT......................................................................................
FNIPK-D      ..CDCT.......................KDENI......................................................................................
FNIPK-Ea     LKLLDI..................................................................................................................
FNIPK-Eb     NLCHSE..........................RD......................................................................................
DPYK4-a      .............................NPKKI......................................................................................
ZakA-a       .............................DLSQK......................................................................................
DDB0230002   EIKPIK..........................LT......................................................................................
DDB0229378   ..........................LNQLCLQR......................................................................................
Mek1         FPYGSNLPHQQQQP(83)SVPEGMGFWVLLDCIV......................................................................................
MEKKalpha    PPWSNI...............TELAAVMYHIA.S......................................................................................
DDB0229911   APFAEY..................PPMKVLLLTLQN..................................................................................PP
DDB0230012   PPGYDL..............PPSKVFMNLLFGNSPS....................................................................................
MkcA         NKLR.K..................NPIKTMFMVGSEG...................................................................................
MkcB         PPMMES...................AVKAMFTVATD....................................................................................
MkcC         PPHR.K..................SSLTAMFTTATE....................................................................................
MkcD         LPYHDS...................RLKAMVFVATNNL..................................................................................
MkcE         VPHH.K..................SKIKAMFLAGTVGVK.................................................................................
MkcF         PPHRDS...................RMKAMFYNAIN....................................................................................
PakA         PPYLEF..................PPLRALFLLTTQ....................................................................................
PakB         PPYMSF..................PPLRALFLITTK....................................................................................
PakC         PPYMDF..................PPLRALFLITTK....................................................................................
PakD         PPYAKY..................PPVRALFLLTLE....................................................................................
PakE         PPNRGL..................RSMNQLVEIPNM....................................................................................
PakF         PPHIDL..................PPMRAMKMVPNW....................................................................................
PakG         PPLYTM..................PPTRAMLMIPNK....................................................................................
PakH         PPLSEV..................NPMRAMYMIGRR....................................................................................
Krs1         PPLFNV..................HPMRVIFMIPNPSR..................................................................................
DDB0216375   PPYHDL..................HPMRVLFMIPTS....................................................................................
SvkA         PPRADL..................HPMRALFLIPKD....................................................................................
DDB0216374   PPLFNL..................NPVKVIFVIPFR....................................................................................
DDB0216379   PPRFDV..................PPSRVIFTIPHQ....................................................................................
DDB0216377   PPNSNV..................HPMRVIFMIPRE....................................................................................
DDB0216378   PPLSEI..................HPMKVLFQIPYR....................................................................................
DDB0230010   VPYGKD..................SSLKALFYTAIH....................................................................................
DDB0229972   PPFSHL..................GPMEALIEIINPSTTI..............................................................................LP
DDB0229973   PPYHQF..................QHLQSIEMIINGTC................................................................................PP
IfkA-a       PPLQYQL................QQQYDLIKEIEQ.....................................................................................
SAMK-A       ESYSKK...............ENNQPNLQKLP........................................................................................
SAMK-B       DPWGGK..............RNHQSHSPPIPT........................................................................................
SAMK-C       ...YII..............TTPIIILPSIPS........................................................................................
SAMK-D       TDFKEDE..........EGFEELKRTFAGVLT........................................................................................

DDB0230038   ....RD...........IKLSDLKSNTVNGYT...................................................................................HYLRP
DDB0230124   PLFQDI................HVSRITDTIL...................................................................................NGVRP
DDB0229871b  KPYHEV............TLDFDFQIIHKTSK...................................................................................LNLRP
DDB0229850   HPFSEF...........KDIKNDFQLLL.SSK...................................................................................NGLRP
ARCK-1       IPYQEY..............NKMFNAFQVALL..............................................................................VNSSKRVLRP
```

```
                                      Subdomain X
SAPKalpha      RPFSEY...........KNLQHDFQIIIQTAK................................................................................KNLRP
DDB0229849     RPYSEY...............SHIKMDFQIML.............................................................................NSKEGLRP
DDB0229848     LPYAEY...............PHLKFDYQILI............................................................................SSAKKDVRP
Phq2           RPFHEH............PISMDIQIIILTSK................................................................................NKVRP
DDB0229847     KPFSEF...........KHIQYDFQILVQTSK................................................................................FNLRP
SplA           EPQQDM...............KPMKMAHLAAY................................................................................ESYRP
DPYK4-b        .YASPN............TIYSPKEMASMVSD..............................................................................EKLNYRP
DDB0229872     DPYNGM...............EPLRLAFLASV................................................................................EDYRL
DDB0229844     RPYEDN...........TEISFDFQIVIMSSK................................................................................QGIRP
ZakA-b         APPNTI..............YSPKEMASMVSD..............................................................................PKQNYRP
7TMK1          QPNQNL...............QPLKMAHLAAY................................................................................ENYRP
rk1            DPYFGI...............PPFQVIFAVGR................................................................................EGMRP
rk2            DPYFGI...............PPFQVIFAVGR................................................................................EGMRP
rk3            DPYQGM...............PTFQIVISVGQ................................................................................HKLRP
DDB0214883     IPYAGK...............NTMQVVRSIDR.................................................................................GERL
Shk1           ELFPEF.............DNFFKFVAAICEK.................................................................................QLRP
Shk2           EPYNKL............YSSYPQLVEGVVNK.................................................................................KNRP
Shk3           EPFSHH.............RELEKFREAVCVK.................................................................................HERP
Shk4           APYSHH.............KDYDIFFNAICNE.................................................................................KERP
Shk5           ELFPEY.............SEIDPFYDAICNK.................................................................................KLRP
DDB0229963     EPYQGI..............PPMNAGILVASK.................................................................................GLRP
DDB0231199     EPFLEI.............QSIVELARSVCDK.................................................................................KLKP
DDB0229940     EPYENI...............NSWSIPVLVAK.................................................................................GERP
DDB0218878e    EPYENI...............NSWSIPVMVAK.................................................................................GDRP
Rck1           SAYDEN...............ANINIPNMVMK.................................................................................GERP
DDB0231197     TPYKNL...............TRIEQALFVQS.................................................................................GGRP
DPYK2          IPFSDL..............DGSQASAQVAYA.................................................................................GLRP
DPYK3-b        IPFEGL..............DGTASAAKAAFE.................................................................................NYRP
Gdt1           ILVGDT........................RK...............................................................................FGQEKP
Gdt2           IPYYSE...............PHWRIRELVLK.................................................................................NERP
Gdt4           VPFREI...............PKREIPRFVRL.................................................................................GTRP
Gdt6           TPENLS.....................NLILE.................................................................................GERP
Gdt8           IPFHEI...............CYNDIPNRVQD.................................................................................GFRP
Gdt9           VPFSEI.............D.EKWEIPSKVIS.................................................................................GWRP
KinX           APYMRD...............ASFGLAEDIVR...................................................................................NQ
KinY           VLERGP...............QSAFEIDQDSA...................................................................................RQ
DDB0229853     RVVKTQ..........LKRTPMNAFDMNLEKA...................................................................................EH
DDB0229863     ERFPQH.................RFQFDAPAF...................................................................................LK
DDB0229866     SLKYGL.....................DVDVL...................................................................................LP
DDB0229851     NLTPRI..............RNEDLGVDQKFF...................................................................................LS
DDB0229865     SKLDER................LPQNNFDIDY................................................................................EELQN
GefX           .RKPGV...........DLWVRSPETCFDINP................................................................................EELKQ
DDB0229867     LKRPGKD.......YWVRSQNNGFDINIEELN..................................................................................VNI
HH498          VPFHDY...............EQIAASFKIAN.................................................................................AERP
GbpC           HPFDEF...........QFGQWMSKLEDEIIR.................................................................................GLRP
Pats1          DYFGEI..............GFMTLIEEKVIN.................................................................................GERP
QkqA           IPFDEY..............SFGKMQFINLIR...............................................................................EENLRP
Roco4          GPFDEY..............SYGKIKFINMIR...............................................................................EEGLRP
Roco5          LPFDEY.........PVAHSSFMYQLEDEI.T................................................................................NGLRP
Roco6          HPYREF............NYS.MVSLQEDAIK................................................................................NGLRP
Roco7          LPFEEY..........VDQLKWNSIIEDKIMK.................................................................................GLRP
Roco8          LPFDEF............DFKFMSTLEDNILS.................................................................................GLRP
Roco9          RFFSNI...............TFMSEVEGLIT................................................................................EGVRP
Roco10         IAFQDH..................DRFSNIVI................................................................................SGQRP
Roco11         CPFDEF............TSFGKMEFIRKIRE................................................................................EDLRP
DPYK3-a        LPFRKF...............NDISVAAKVAY................................................................................ENLRP
DDB0229956     IPYKDF...............AQIQWISMVLE................................................................................DSFRP
DDB0229957     LPYSHI...............AFNCEVEDQVL................................................................................KGLRP
DDB0220138     IPFDGM................DPYEVKELLK................................................................................SGKRL
DDB0220436     LPYESL................SREQVKQIKT...............................................................................DMERES
DDB0230133     RPYQEY.............DQLGYLELARVRE................................................................................EGLPP
DDB0229871a    QPSGNI....................SATSLG...................................................................................HP
DDB0229955     LPYSGL................DSVQLALAVT...............................................................................TKSLRP

Bud32          FISTHP...............NSEQLFQTILS....NYELTS...........................................................................
DDB0231196     IPLFQNE.................VDFLIWIHKDYRFSFPVGTP.........................................................................
```

Subdomain X

```
DDB0229339   IALFQNE.................VDFSIWIHKDYRFSFPIGTP..........................................................................
DDB0231195   HPYHEYQI............ADEPENLNIIQNTGLIPTIPPNLP..........................................................................
DDB0229335   NPFYEF...........KISKMEYFKTVVALENFLVPIVPTFLP..........................................................................
DDB0216331   LPLTKRS..............FGQELIEGKFDRKAFKQEFDDSD..........................................................................
DDB0229344   LLSHNR............IYLGEDMLNDRFDSMQFLSTFPEKY...........................................................................
DDB0231281a  TGFVYN...............RIYLKLFEIMTNPFETR................................................................................
DDB0230037a  .........................RVQQNQY......................................................................................
DDB0219988a  .LVHSP..............MNSIFESLFQLCTLENFKIR..............................................................................
DDB0231281b  ..FNSS................................................................................................................
DDB0230037b  ...........................FNITS......................................................................................
DDB0219988b  ..FNCNP...............................................................................................................
DDB0231182   SPSSSS...............TTSTSTSQVLIKSDSDSSSSASSSSSSSSSSSSSSSSSLP....................................................KSKNK
IksA         LPYRYSGDPF........IDEDPNRNLPSLIDEIAGFSNNRLIFPQIPQRS...................................................................
IreA         HPFGHRY.................NREKNVLKGKFDIDQIKHL...........................................................................
IrlA         HPFGNN................KLLRVANIVYDKPDLEPLKFN...........................................................................
IrlB         HPFGND................KLFRIVNIISNKMNLTPLNSN...........................................................................
IrlC         HPFG.D................KFYRIVNILTDKPILEPLKHN...........................................................................
IrlD         HPFG.D................KFFRMANILTDKPILEPLKHN...........................................................................
IrlE         HPFG.D................KFSRLRYITKSKYNLSQLSNLN..........................................................................
IrlF         HPFGND................KLFRIYNIMLNKVNLELLGHN...........................................................................
DDB0216373   SPFEVC...........DNEPNGSVALKVLSG.....................................................................................L
DDB0229347   DPFDGG...................SLQIINN......................................................................................
DDB0229350   TPFEDS..................GSLGILNS......................................................................................
Nek2         PPFEAT...............NQAQLTSKIQV......................................................................................
Nek3         HAFNAA...............NLPALVLKILK......................................................................................
Nek4         HAFDAK...............EMPSLIFQILQ......................................................................................
DDB0229345   LPFDGR.................KETVMRNIQTESTIFKPVNHPN.........................................................................
IfkA-b       ..FQTQ...............MERSNILRDLRDNLKFPPGFESTK.........................................................................
IfkB         ..FQTQ...............MERSNILRDLRDNLKFPPGFESTK.........................................................................
DDB0216407   .VFSTG...............HERVIVLRNLREKFEFPSDFERNH.........................................................................
DDB0229432   .GFETQ...............FERTTHIKNLKNGIL.PSWFTSKH.........................................................................
DDB0220611e  .PISTR...............MEKARVLTDLRNGVL.PKSFLQKY.........................................................................
DDB0220652   TTMERS............TTLSKLKKSLSVLKTN....PNLKQKY.........................................................................
PLK          PPFETS................DVKHTYQRIKQNQYSFPDEPIIS.........................................................................
Vps15        FPIFDF...............SQLLSYRKGEYSPEPIIRQRIQD..........................................................................
TBCK         CIWQDN...............NPSIVTNRILHLSGYSTTQSFNSISKSFEQCELKKTIINEKQHEEYEEYDDDEDISEEEKEKRIKDIKLNNRDNMNNFINSLKREEKENSDSNSGSS
TtkA         PPFK.....................SFSNIISKYQAIINPHHKIEFPVHPN...................................................................
DDB0220004   PLPKSGM........EWDSIRNGILSFEKEDS...IYDDNKNDFS.........................................................................
DDB0229384   PLWEQL...............RSDKAIDFLEPGK.........YS.........................................................................
DDB0216398   NLPQKGQ..................WWRNLREGKIPFPENDDSIS.........................................................................
Scy1         MTKAED.................IKNLDQIPKQLHQAYQKS.............................................................................
Scy2         LPKLGVISYYNT.............MIEQVRRQSTMNTQRSDSA..........................................................................
SLOB1        VICF..................GHCLFEMIIGIPLGDHSNINSFIPLFP.....................................................................
SLOB2        ..........................YEMENPHSVDISIPAHCY..........................................................................
DDB0229334   ..SLNN....................IPDDLP......................................................................................
DDB0231326   FLFYDDD........WVKFFIRVTQPGQELITPERKSKVANI............................................................................
DDB0231559   FPFGDL............KYLRVASAVSNYLRPILHPVIP..............................................................................
DhkG-a       LPFQSS...................DLSELIHSHLAKKPPLVIDLNQNVP....................................................................
DDB0231198   YQYKHP.................TPLHRDFLTTDFQYIP...............................................................................
DDB0231179   HPGTTLFEQN........VLFLLKFHGIEIVPTGGPNNNYTTLKVAFSNDIRNFT.............................................................SN
DDB0231335   DTINHDKKEKIKNV(66)NLNSNQKILIDLLNILIEDEEILIPI..........................................................................
7TMK2        YSCFDSKFDTSN....YYDDDKFNNKSNTKIKKPDLPDYIKKDEKLW.......................................................................
DDB0229381   VIFPNL...............NQTTVLAALVNLAKMIDNGDFQKGLDSIPNE.IR...............................................................
Bub1         ....E....................ELKLSLSQQ....................................................................................
DDB0230007   YPFYSN...............DATKLIESIIYDNYLDPIIRLPSDNNDLIQQ..................................................................
DDB0229337   LPFPTY..................PKIVNFITSSSQNVEIPP............................................................................
DDB0229346   ......................................................................................................................
LvsG         ......................................................................................................................
DDB0230125   AVKRTI...........KSIPNSEIGLFRTMWEFFNTECFPQYSTL........................................................................
DDB0204911e  TVKRNI...........LSIPNSEFGLFRTMWEILKTEFYPQYSTL........................................................................
CK1          LPWQGL............KAYTKRDKYEKICDKKAQTKIDTLCQ..........................................................................
DDB0216336   LPWRKL............KDKDQIGEMKQKYNTPD.....LVKDLP........................................................................
```

Subdomain XI

```
Akt1          ..EKLSFPHFISPDARSLLEQLLERDPEKRLA......DPNLIKRHPFF
DDB0220670    ..SPLKFPPQISKNAASLVSLLLNRDPSKRLGSG..ESDVEEIKAHPFF
DDB0216386    ..NEINFPSYFSEVAIDLIQKLLQVDPLKRLGA....NGADEVKRHPFF
DDB0220701    ..REMEWPEEISSEAKDLILKLLNPDPYKRLGA....NGAYEVKTHPFF
DDB0220700    ..KKLYFPKKLYPVAIDLIEKLLDPNPSVRLGA....NGVDEVKCHPFF
DDB0185113    ..RNISWPEDMSPEARDLIDKLLALDPRQRLGF....NGAEEIKSHPFF
DDB0229452    ..RN.TECIEIQKDAKDLIIKLLDPNPSTRIG.......SKDIKNHPYF
NdrA          ...QIPTDMGLSKEVIDLIKRLVCEKDRYK.S.......ADEIKLHPFF
NdrB          IMEEAKAEVNLSPEAQDLIERFLT.DPMTRIGF....NGVEEIQSHPFF
NdrC          ..DWPLFTQELSVEAADLLKNMLM.EQPHRLGG....KGKQDFKNHPFF
NdrD          ..DWDLYSDYISEYAVDLLQKLIC.EPDKRIG.......IDEMKVHPYF
DDB0231558    ..TMLQRPDGVSDELWDLISGLIN.DGSTRLGSG..EKGVENIKNHIFF
DDB0216387    .NTAVQKNQPMSKQAESLIRECIAPE.RKRPD.......ASTIKKHPFF
Pdk1A         ..RELVYPINMNPVIKDLVEKLLVIKPTDRLGSSSTPGGFDNLKAHPFF
Pdk1B         ..REFSYPDNFDKCCMNLIDQLLDLDPYKRPT.......ISEIKNHEFF
PKA           ..GRITFPLGFDVDAKDLIKRLLTADRTRRLGAL..KDGALDVKNHRWF
pk4           .EARIQVPPEVDQVARDLIEKLVVTDVEKRLGSL..EGGIEDIKNHPFF
DDB0185224    .FQLIFQNSYLSDEIKDFIFQLLSVDPSKRLGT....FDSCSIRNHKWF
pk2           ..GELKIPTYISPEAKSLLEGLLTREVDKRLGT....KGGGEVKQHPWF
DDB0220702    ..EKAKFPKFVSSSARSLINGLLTKDPTKRLGA....NGAIEIKRHPFF
pk3           ..GNLRMPMFLSSDAQDLLEKLLVPDPNKRLGST...QGFEEISSHPFF
Aurora        QENDVYYPSSISPEAKDLISRLLVSDPHQRIT.......LKDVINHPWI

MLCK-A        YEFPEEYWGGISKEAKDFIGKLLVVDVSKRLN.......ATNALNHPWL
DDB0216385    WEFPSKTQHKYSDQVKDLIKLLLENDPNKRLT.......VKQSLAHKWI
DDB0216309    VDYGWPEGLEVSNEAKDLVSHLIEKNPEKRFT.......FEQCLIHPWV
pXi           LEFPSPEWDNISDSAKSLIKGLLNNDPSKRFT.......IDQTLKHPWI
DDB0216308    YDFPEPEWNGITDLAKDFISQLLIINPEERWT.......ASQCIKHPWL
DDB0229351    NFDSSVGWDNVSESAKDLIRKMLNPDPEKRLT.......AQQVLLHEWI
DDB0216307    YDFPEPEWTHVSEHAKAFIRNLIVKDPDQRHT.......AKQCLDDLWL
DDB0216312    YDFPNDYWGNVSKEAKHFINCLLTVEPTKRYS.......AKQALEHPWI
Snf1          FSIP.DF...VSPSCADLIKKMLVVDPVKRIT.......IHEIRNHPWF
DDB0229364    FSMP.PY...IHKDAQDLLTKMLTVDPSKRIS.......IKEIKEHPWF
Lkb1          IEFP.ND...LDKDLVNLIKGILQVDHIQRFS.......LGQIKNHPWC
MARK-A        YTIP.SY...LTHECKSLISRMLVVDPDKRAT.......MEEIINHPWL
MARK-B        FKYP.SHV.ILSDDVKDLINRMIVAEPVERAT.......LDEIKTHVWV
MARK-C        YEDP.TH...LSAEAVHILRRMIVPNPKDRAT.......IQELKNHPWT
DDB0231454    PSIP........LDLQDLIKKMLTVDREHRLN.......IMNVLNHPWL
DDB0219986    NNLPLPANH.SDQRLISLLSSIFQRNPKLRPT.......AHQIAVHPYF
FHAK-A        YRVEKLEKSLISSSAADLVKGLLTVDPDKRLT.......VEQALNHPWI
FHAK-B        LGFTSKKWAKISNYAKDLVRRLLVIDPEHRIT.......IKEALNHPWF
FHAK-C        YNFSLPVWDVVTENAKDLIKKLLNVDPTKRIS.......TKGALSHDWF
FHAK-D        FSFPSPYWDEISDEAKSLIKNLIKVDVEKRFT.......IDQALNHPWF
FHAK-E        FEFDPEDWSSVSEEAKDLIKRLLCVDPHKRYT.......CNNIIQHPWF
DDB0220010    VNHEPTYPREISNDLMDLFKRLLDKNPLTRIQ.......IAEIKSHKWT
Apg1          QNKKIKLPSHISSDCQNLIYSLLQIDVEKRIS.......WEDFFNHKWL
tsunami       EIPKELNKY...PDLVSLFRQLLCKNPDKRIG.......WPDLLYHPFV

Cdk1          GQPYNKIFPRCEPLALDLIAKMLQYEPSKRIS.......AKEALLHPYF
Cdk5          AHQLSSIVHGLDEKGLNLLSKMLQYDPNQRIT.......AAAALKHPYF
Cdk7          ATPFKQLFTAASDEAIDLISKMLLFNPSNRIS.......AADALNHPYF
Cdk8          NSLAKCVGIDENSQAYDLLSKMILYDPSKRIT.......ASEALDHPYF
CDK9          DFYQHENPSFFTKEAFDLLDKLLCMDPKKRIT.......ASEALDHQYF
Cdk10         YNNLRELVPTISDTAFDLLNQLLTYDPTKRIT.......ASDAIKHPFF
Cdk11         YNNLKSKFPHITDNAFDLLNKLLELNPEARIS.......ASDALKHPYF
DDB0229294    PSRLRTVFKNFSNDFIELLEGLLTLNPKKRLT.......AEQALQSPFF
DDB0229424    SLKDISKFKSFSNSTLDIILRMLTLCPKNRIS.......TKEALNHPWF
DDB0230050    KLIDKFPHLVLLPSVLDLASKMLTLDPKKRIN.......SLEALEHPWF
CK2           RFITKENQHLAVPEAIDFLEKLLRYDPAERLT.......TREAMEHPYF
DDB0219953    RFIPYENDDIAQPDAIDFLDKLLRYDPTERLT.......AKEAMKHPYF
ClkA          LKQLKEYFHPCHDSFFDLASRLLEYQPSKRIS.......ASDALSHPFL
DDB0229841    QPTFNNTFIGCNQIQIDLLKNMLCWDPRDRYS.......VNEILESPYF
DDB0229333    HGGIKKRIPNATEEEIEILEGMLCWNPKKRWP.......IERIIQHPYF
DDB0229296    SVGIKRRFKGASKDQIELLQGMLCWDPRKRMT.......IDQLLAHKYF
Dyrk1         EPGHALVDY...LKFLDLIEKMLIYDPQKRIT.......PLEALQHSFF
Dyrk2         TKSFKDSIRSGDEDFDNFILDCLKWEPSQRIT.......AEQGLKHDWI
```

Subdomain XI

| | |
|---|---|
| YakA | QDIEKEKQHR..IVFTDFINGLLQLDPNERWS.......PMQAKEHPFI |
| Prpf4B | NVSIPDQDMKKLIQLKDLIEKCTILDPEKRIT.......PFEALNHEFL |
| GSK-3 | PWPRVFKAKDVPAESIDLISKILLYDPSSRLK.......PVEICAHPFF |
| GlkA | PKFFESLHNVEDKTVVDLLSKIFIFDPVKRAS.......IDEIIAHPFL |
| Erk1 | KVNFANMFPKANPDAIDLLERMLYFDPSKRLT.......VEEALAHPYF |
| Erk2 | PRSLSDMYPSASVDALDLLKKLLQFNPDKRIT.......AEEALAHPFV |
| DDB0229430 | INPLSTLLPNANQDAIELITDLLQYDPLKRPT.......PLQALQHRYF |
| Sky1 | LTEKYKFSIQEAKDFEAFLLPMLNYLPEKRAT.......AKDCLNHTWL |
| DDB0230051 | ..............DAYNLLCALLSFNPTTRIK.......ANQAAFHKFF |
| DDB0230126 | IDNLRSHCSLASDSALDLLTQMLQFVPHKRIS.......IEQALSHPFI |
| Cdc7 | RSESSCDKQDVPVELYDLLERCLDPNPLTRIT.......ASEALLHPFL |
| DDB0229971 | QDDRPPLPDDISSELANFLERCFCKSTEERAT.......AKELLSHPWI |
| DDB0229896 | QDDCPPLPEGISPPLKDWLMQCFQKDPNLRIS.......AQKLLKHKWI |
| DDB0229877 | NNPHPPIPNNISADLKHFLLACFVRDINKRPT.......ASQLLEHPWI |
| DDB0231212 | QEDHPPIPPGISAALKDFLLNCFKKDENIRSS.......AKQLLHHPWV |
| DDB0229915 | SDQHPPFPTDISKEFLDYFQQSFKKDPTQRPT.......AQELLQHPIF |
| FNIPK-A | IHGKPKIPDHLSASCKNFIQRCLTIDPNHRDD.......IINLISHNFI |
| FNIPK-B | NIWIPKIPPHASKFLQNFIQKCFFEEEVLRFD.......SITLIDHPFL |
| FNIPK-C | TNFEPTIPYHLSNNCKNFIQKCLIQDPYHRWD.......TNLLLTHKFI |
| FNIPK-D | DFLFSKIPNHLVEKFKKVISQLLSKNINNQIL.......TDIISNKITT |
| FNIPK-Ea | .NGIPLIPDHLSNLFKNTIQHCLQINPNARFN.......ANELYNYVIK |
| FNIPK-Eb | DKGIPSIPTHLSNSFKNIIQNCLKFDTNARHS.......VESLIITLSN |
| DPYK4-a | FDGIPIIPNHVSEIMVDFIKRCLIIDPNKRSH.......MEELLTHRLI |
| ZakA-a | LNGIPIIPDHLSDTLKDFLNHCLVIDPKKRSY.......MEELLSHKLI |
| DDB0230002 | HIYEYVEKSQISPLFLNFIKKCI....GRYLD.......IDVLEIHPFL |
| DDB0229378 | PNNIPVFPVN.S.LFTEILNLCFQTEPSKRIK.......SHQLIKHPFF |
| Mek1 | KEEVPILPSTFSKEFRSFISECLQKEPTERPT.......ASNLLNHEFV |
| MEKKalpha | SNSIPNIPSHMSQEAFDFLNLCFKRDPKERPD.......ANQLLKHPFI |
| DDB0229911 | PSLEGDGESKWSHSFKDLVEKCLQKDPSKRPL.......PSKLLEHRFF |
| DDB0230012 | .LQEEEDKGVCSHLYKDLVDKCLQKEPSKRPN.......ASKLLEHKVF |
| MkcA | IKDPFEDPHKWSDEFHDIINKCLQMDPHKRPS.......AQELLKHPFI |
| MkcB | GATGFDDPALWSDCFKDFLSLCLKQDPAERAT.......AEELLKHPFI |
| MkcC | GCAGLDKPERWTEHFTHFLNLCLEMDPSKRST.......AEQLLKHPWI |
| MkcD | NLPLLLSTTTSSLEFRDFLTNCLQFDPSKRLT.......SSQLLQHPFL |
| MkcE | FSKEKKYSCHWSPELFDFLNVCLQMDPTKRPT.......PTQLLQHPFI |
| MkcF | GI..DFPKIRCSIDLKDMLWQCFESNPEKRST.......VDKLMRHPFF |
| PakA | GLPPIRDAHKWSKEFNDFLALCLEKDTEKRAS.......SSSLLHHPFL |
| PakB | GIPDLKDQNKWSDDFKDFVKKCLDKDVENRPE.......AKVLLNHPFL |
| PakC | GIPPLKETTKWSKTFQDFFSKCLDINVANRPD.......ATDLLKHPFM |
| PakD | GVPDFKEPNKWSSDFIEFVNLCLNLDDKRRPD.......AHYLLRHPFL |
| PakE | PPPKLSNPKDWSPCFNNFLATCLVKDPVQRPS.......VIDLLSHDFI |
| PakF | PPPTFAEPKKWSPLLNDFLARCLVKDPEKRAS.......PIDLLCHPFL |
| PakG | PPPTLSKPHHFSKELNDFIGQCCQKDPEKRPS.......AIELLTHPFL |
| PakH | PPPTFKDPKKWSPEFVSFVDKCLTKDINERWS.......PSQLLDHPFI |
| Krs1 | PPPKLTEPEKWSPEFNDFLAKCLTRKPELRPS.......AEELLKHPFI |
| DDB0216375 | TSPTLKEPHKWSPEFSDFIALCLAKEQSQRPS.......AKDLLKHSFF |
| SvkA | PPPTLE..GNFSKGFKEFCALCLNKDPNQRPT.......AKDLLKHKFI |
| DDB0216374 | QAPTLEVPGNWSPEFNDFISVCLNKEADKRPS.......AVDLLNHPFI |
| DDB0216379 | PPPSLKIPSDWSPEFNDFVKQCLSMNPALRPS.......AQQLLSHPFI |
| DDB0216377 | ESPALTDKSIWSDKFQDFISKCLTKDPAERPT.......AKELLNHEFI |
| DDB0216378 | DPPKLKNQENYSKDFINFIQSCLQKDPNQRKT.......ATELLKHPFV |
| DDB0230010 | .QYKLPNPKKYTKEFVDFLYLCLNPDPFKRPT.......PEMLLHHTFL |
| DDB0229972 | NCLDENEQSLFSLELNQFLELCFKKEPSDRAS.......VHDLLRHPWL |
| DDB0229973 | LPTDLIQPKLL.QPLIELLVTCIDLNPNHRPN.......ASQLVQKLTK |
| IfkA-a | ...KTHPSSPISNNAKDFLSLCFTINGNGSTNDS...LEAGILLKHPFL |
| SAMK-A | ........DNLTISCRLALGDIIGLEQNFKPD.......SKDLQKLSWF |
| SAMK-B | .........YLTLECKEVLNETLKYNQNFRAD.......SKQLLEAQWF |
| SAMK-C | .........GMSLECQILFNETTKIDSCFRPT.......SKQLLNFSWF |
| SAMK-D | ......SRDKVSFDYRSLFTEINKDEPCFRPS.......SKELLKSFWF |
| DDB0230038 | PLP.NCP.....FTIEKLIKLCLSTDPSVRPT.......FTTILKILRQ |
| DDB0230124 | EIPDNVP.....SVFSRLIKACWSADSSARPS.......FLTISKILSQ |
| DDB0229871b | TISNNMP.....NEISKILQQGWFSDSVLRPS.......LDTIIKELLI |
| DDB0229850 | SLPNICP.....EPLEKLYKQCVDQSPLNRPS.......CEEVIISLNQ |
| ARCK-1 | TIPIGVP.....QVLKDLIYCLWDHDVSSRPT.......ATEAMTALAV |

Subdomain XI

```
SAPKalpha     TIPNACP.....ESLVSLIQDCWDPNLENRPT.......CTDILSRLVT
DDB0229849    TLPQNTP.....PGLEALYKQCVNQEQTLRPS.......CEEIIETLNR
DDB0229848    TTPENAP.....ESLKNLIARTLVKDSTLRPT.......TLEFYEELLK
Phg2          KISENCP.....EQLKTLITRCWDENPDKRPN.......CDEILEILLT
DDB0229847    TIPQNCP.....EAFSNLISNCWESSPDNRPS.......CPEILDSLLD
SplA          PIPLTTS.....SKWKEILTQCWDSNPDSRPT.......FKQIIVHLKE
DPYK4-b       QIPFNVP.....LKFKELITQCWDRNPLNRPK......DFSEIIDKLKD
DDB0229872    PL.NGFP.....PYWVELISKCWNITPSLRPS.......FKEILQILNQ
DDB0229844    TMPPNVP.....PKLSYLIQKCWNQDPNERPS.......CQQILLAITS
ZakA-b        QIPFNVP.....LKFKELITQCWDRNPLNRPK......DFSIIIEKLKE
7TMK1         PMPQPMDPMW..ENLGKLIEMCWKKSPEERPS.......FSFILDFLEA
rk1           PVPQNGP.....PKYIQLLIDCLNENPSHRPT.......MEQCLERLES
rk2           PTPKYGP.....PKYIQLLKDCLNENPSQRPT.......MEQCLEILES
rk3           IVPPQVS.....APFTRLITECWSEDPQQRPS.......FQETVKRLEA
DDB0214883    PMPAWCP.....PKYAALMNRCWETDPTHRPS.......FPEILPIMEG
Shk1          SIPDDCP.....KSLKELIQKCWDPNPEVRPS.......FEGIVSELEE
Shk2          IIPDYFP.....TRLKDLLARCWDHYPSRRPS.......FAEISKQRIL
Shk3          PIPNDCL.....DSLRRLITEKCWDKEPISRPS.......FKEIISALDH
Shk4          PIPADTL.....PSLRHLIQTCWDHNPQNRPS.......FSEILFRLNE
Shk5          PIPDSFP.....KSLKTLIQKCWDHDPNKRPS.......FNEVTQRMNE
DDB0229963    ELPDNCD.....PNWKKLVVWCWSEDPNKRPS.......FEEITNYLTK
DDB0231199    KISSSVP.....NFISSLIKDCLHNSPKKRPT.......MNQIIQKLCN
DDB0229940    TIPADCP.....SEYSKLIKACWTDKPKKRPS.......FKEICDTLKK
DDB0218878e   TIPADCP.....SEYSKLIKACWTDKPKKRPS.......FKEICDTLKK
Rck1          ELPKDMQ.....TSISNIIKKCWQQKPSNRPS.......FIKIVAYLES
DDB0231197    TIPPTVP.....TTIANLIESCWVQTPHLRPT.......FTEILKVFYS
DPYK2         PIPEYCD.....PELKLLLTQCWEADPNDRPP.......FTYIVNKLKE
DPYK3-b       AIPPDCP.....VSLRKLITKCWASDPSQRPS.......FTEILTELET
Gdt1          SIGLDKI.D...PNIKNFIHKCW..NPIDGFT.......FNEILKTLKD
Gdt2          SLDQEIFKD...TDISDIVTRCWSKNPSQRPT.......FDYLSKYLKH
Gdt4          RLDQDVFAD...NDISLILLACWNPNPRGRPT.......FDTLIDLLEK
Gdt6          TLDLEKL.D...ADIRNIIERCWNQN..KRST.......FSEISISLKE
Gdt8          TADLDTI.D...DDIRKIIECCWIKEKSKRPS.......FSEISLHLEI
Gdt9          TKGLNSL.D...RDIKNLINICWA..PHSLPS.......FDEIVFSLVK
KinX          LIPTDCP.....ESLIDLTFNCCSVDPNNRPS.......FKEISQTLKQ
KinY          LIPDDIP.....VLYSDLALDCIKYQPEERPN.......FSHIIHVLKQ
DDB0229853    LAPESCP.....RAFMDLAKWCCSYNPKDRPT.......FKIVVEGLKV
DDB0229863    KVPKECP.....PDFSKLCVDCTKYNPKERPS.......FIKVLDTIQN
DDB0229866    LVPKDCP.....PPFLKLVLDCTEYDPDNRPT.......FKEITERLKS
DDB0229851    KVPADCP.....KELLRLVSECCKVAPSGRPS.......ASNILGLLEY
DDB0229865    KIPSECP.....REFLELSMKCCNYDPNDRPS.......FTDIVQTLDQ
GefX          KSIPGCP.....SELISVCVECCLYEPLTRPK.......FDEILSQLKV
DDB0229867    PSPNDCP.....IQFYDLALKCCSYKHTNRPM.......FSTIVTILES
HH498         PLPQTID.....HRWSNLIQICWDQNPNNRPS.......FDQILTIIQN
GbpC          TIPPTCP.....PEYVELIQSCWTHEPNSRPT.......FTSIVEILGQ
Pats1         KIPEDCP.....EMYSKLIVECWQTDASQRPK.......FSEIEDRLIK
QkqA          TLPFDCD.....PRLSNLIQLCWSTNPKKRPS.......FTFIIKELLE
Roco4         TIPEDCP.....PRLRNVIELCWSGDPKKRPH.......FSYIVKELSE
Roco5         TIPQNSVCGH..PDFITLITDCWQNDPLKRPT.......FIDIHSRLLI
Roco6         TISPSYTQTVTGHEYCGLIQDCWDSDIDRRPT.......FNKIVKRIKQ
Roco7         TIPNECP.....PDMKQLITDCWSGDPKKRPS.......FNSILERLST
Roco8         SINQNCN.....RMYSSLITKCWNADPNLRPS.......FNSILKTLNE
Roco9         SLPSHNL.....PEYDSLLNICWAQDPTCRPS.......FIEITKKLEE
Roco10        SIPLDCL.....PSFADLIKDCWSGEPLNRPS.......PSKIISQLYT
Roco11        TIPSDCP.....PTISNLIELCWSGDPKKRPH.......FSYIVKELTN
DPYK3-a       KIPTSCP.....LIIRKLINRCWAPLPSDRPT.......FNDILKLFDH
DDB0229956    PIPDSCL.....PEFRDLITMCWTSNPDDRPS.......FQQITTYLEN
DDB0229957    PIPMHCN.....KNYTDLMEQCWDDDPENRPY.......FDTIIHSISK
DDB0220138    EIPENCN.....EVLKNIIQDCWNQQSEDRPT.......FLSIYHRLEN
DDB0220436    HLPLDHLP....KGIQDLIRLCWKHDPSIRPS.......FIEIRSRLSE
DDB0230133    MIPPEIE.....GDLRKIIEACFHRDHTKRPN.......FENIEQFLDA
DDB0229871a   ELPSNCP.....QSFSDLIKECCNRNPDQRPN.......FSQILLKKL
DDB0229955    PIPNAWP.....YQLSHLIQACWHQDPLKRPS.......FTEILNLLNE

Bud32         ............SNSKIVIQKLNQVRLRGRKK.......TCFG......
DDB0231196    ............QSFVKLITRMCLPFRDRRPT.......VRQVLDNVKA
```

Subdomain XI

```
DDB0229339  ............QSLVKLITSMCLPCRDRRPT.......VHQILDEVGI
DDB0231195  ............SRLKRLLFLMMSKMPERRPS.......IKECIEEIQK
DDB0229335  ............DSFKEFLFSTMNRIYTCRPN.......SEECVERLIT
DDB0216331  ............EPIADLILDMLNLDQNLRPT.......IDLILQNKLF
DDB0229344  ............EKLAPLVLSMISKNPTFRPS.......IESIIETLQK
DDB0231281a ............PNVNQLIEFMCRHHENLKYT.........LLVSDFQH
DDB0230037a ...........NKINKILFEMMCSNESTERPT.......VDQLIEFMVC
DDB0219988a ............PSIDQLIEFVCRQYDQSRYT.......ISLSSYTHPN
DDB0231281b ...........SSKLKELVEKMGFFNYDDRIS.......LKELIECLNK
DDB0230037b ............TYLNDFIDNM.........T.......QPHVCKSFKK
DDB0219988b ............PSLVQIVKKMIKHKAF...............LLKQPTL
DDB0231182  SNRSKDNQSKLDPE.KRLLTK.LKKSMKDLP........PYKLLDHPFF
IksA        ............RDLKDMITILLRAKPHERPT.......ISQILSTHFI
IreA        ............PDIHQLVHSMIQFEPEKRPD.......IGECINHPFF
IrlA        ............APALDLVRLMISQDEKKRPT.......IDTILNHPLF
IrlB        ............QLACTLIKSMISKDESIRPT.......IQNVLNHPLF
IrlC        ............LVACDLISQMISKNESDRPT.......IEKILLHPFF
IrlD        ............LVACDLISQMISKNESDRPT.......TENILLHPFF
IrlE        ............LVATHLIELMISYDESKRPT.......LSSVLKHPLF
IrlF        ............LLACDLIKSMISNDESKRPT.......IENVLNHPLF
DDB0216373  PKPFPPSQTNYSNQFNQLIIDMVNLDKDERLH.......LNQVIERINQ
DDB0229347  .NYKIPDNSKYSNNFHKLIQFILVADPTQRPS.......INDLLNYLNE
DDB0229350  .NYTIPPNHTHSNDLISLIKIMLNPDPINRPN.......IFEITNQLNL
Nek2        .GRYNPIPSQYSEHLSKVISLMINVDPKSRPN.......VNELLGYSFI
Nek3        .GTYPPIPSHYSNDLRNLISSMLQIDPKNRPS.......VNDILELPFI
Nek4        .GQPLPISPNYSQDLQNLVYQLLEKQPTKRPS.......IFDIFQMPYI
DDB0229345  ............EELSSLLFKLLNKNPESRFS.......TQQILEQVFI
IfkA-b      ............PDQTQIIRSLLSRDPTQRPS.......TKQLLESGLL
IfkB        ............PDQTQIIRSLLSRDPTQRPS.......TKQLLESGLL
DDB0216407  ............SRQATLIRMLIDKDPAKRPS.......AQQLLQSELM
DDB0229432  ............PEESNLILRMIDINPDNRPT.......SDQILSELLP
DDB0220611e ............PKVSELILLMMKTNPDERPS.......ASDILKSDLF
DDB0220652  ............PNDTDFIDH.LIQSFATRPF.......SNQISTDYDN
PLK         ............HYGKSLIISILNPVPEQRPN.......LTQILEHDFF
Vps15       ............PNIQTLILHMIQKDPDQRYT.......PEKYISKWNT
TBCK        NSNIGIGLNGLGKELIEIIEQCLIPNPMDRPD.......SETLLDHPYF
TtkA        ............ENLLKVLKLCLIRNPHERPT.......IPTLLNHDFL
DDB0220004  ............TEFWQLIKSMIHPDPSVRPS.......AEQLLEHPLI
DDB0229384  ............DSLYQVILDMMKSNITERIS.......LDQILLNENI
DDB0216398  ............QDLKDLIILMMNPDHTKRIT.......IQSLLKYDKL
Scy1        ......................FAVKTESRLN.......PQKFLESPYF
Scy2        ............KVCTILLG.....DPMLRGD.......LENFIRSSFF
SLOB1       ............DKVFLLLQQIFS...EKTPT.......LEELVKNPWF
SLOB2       ............PEVRKVLEAIFKPFYGTPIT.......LEELSKMDFF
DDB0229334  ............TEQFDFLHSCLATNLSDRLD.......AQTLLHHPFI
DDB0231326  ............PAILDYLDYVFIRDPFYRPT.......LRDLLTKFIA
DDB0231559  ............QPLSDLIYSMLNHNEYDRPT.......SFEVFQKLKK
DhkG-a      ............LILSNIVNKLLQKSPDDRYQ.......SAYGLKKDLE
DDB0231198  ............IRIKTLIASLLAEQSQRKQS.......FNEVYNDLQD
DDB0231179  TQFTTITFDSKFNPIISQIINYIQ.YYRNRPN.......SSQVLESLIN
DDB0231335  ........HSKSYNDERQIEYIFAKRDNDKF...........LLNDKTF
7TMK2       ............EPVIQLYKSCTSDDPNKRPS.......SLGVKFHLAN
DDB0229381  ...KYGLVDPDIELGISFITSLLQLDPKKRLP.......LKETLNHPFL
Bub1        ............TGKLEIINQSIIQK..DSFS.......ELDTLWLPFL
DDB0230007  ............QHFNDLISKILEKNPQKRIT.......WPKLFIHPFF
DDB0229337  .....QFYKPENSLLIQLITCCLFKNPAQRIS.......WNEIVNHSFF
DDB0229346  .................................................
LvsG        .................................................
DDB0230125  ......................FQQAQNINYE.........EL.KNEFI
DDB0204911e ......................FQQAQDKNYE.........EL.KNEFI
CK1         .........GFPSEFATFLNYTRFLKFEDKPD.......FLYLRKLLRE
DDB0216336  ............PQFSQFMKHLKSLNYEDRPN.......YVFLQTLLND
```
