# Supplementary figures and images for: The Dictyostelium Kinome—Analysis of the Protein Kinases from a Simple Model Organism
Source: PLoS Genet. 2006 Mar 31;2(3):e38. doi: 10.1371/journal.pgen.0020038 (PMC1420674; doi:10.1371/journal.pgen.0020038)

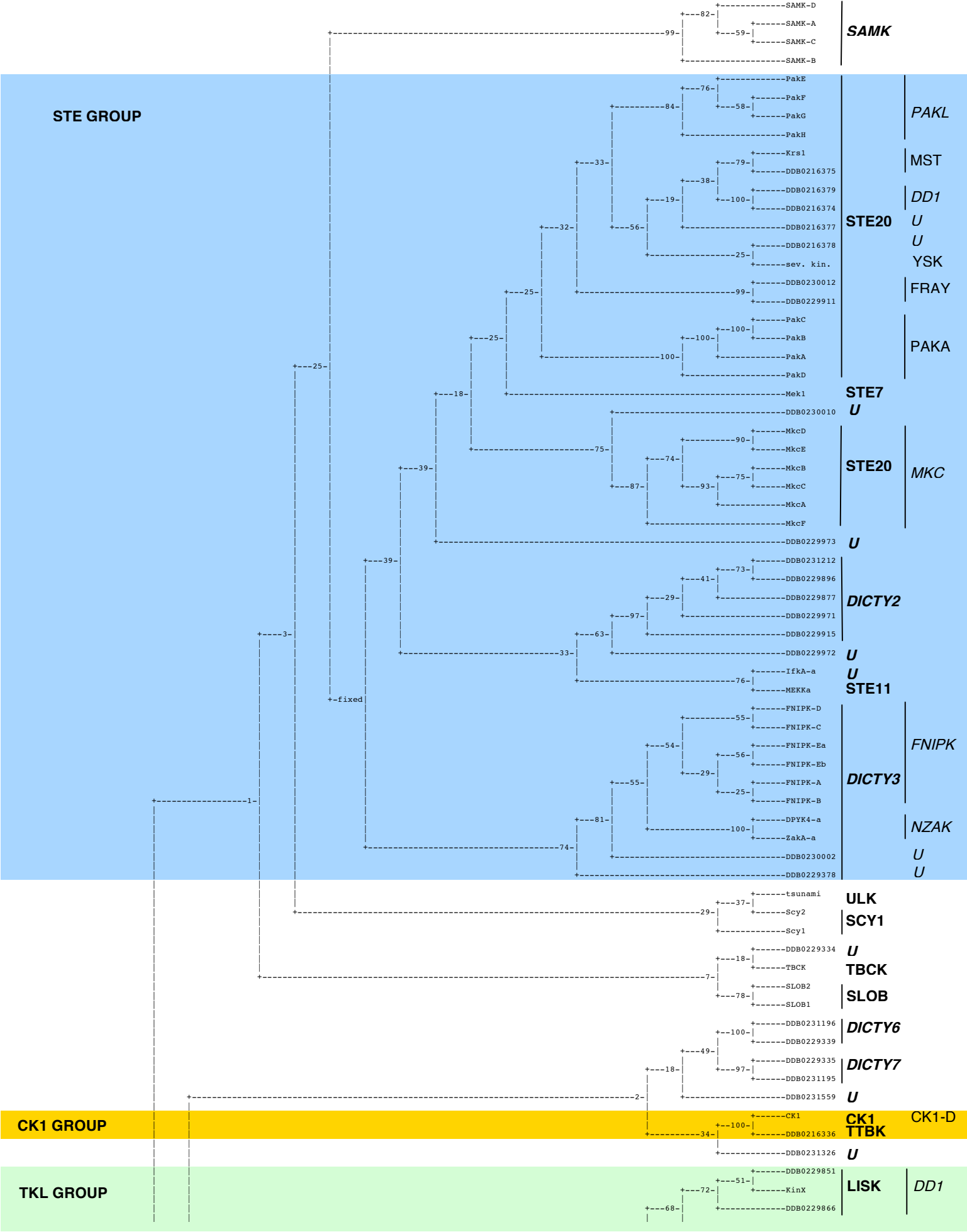

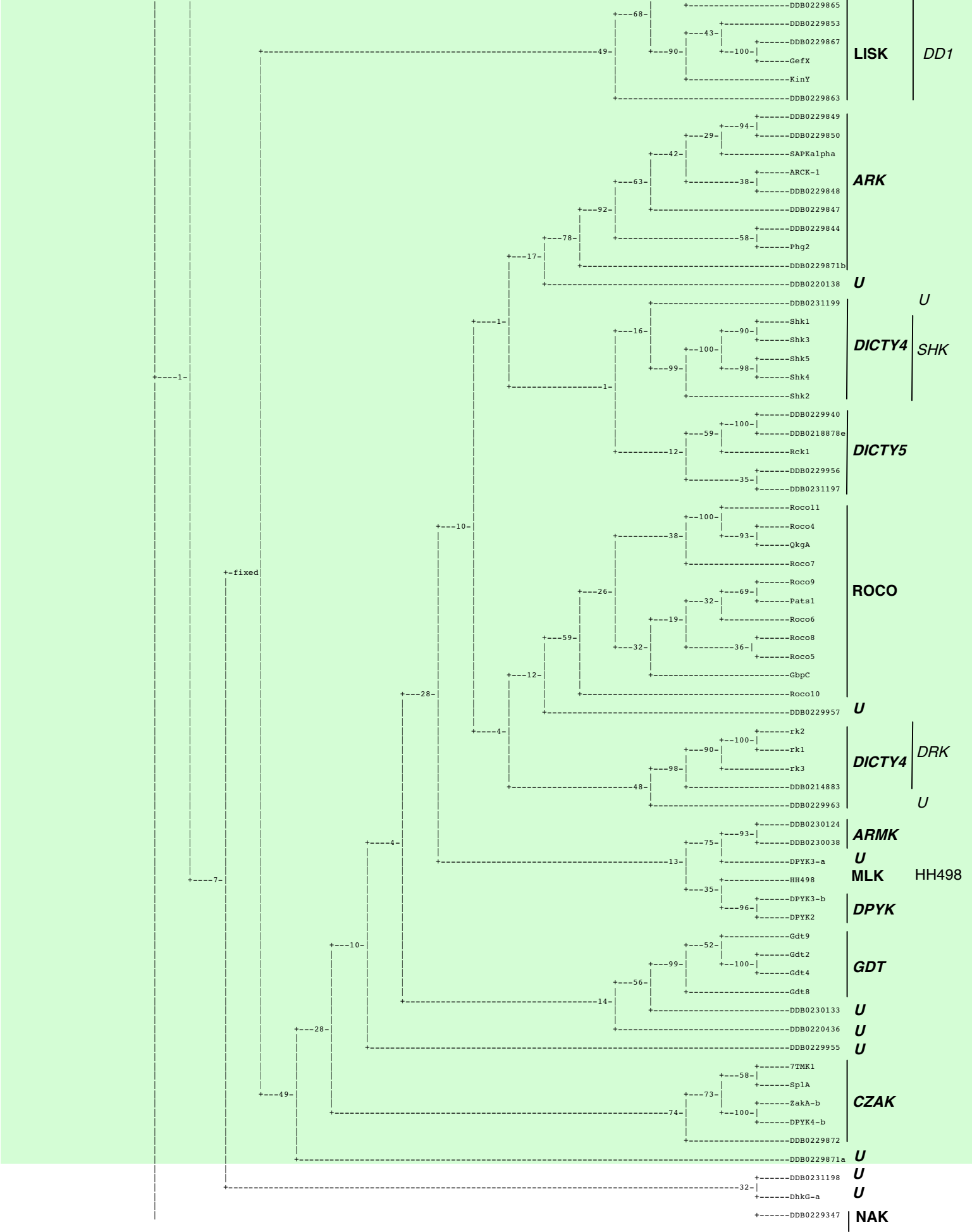

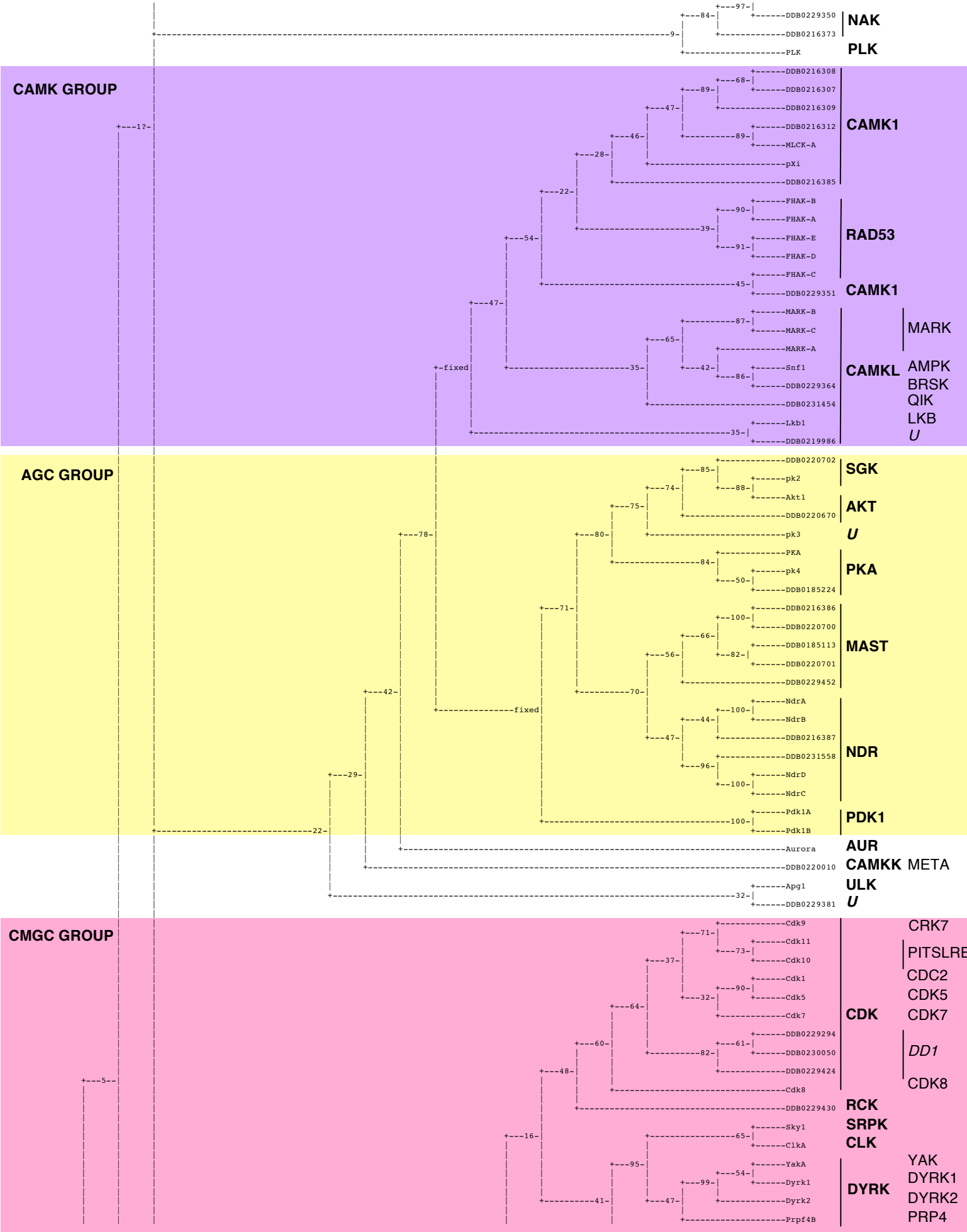

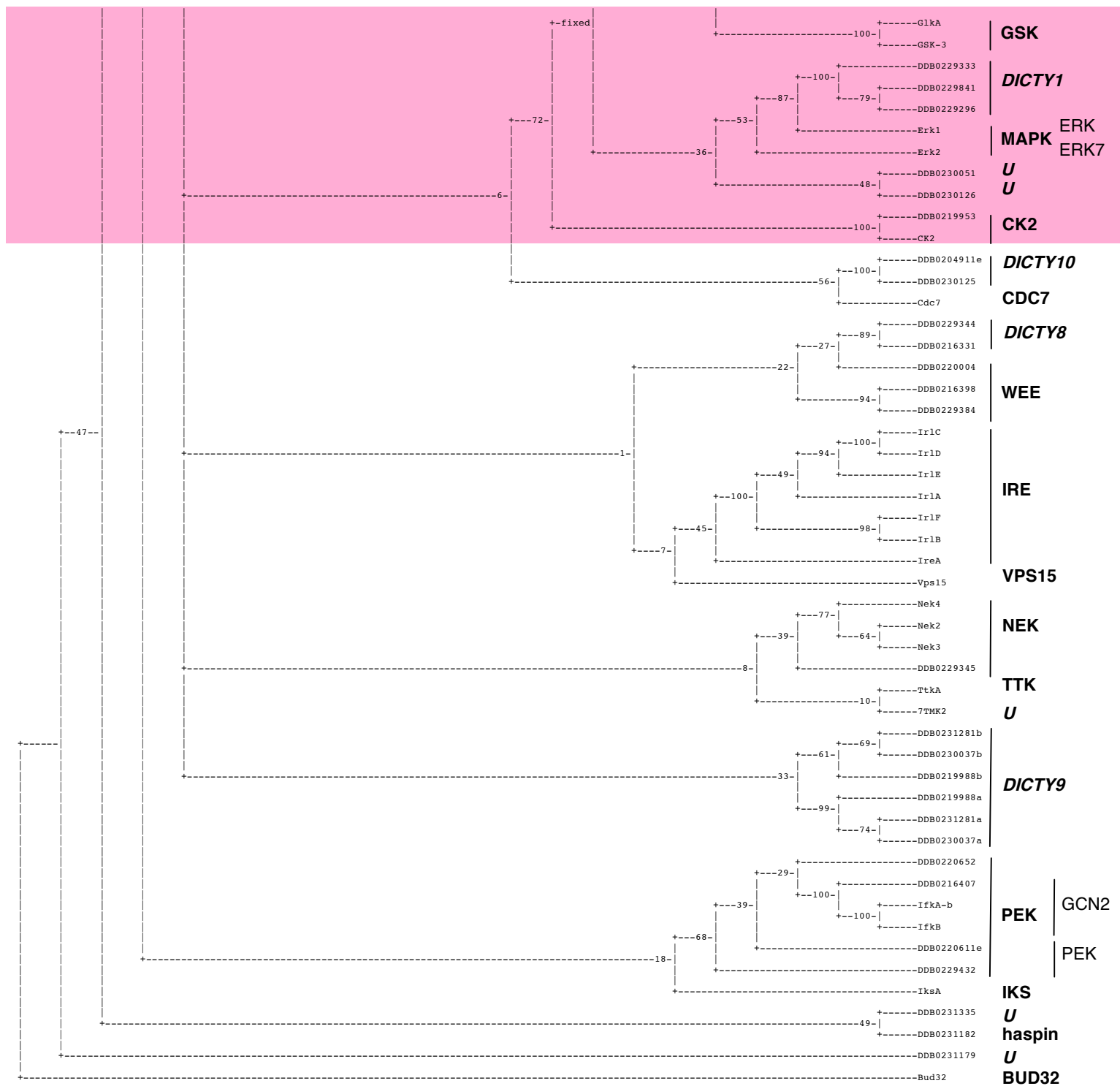

Supplement: Figure S2 — The tree shown in Figure 1 is shown here in text format with bootstrap values indicated at the nodes. Nodes at which group specific trees were grafted to the main tree are designated by the word “fixed.” (140 KB PDF) [file pgen.0020038.sg002.pdf]
