# Supplementary material for: The Dictyostelium Kinome—Analysis of the Protein Kinases from a Simple Model Organism
Source: PLoS Genet. 2006 Mar 31;2(3):e38. doi: 10.1371/journal.pgen.0020038 (PMC1420674; doi:10.1371/journal.pgen.0020038)
Supplement: Figure S3 — Matches to PFAM, SMART, and in-house HMMs, polyN and polyQ stretches (common in Dictyostelium proteins), transmembrane helices (TMDs), and signal peptides are shown. Motifs are labeled the first time they appear in each group, and the first time they appear on each page. All proteins are drawn to scale, and the vertical lines represent 100 aa intervals. A brief description of each of the motifs is given in the legend. (210 KB PDF) [file pgen.0020038.sg003.pdf]

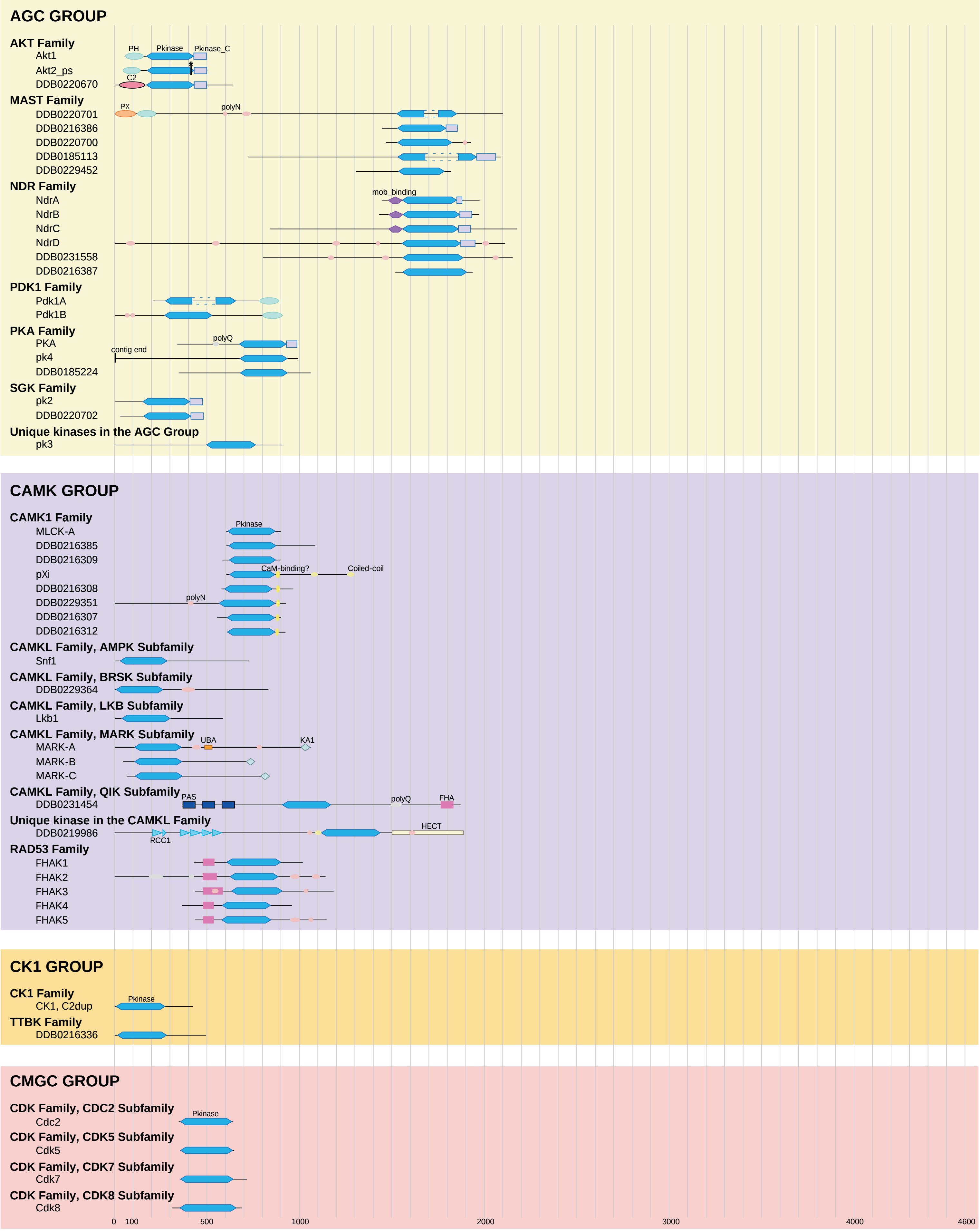
AGC GROUP
AKT Family
Akt1
Akt2_ps
DDB0220670
PH
Pkinase
Pkinase_C
C2
MAST Family
DDB0220701
DDB0216386
DDB0220700
DDB0185113
DDB0229452
PX
polyN
NDR Family
NdrA
NdrB
NdrC
NdrD
DDB0231558
DDB0216387
mob_binding
PDK1 Family
Pdk1A
Pdk1B
PKA Family
PKA
pk4
DDB0185224
polyQ
contig end
SGK Family
pk2
DDB0220702
Unique kinases in the AGC Group
pk3
CAMK GROUP
CAMK1 Family
MLCK-A
DDB0216385
DDB0216309
pXi
DDB0216308
DDB0229351
DDB0216307
DDB0216312
Pkinase
CaM-binding?
Coiled-coil
polyN
CAMKL Family, AMPK Subfamily
Snf1
CAMKL Family, BRSK Subfamily
DDB0229364
CAMKL Family, LKB Subfamily
Lkb1
CAMKL Family, MARK Subfamily
MARK-A
MARK-B
MARK-C
UBA
KA1
CAMKL Family, QIK Subfamily
DDB0231454
PAS
polyQ
FHA
Unique kinase in the CAMKL Family
DDB0219986
RCC1
HECT
RAD53 Family
FHAK1
FHAK2
FHAK3
FHAK4
FHAK5
CK1 GROUP
CK1 Family
CK1, C2dup
Pkinase
TTBK Family
DDB0216336
CMGC GROUP
CDK Family, CDC2 Subfamily
Cdc2
Pkinase
CDK Family, CDK5 Subfamily
Cdk5
CDK Family, CDK7 Subfamily
Cdk7
CDK Family, CDK8 Subfamily
Cdk8
0
100
500
1000
2000
3000
4000
4600

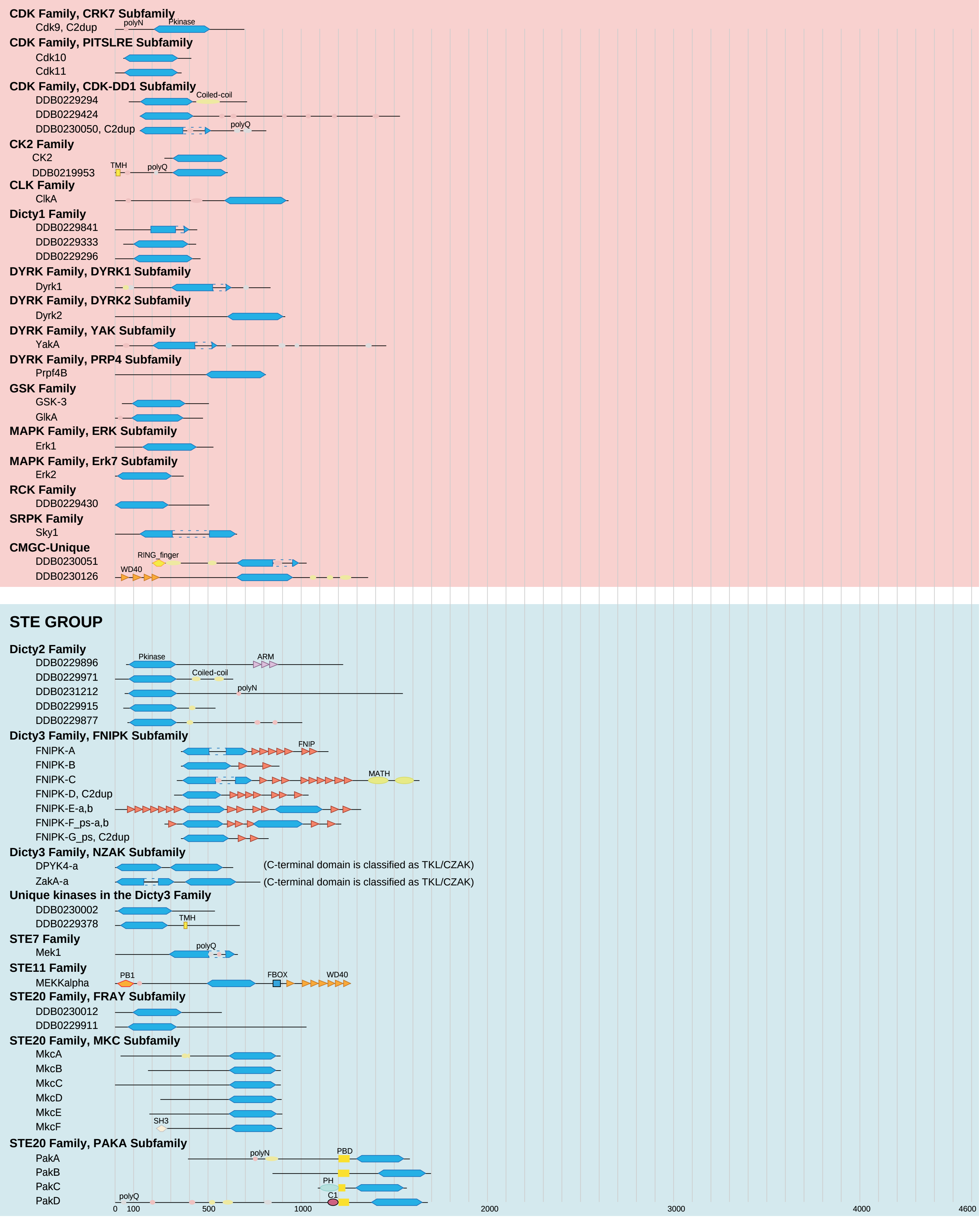

CDK Family, CRK7 Subfamily
Cdk9, C2dup
polyN
Pkinase
CDK Family, PITSLRE Subfamily
Cdk10
Cdk11
CDK Family, CDK-DD1 Subfamily
DDB0229294
Coiled-coil
DDB0229424
DDB0230050, C2dup
polyQ
CK2 Family
CK2
DDB0219953
TMH
polyQ
CLK Family
ClkA
Dicty1 Family
DDB0229841
DDB0229333
DDB0229296
DYRK Family, DYRK1 Subfamily
Dyrk1
DYRK Family, DYRK2 Subfamily
Dyrk2
DYRK Family, YAK Subfamily
YakA
DYRK Family, PRP4 Subfamily
Prpf4B
GSK Family
GSK-3
GlkA
MAPK Family, ERK Subfamily
Erk1
MAPK Family, Erk7 Subfamily
Erk2
RCK Family
DDB0229430
SRPK Family
Sky1
CMGC-Unique
DDB0230051
RING_finger
DDB0230126
WD40
STE GROUP
Dicty2 Family
DDB0229896
Pkinase
ARM
DDB0229971
Coiled-coil
DDB0231212
polyN
DDB0229915
DDB0229877
Dicty3 Family, FNIPK Subfamily
FNIPK-A
FNIP
FNIPK-B
FNIPK-C
MATH
FNIPK-D, C2dup
FNIPK-E-a,b
FNIPK-F_ps-a,b
FNIPK-G_ps, C2dup
Dicty3 Family, NZAK Subfamily
DPYK4-a
(C-terminal domain is classified as TKL/CZAK)
ZakA-a
(C-terminal domain is classified as TKL/CZAK)
Unique kinases in the Dicty3 Family
DDB0230002
DDB0229378
TMH
STE7 Family
Mek1
polyQ
STE11 Family
MEKKalpha
PB1
FBOX
WD40
STE20 Family, FRAY Subfamily
DDB0230012
DDB0229911
STE20 Family, MKC Subfamily
MkcA
MkcB
MkcC
MkcD
MkcE
MkcF
SH3
STE20 Family, PAKA Subfamily
PakA
polyN
PBD
PakB
PakC
PH
PakD
polyQ
C1
0
100
500
1000
2000
3000
4000
4600

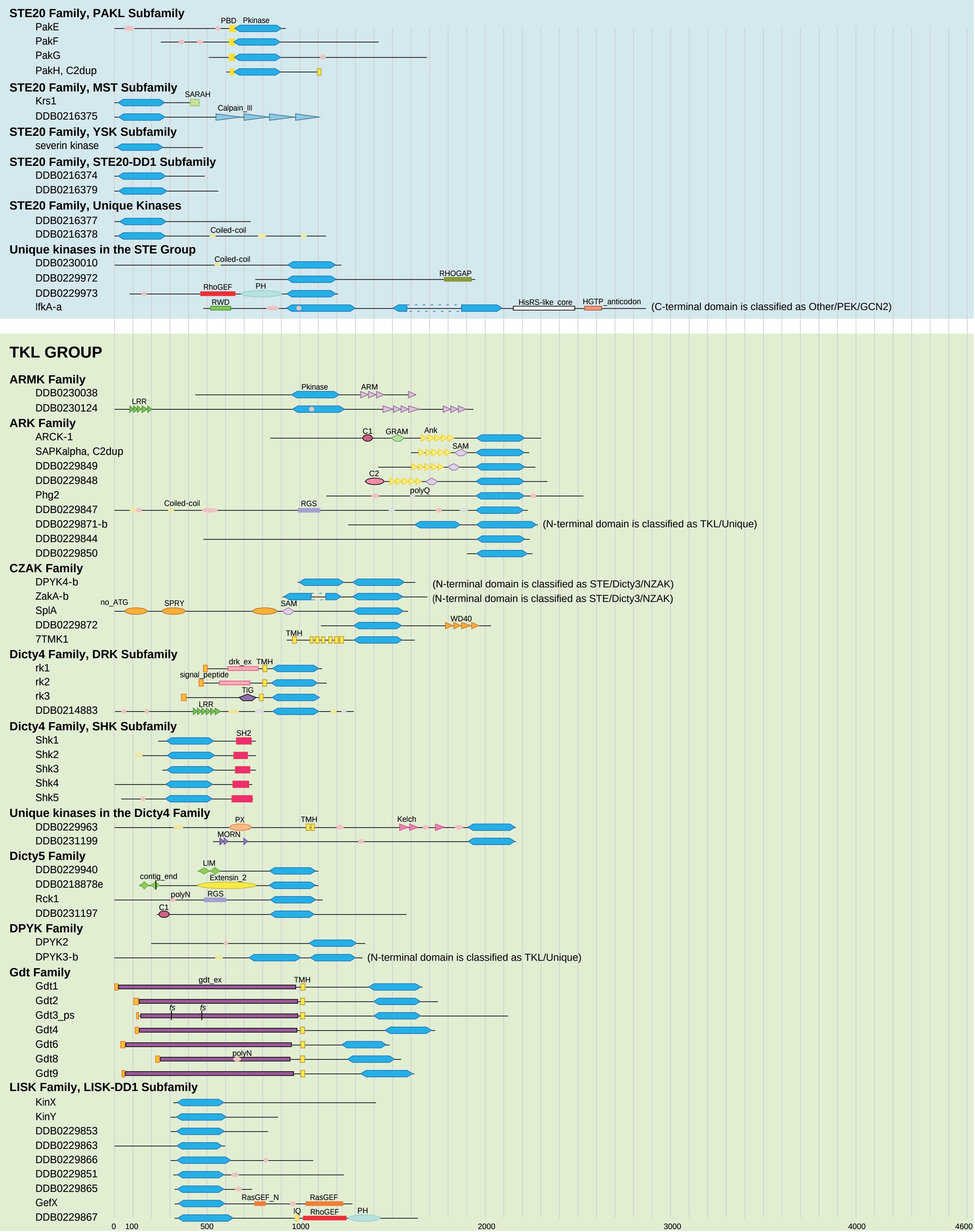

STE20 Family, PAKL Subfamily
PakE
PakF
PakG
PakH, C2dup
PBD
Pkinase
STE20 Family, MST Subfamily
Krs1
DDB0216375
SARAH
Calpain_III
STE20 Family, YSK Subfamily
severin kinase
STE20 Family, STE20-DD1 Subfamily
DDB0216374
DDB0216379
STE20 Family, Unique Kinases
DDB0216377
DDB0216378
Coiled-coil
Unique kinases in the STE Group
DDB0230010
DDB0229972
DDB0229973
IfkA-a
Coiled-coil
RHOGAP
RhoGEF
PH
RWD
HisRS-like_core
HGTP_anticodon
(C-terminal domain is classified as Other/PEK/GCN2)
TKL GROUP
ARMK Family
DDB0230038
DDB0230124
Pkinase
ARM
LRR
ARK Family
ARCK-1
SAPKalpha, C2dup
DDB0229849
DDB0229848
Phg2
DDB0229847
DDB0229871-b
DDB0229844
DDB0229850
C1
GRAM
Ank
SAM
C2
polyQ
Coiled-coil
RGS
(N-terminal domain is classified as TKL/Unique)
CZAK Family
DPYK4-b
ZakA-b
SplA
DDB0229872
7TMK1
(N-terminal domain is classified as STE/Dicty3/NZAK)
(N-terminal domain is classified as STE/Dicty3/NZAK)
no_ATG
SPRY
SAM
WD40
TMH
Dicty4 Family, DRK Subfamily
rk1
rk2
rk3
DDB0214883
drk_ex
TMH
signal_peptide
TIG
LRR
Dicty4 Family, SHK Subfamily
Shk1
Shk2
Shk3
Shk4
Shk5
SH2
Unique kinases in the Dicty4 Family
DDB0229963
DDB0231199
PX
TMH
Kelch
MORN
Dicty5 Family
DDB0229940
DDB0218878e
Rck1
DDB0231197
LIM
contig_end
Extensin_2
polyN
RGS
C1
DPYK Family
DPYK2
DPYK3-b
(N-terminal domain is classified as TKL/Unique)
Gdt Family
Gdt1
Gdt2
Gdt3_ps
Gdt4
Gdt6
Gdt8
Gdt9
gdt_ex
TMH
fs
fs
polyN
LISK Family, LISK-DD1 Subfamily
KinX
KinY
DDB0229853
DDB0229863
DDB0229866
DDB0229851
DDB0229865
GefX
DDB0229867
RasGEF_N
RasGEF
IQ
RhoGEF
PH
0
100
500
1000
2000
3000
4000
4600

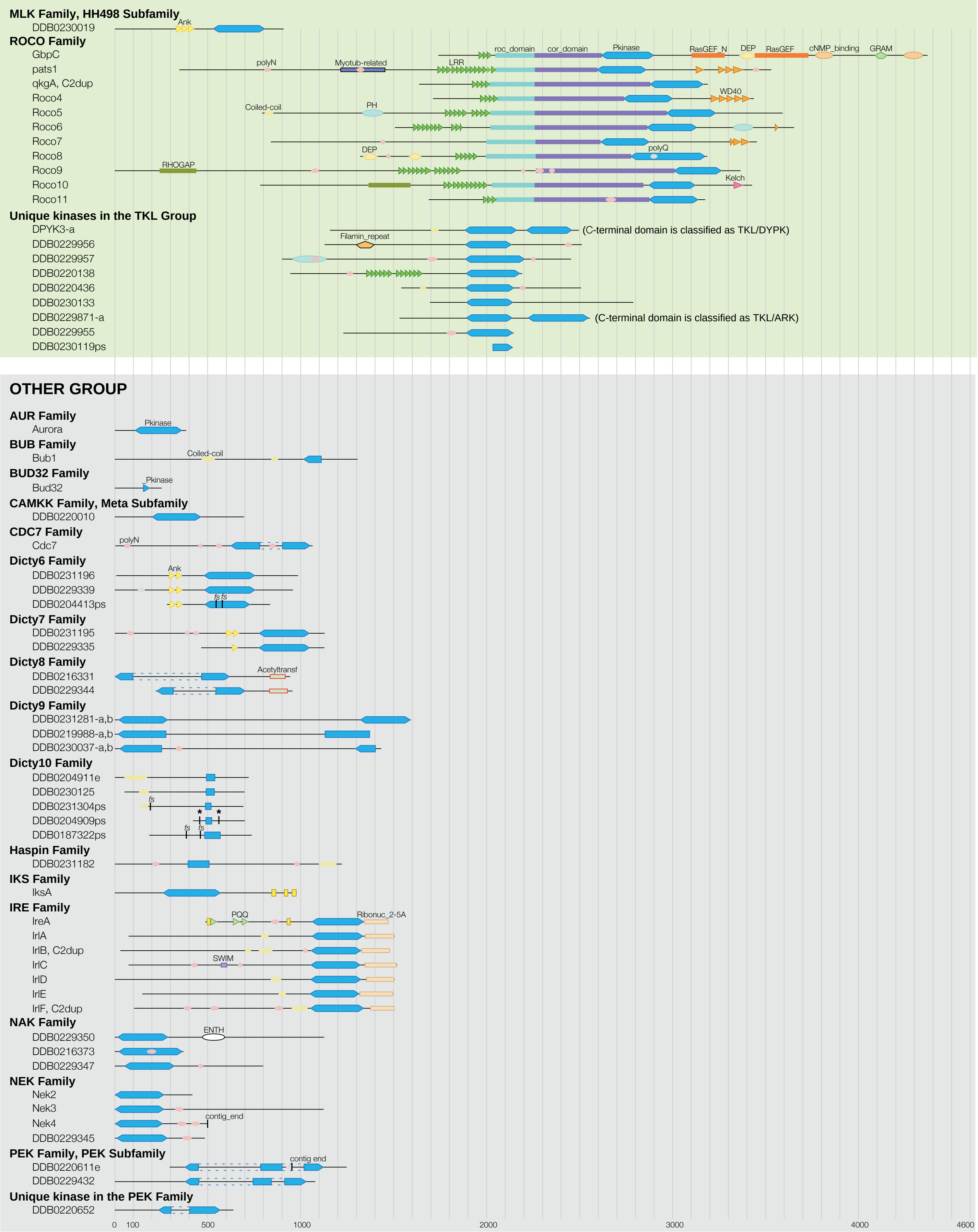

MLK Family, HH498 Subfamily
DDB0230019
Ank
ROCO Family
GbpC
pats1
qkgA, C2dup
Roco4
Roco5
Roco6
Roco7
Roco8
Roco9
Roco10
Roco11
roc_domain
cor_domain
Pkinase
RasGEF_N
DEP
RasGEF
cNMP_binding
GRAM
polyN
Myotub-related
LRR
WD40
Coiled-coil
PH
polyQ
RHOGAP
Kelch
Unique kinases in the TKL Group
DPYK3-a
DDB0229956
DDB0229957
DDB0220138
DDB0220436
DDB0230133
DDB0229871-a
DDB0229955
DDB0230119ps
(C-terminal domain is classified as TKL/DYPK)
Filamin_repeat
(C-terminal domain is classified as TKL/ARK)
OTHER GROUP
AUR Family
Aurora
Pkinase
BUB Family
Bub1
Coiled-coil
BUD32 Family
Bud32
Pkinase
CAMKK Family, Meta Subfamily
DDB0220010
CDC7 Family
Cdc7
polyN
Dicty6 Family
DDB0231196
DDB0229339
DDB0204413ps
Ank
fs fs
Dicty7 Family
DDB0231195
DDB0229335
Dicty8 Family
DDB0216331
DDB0229344
Acetyltransf
Dicty9 Family
DDB0231281-a,b
DDB0219988-a,b
DDB0230037-a,b
Dicty10 Family
DDB0204911e
DDB0230125
DDB0231304ps
DDB0204909ps
DDB0187322ps
fs
* *
fs fs
Haspin Family
DDB0231182
IKS Family
IksA
IRE Family
IreA
IrlA
IrlB, C2dup
IrlC
IrlD
IrlE
IrlF, C2dup
PQQ
Ribonuc_2-5A
SWIM
NAK Family
DDB0229350
DDB0216373
DDB0229347
ENTH
NEK Family
Nek2
Nek3
Nek4
DDB0229345
contig_end
PEK Family, PEK Subfamily
DDB0220611e
DDB0229432
contig end
Unique kinase in the PEK Family
DDB0220652
0 100 500 1000 2000 3000 4000 4600

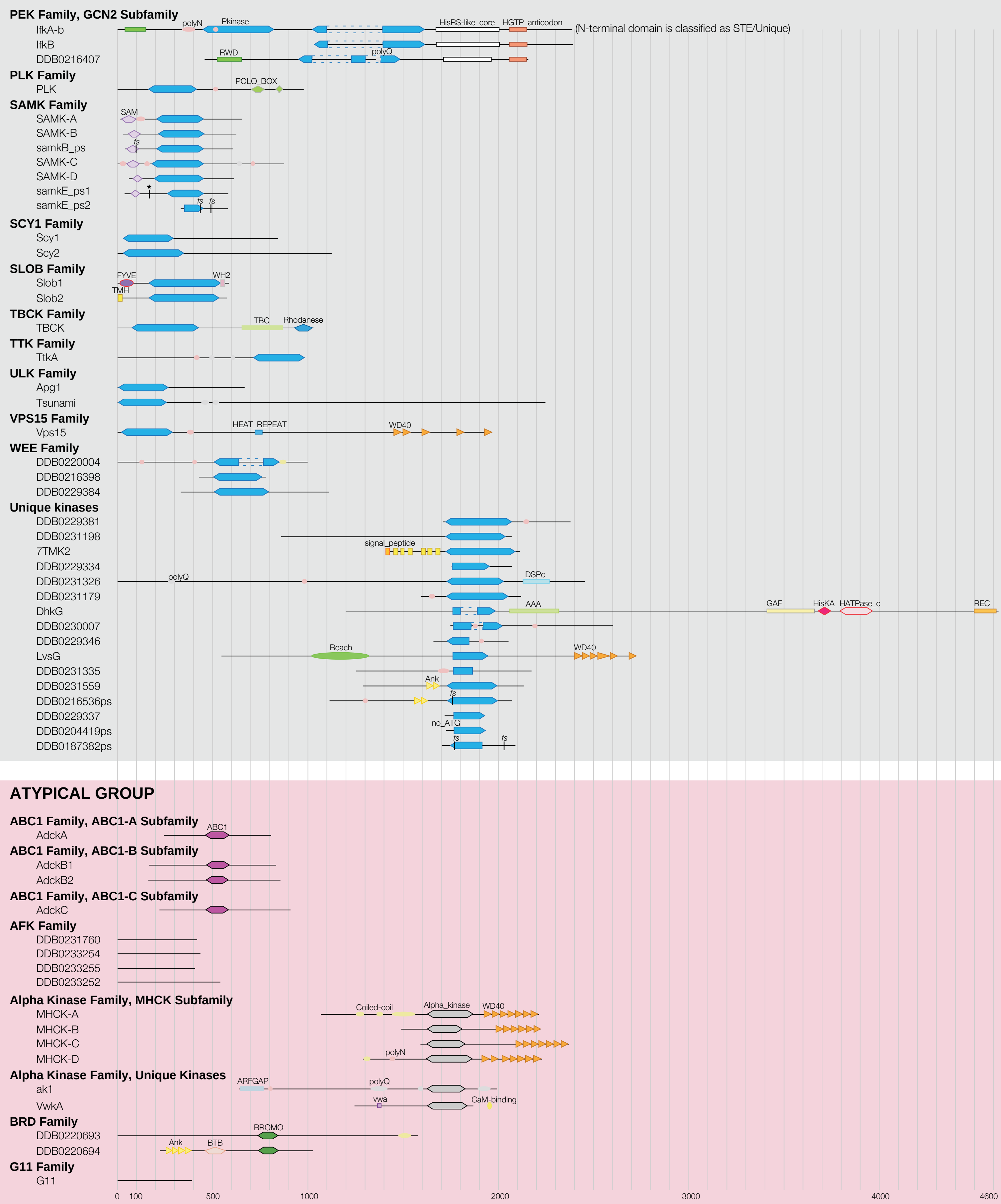
PEK Family, GCN2 Subfamily
IfkA-b
IfkB
DDB0216407
polyN
Pkinase
HisRS-like_core
HGTP_anticodon
(N-terminal domain is classified as STE/Unique)
RWD
polyQ
PLK Family
PLK
POLO_BOX
SAMK Family
SAMK-A
SAMK-B
samkB_ps
SAMK-C
SAMK-D
samkE_ps1
samkE_ps2
SAM
fs
SCY1 Family
Scy1
Scy2
SLOB Family
Slob1
Slob2
FYVE
WH2
TMH
TBCK Family
TBCK
TBC
Rhodanese
TTK Family
TtkA
ULK Family
Apg1
Tsunami
VPS15 Family
Vps15
HEAT_REPEAT
WD40
WEE Family
DDB0220004
DDB0216398
DDB0229384
Unique kinases
DDB0229381
DDB0231198
7TMK2
signal_peptide
DDB0229334
DDB0231326
polyQ
DSPc
DDB0231179
DhkG
AAA
GAF
HisKA
HATPase_c
REC
DDB0230007
DDB0229346
LvsG
Beach
WD40
DDB0231335
DDB0231559
Ank
DDB0216536ps
fs
DDB0229337
DDB0204419ps
no_ATG
DDB0187382ps
fs
ATYPICAL GROUP
ABC1 Family, ABC1-A Subfamily
AdckA
ABC1
ABC1 Family, ABC1-B Subfamily
AdckB1
AdckB2
ABC1 Family, ABC1-C Subfamily
AdckC
AFK Family
DDB0231760
DDB0233254
DDB0233255
DDB0233252
Alpha Kinase Family, MHCK Subfamily
MHCK-A
Coiled-coil
Alpha_kinase
WD40
MHCK-B
MHCK-C
MHCK-D
polyN
Alpha Kinase Family, Unique Kinases
ak1
ARFGAP
polyQ
VwkA
vwa
CaM-binding
BRD Family
DDB0220693
BROMO
DDB0220694
Ank
BTB
G11 Family
G11
0
100
500
1000
2000
3000
4000
4600

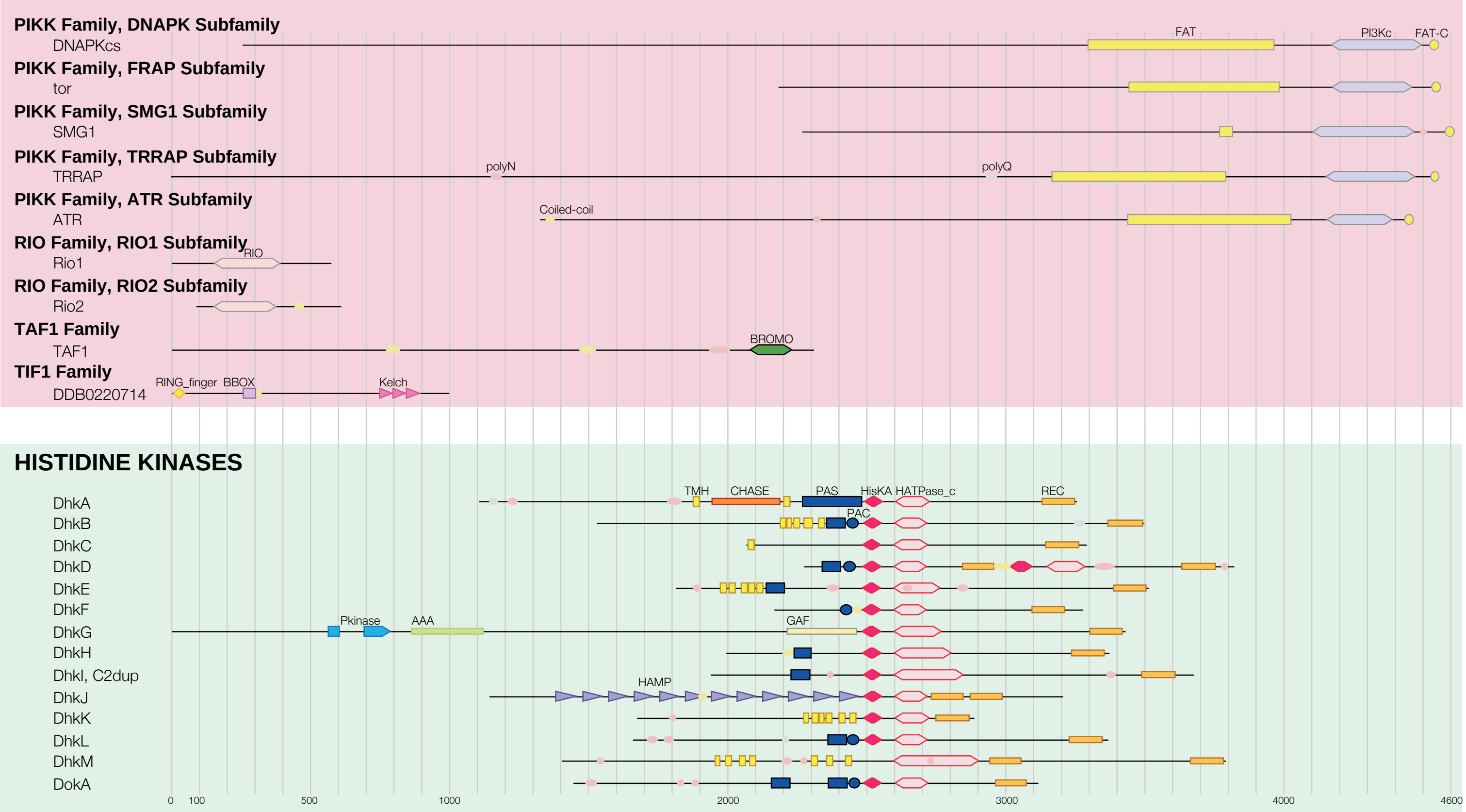
PIKK Family, DNAPK Subfamily
DNAPKcs
FAT
PI3Kc
FAT-C
PIKK Family, FRAP Subfamily
tor
PIKK Family, SMG1 Subfamily
SMG1
PIKK Family, TRRAP Subfamily
TRRAP
polyN
polyQ
PIKK Family, ATR Subfamily
ATR
Coiled-coil
RIO Family, RIO1 Subfamily
Rio1
RIO
RIO Family, RIO2 Subfamily
Rio2
TAF1 Family
TAF1
BROMO
TIF1 Family
DDB0220714
RING_finger
BBOX
Kelch
HISTIDINE KINASES
DhkA
DhkB
DhkC
DhkD
DhkE
DhkF
DhkG
DhkH
DhkI, C2dup
DhkJ
DhkK
DhkL
DhkM
DokA
TMH
CHASE
PAS
HisKA
HATPase_c
REC
PAC
Pkinase
AAA
GAF
HAMP
0
100
500
1000
2000
3000
4000
4600

LEGEND

KINASE CATALYTIC DOMAINS

Pkinase	Eukaryotic protein kinase (ePK) catalytic domain. Members phosphorylate serine/threonine and/or tyrosine.
Pkinase_C	Domain found downstream of many ePK domains from the AGC Group.
ADF	Actin Depolymerization Factor. Severs actin filaments and binds to actin monomers.
ABC1	Named for ABC1 from yeast, and is unrelated to the ABC transporter proteins.
PI3Kc	Phosphoinositide 3-kinase isoforms include lipid kinases and protein kinases.
Alpha_kinase	A novel protein kinase catalytic domain with a related structure, but no sequence homology, to ePKs.
RIO	Domain found in eukaryotes and prokaryotes that is related to ePKs.
BROMO	May be involved in protein-protein interactions in transcriptional activation complexes.
HATPase_c	Domain found in several ATP-binding proteins, including histidine kinases.
HisKA	Dimerisation and phosphoacceptor domain of histidine kinases.

EXTRACELLULAR DOMAINS

CHASE	Domain found in the extracellular portion of receptor-like proteins and is predicted to be a ligand binding domain.
gdt_ex	Extracellular domain found in *Dictyostelium* GDT proteins.
drk_ex	Extracellular domain found in two *Dictyostelium* protein kinases.
TIG	Domain found in cell surface receptors such as Met and Ron, as well as in intracellular transcription factors, where it is involved in DNA binding.

DOMAINS INVOLVED IN G-PROTEIN SIGNALING

ARFGAP	Putative zinc fingers with GTPase activating proteins (GAPs) towards the small GTPase Arf.
RHOGAP	GTPase activator proteins towards Rho/Rac/Cdc42-like small GTPases.
TBC	Thought to be a GTPase activator of Rab-like small GTPases.
RasGEF_N	Found N-terminal to the RasGEF domain in several exchange factors for Ras-like small GTPase.
RasGEF	Guanine nucleotide exchange factor for Ras-like small GTPases.
RhoGEF	Guanine nucleotide exchange factor for Rho/Rac/Cdc42-like GTPases.
RGS	Regulator of G Protein Signalling. Promotes GTP hydrolysis by the alpha subunit of heterotrimeric G proteins.
roc_domain	Ras-like domain of complex proteins.
cor_domain	Domain found C-terminal of roc_domain.
PBD	Domain that binds Cdc42p- and/or Rho-like small GTPases.

SECOND-MESSENGER-BINDING DOMAINS

CaM-binding?	Putative Ca^{2+}/calmodulin binding domain.
IQ	Ca^{2+}-independent calmodulin binding domain.
cNMP_binding	cAMP or cGMP binding site.
PH	Pleckstrin Homology. Binds inositol phosphates, and various proteins.
PX	Phox domain. Phosphoinositide-binding modules with varying lipid-binding specificities.
FYVE	Fab1, YOTB/ZK632.12, Vac1, and EEA1. Zn^{2+}-binding; binds PtdIns(3)P.
C1	Zn^{2+}-binding domain that may bind to molecules such as diacylglycerol and phorbol esters.
C2	Ca^{2+}-binding motifs that appears to bind phospholipids, inositol polyphosphates, and intracellular proteins.

REPEATED DOMAINS

Ank	Ankyrin repeats are usually involved in protein-protein interactions.
ARM	Armadillo repeats mediate protein-protein interactions.
PQQ	Domain found in several enzymes which utilise pyrrolo-quinoline quinone as a prosthetic group. Function is unknown.
Calpain_III	The function of domain III of calpains is unknown.
WD40	Named for their ~40 aa length, and because they often terminate in a WD dipeptide. Mediate protein-protein interactions, including to Gβ and myosin II.
FNIP	Named after the pattern of conserved residues. Found only *Dictyostelium*.
HAMP	Named for four of the proteins it is found in: histidine kinases, adenylyl cyclases, methyl binding proteins and phosphatases.
Kelch	Named after the protein in which it was first identified. Its function is unknown.
LIM	Named after Lin-11 Isl-1 Mec-3. Binds two Zn^{2+}; mediates protein-protein interactions.
LRR	Leucine-rich repeats provide a versatile structural framework for the formation of protein-protein interactions.
MORN	“Membrane Occupation and Recognition Nexus”. Function is unknown.
RCC1	Repeated domain found in regulator of chromosome condensation (RCC). Binds to chromatin and acts as a guanine-nucleotide dissociation stimulator for Ran.

STRUCTURAL MOTIFS

polyN	Regions at least 19 aa in length that are at least 90% asparagine.
polyQ	Regions at least 19 aa in length that are at least 90% glutamine.
Coiled-coil	Regions predicted to dimerize by forming parallel alpha-helices.
signal_peptide	
TMH	Transmembrane helix.

OTHER CATALYTIC DOMAINS

AAA	ATPase family associated with various activities. Often found in proteins with chaperone-like functions.
Acetyltransf	Gcn2-related N-actyltransferase. A superfamily that includes histone acetylases and other acetyltransferase enzymes.
HECT	Homologous to the E6-AP Carboxyl Terminus. E3 ubiquitin-protein ligase.
tRNA-synt_2b	tRNA synthetase class II core domain. tRNA synthetases catalyse the attachment of an amino acid to its cognate tRNA.
Myotub-related	Domain in myotubularin-related proteins. Myotubularin is a lipid phosphatase that dephosphorylates PtdIns(3)P and PtdIns(3,5)P_2.
DSPc	Dual-specificity (Ser/Thr and Tyr) protein phosphatases.
Ribonuc_2-5A	An endoribonuclease that cleaves an intron from Hac1 mRNA in humans, which causing it to be more efficiently translated.

PHOSPHOAMINO ACID-BINDING DOMAINS

SH2	Src homology 2. Binds phosphotyrosine-containing polypeptides
FHA	Forkhead-associated. Binds phosphopeptides. Highest specificity for phospho-threonine, but also recognises phosphotyrosine.

DOMAINS MEDIATING PROTEIN-PROTEIN INTERACTIONS

BTB	BR-C, ttk and bab. Mediates homomeric, and in some instances heteromeric, dimerization.
FBOX	A motif found in cyclin-F. Serves as a link between a target protein and a ubiquitin-conjugating enzyme.
Filamin_repeat	These form a rod-like structure in filamin, which is an actin-binding protein.
SH3	Src homology 3. Binds to target proteins through sequences containing proline and hydrophobic amino acids.
PB1	Phox and Bem1p domain. This is present in many eukaryotic cytoplasmic signalling proteins. Forms heterodimers.
mob_binding	Binding site for Mob1. NDR Family kinases are activated by Mob1binding.
Rhodanese	Rhodanese is a sulfuryltransferase with two Rhodanese domains. Inactive versions are found in dual specificity phosphatases and ubiquitin hydrolases.
RING_finger	A specialised type of Zn-finger. It probably mediates protein-protein interactions and probably has E3 ubiquitin-protein ligase activity.
DEP	dishevelled, Egl-10, and pleckstrinproteins. Unknown function.
SAM	Sterile Alpha Motif. Form homo- and hetero-oligomers. Also bind to non-SAM domain-containing proteins with a low affinity constant, and appear to bind RNA.
SARAH	Sav/Rassf/Hpo - 3 classes of eukaryotic tumour suppressors that it is found in. Mediates homodimerization.
WH2	Wiskott Aldrich syndrome homology region 2. Mediates actin-binding.
POLO_BOX	Found in Polo kinases. Mediates interaction with multiple proteins, some of which are substrates.

OTHER FUNCTIONS

HGTP_anticodon	Found in Histidyl, Glycyl, Threonyl and Prolyl tRNA synthetases. It is probably the anticodon binding domain.
PAC	Occurs C-terminal to a subset of PAS motifs, and is proposed to contribute to the PAS domain fold.
PAS	Found in Per, Arnt, Sim. Mediate protein-protein interactions, dimerization, and sensory functions such as detecting light and redox.
REC	Receiver domain. Contains a phosphoacceptor site that is phosphorylated by histidine kinases.

UNKNOWN FUNCTION

BBOX	Zn finger. Found in transcription factors, ribonucleoproteins and proto-oncoproteins, but no function is clearly assigned to this domain.
SWIM	Zn-finger.
Beach	Found in the BEIGE and CHS protein. The function is unknown. Usually followed by WD repeats.
RWD	Found in proteins containing Ring finger and WD40 repeat domains.
Extensin_2	Homologous hydroxyproline-rich glycoproteins (HRGPs) found in the plant extracellular matrix.
ENTH	Epsin N-terminal homology. Found in proteins involved in endocytosis and cytoskeletal machinery. May bind PtdIns(4,5)P2 and PtdIns(1,4,5)P3.
FAT	FRAP, ATM and TRRAP. Present in the PIK-related protein kinases.
FAT-C	Found at the C-terminal end of the PIK-related protein kinases.
GAF	Found in phytochromes and cGMP phosphodiesterases (PDEs). In PDEs, it forms an allosteric cGMP binding site.
KA1	Found in the C-terminal extremity of kinases in the MARK subfamily.
GRAM	Found in glucosyltransferases, myotubularins and other putative membrane-associated proteins.
HEAT_REPEAT	Found in Huntingtin, EF3, PP2A regulatory subunit, and yeast TOR1. Related to armadillo repeats.
MATH	Meprin And TRAF-Homology. TRAFs are intracellular proteins, and meprins are extracellular.
UBA	Ubiquitin associated domain. Found in several proteins having connections to the ubiquitination pathway.
SPRY	Found in SplA and Ryanodine Receptor.
vwa	von Willebrand factor type A. VWA domains in extracellular eukaryotic proteins mediate adhesion. Their function in intracelllular proteins is unknown.
